# Phage therapy against methicillin-resistant *Staphylococcus pseudintermedius*: a novel strategy for canine pyoderma

**DOI:** 10.3389/fmicb.2025.1719973

**Published:** 2026-01-13

**Authors:** Xiancheng Feng, Qingjie Du, Keyi Wang, Ruiqi Chen, Rui Ma, Yunping Xing, Xinyue Lei, Miao Wang, Pengxiu Dai, Xinke Zhang

**Affiliations:** The College of Veterinary Medicine, Northwest Agriculture and Forestry University, Yangling, Shaanxi, China

**Keywords:** methicillin-resistant *Staphylococcus pseudintermedius*, multidrug resistance, phages, pyoderma, therapy

## Abstract

**Introduction:**

Methicillin-resistant *Staphylococcus pseudintermedius* (MRSP) poses a major public health challenge due to its multidrug resistance. Phages represent an innovative therapeutic strategy with considerable potential against drug-resistant bacterial infections.

**Methods:**

In this study, dogs clinically diagnosed with pyoderma were recruited to establish an epidemiological dataset, and multidrug-resistant *S. pseudintermedius* strains (including 6 MRSP isolates) were obtained from bacterial cultures. Three high-efficiency lytic phages were isolated using these MRSP strains as hosts and characterized. The combined efficacy of phages and antibiotics was evaluated in a pyoderma model via clinical scoring, histopathological examination, and tissue bacterial load quantification.

**Results:**

Notably, these phages enhanced MRSP susceptibility to antibiotics, with genomic and proteomic analyses identifying key mediators of phage-mediated *S. pseudintermedius* lysis. The phage-antibiotic combination exhibited the most significant therapeutic effect on pyoderma, followed by the phage-only group. In contrast, the antibiotic-only group showed no significant improvement compared to the control group, with both yielding poor outcomes.

**Discussion:**

This study provides a promising therapeutic strategy for the clinical management of canine pyoderma in veterinary medicine and offers valuable insights for safeguarding public health security.

## Introduction

Canine pyoderma is a common bacterial skin infection that severely threatens pet health. Moreover, cross-infections—both among animals and between animals and humans—have exerted an impact on public health and safety. Antibiotic therapy remains the mainstay for treating canine pyoderma, but the misuse and overuse of antibiotics in veterinary medicine have accelerated the development of bacterial resistance, posing considerable challenges to clinical management. Notably, canine pyoderma often presents as mixed infections involving multiple bacterial species, with *Staphylococcus* spp. playing a key role in disease progression (Hnilica and May, 2004; Shobha et al., 2024). Research has indicated that *S. pseudintermedius* is the predominant pathogenic bacterium associated with canine pyoderma and surgical site infections (Lee et al., 2023).

Currently, reports on MRSP are increasing emerging globally, making it a prevalent pathogen with pandemic potential. Notably, MRSP harbors aminoglycoside, macrolide, and tetracycline resistance genes, which confer a multidrug-resistant phenotype, rendering it resistant to most clinically available antibiotics and posing a significant threat to animal health (Morais et al., 2023; Wormhoudt et al., 2017; Worthing et al., 2018). Companion animals play an important role in the epidemiology of MRSP: as close human companions, dogs engage in frequent intimate contact with humans, and MRSP exhibits a high propensity for interspecies transmission (Moses et al., 2023). Consequently, several studies have hypothesized that pets may serve as a reservoir for drug-resistant bacteria, with implications for public health (Srednik et al., 2023). Current research has confirmed that animal-derived *S. pseudintermedius* can be transmitted to and infect humans (Binek et al., 2019), posing a threat to human health and public safety. Thus, increased attention should be directed toward antimicrobial resistance in animal-derived bacteria.

Phages—viruses that specifically infect bacteria, are widely distributed in nature and the human microbiota, and are regarded as the most abundant biological entities on Earth (estimated at ∼10^31^)—remain largely undercharacterized, with only tens of thousands of phages isolated, identified, and sequenced to date (Espinoza et al., 2025). In recent years, the widespread use of antibiotics has led to the continuous emergence of drug-resistant bacteria, greatly exacerbating the challenges in treating bacterial infectious diseases. Phages exhibit considerable potential for mitigating the threat posed by these superbugs: they are unaffected by antibiotic resistance mechanisms, and depolymerases and lysins encoded by phages facilitate the disruption of cell walls and biofilms in highly virulent bacteria (e.g., mucoid strains). Thus, phages offer a novel therapeutic approach for bacterial infections and represent a promising strategy for addressing multidrug-resistant bacteria. In recent years, clinical trials of phage therapy have been successively launched in Europe, the Americas, and China, where targeted phages are used to treat superbug infections, yielding important progress (Bao et al., 2020; El Haddad et al., 2019; Elfadadny et al., 2025; Rohde et al., 2018).

Given the clinical challenges posed by MRSP in canine pyoderma and the associated public health risks, this study aimed to explore novel therapeutic strategies for veterinary clinical management of the disease. By investigating phage-based interventions and their potential applications, this research sought to provide valuable insights for addressing antibiotic resistance in animal-derived bacteria and safeguarding public health security.

## Materials and methods

### Epidemiological investigation and sample collection of canine pyoderma

Skin samples from canine pyoderma cases were collected from multiple pet hospitals in Xi’an City, Shaanxi Province, China. Eligible subjects were dogs presenting with clinical manifestations including skin papules, erythema, and purulent exudation upon physical examination. For sample collection, hair surrounding the lesions was clipped, and a sterile surgical blade was used to gently scrape the junction of the lesion and healthy skin until slight bleeding occurred. The collected skin scrapings were immediately transferred to sterile microcentrifuge tubes containing 1 mL of sterile normal saline. Concurrently, microscopic examinations were performed on all samples. Relevant clinical data of affected dogs, including gender, age, breed, onset time, and pyoderma type, were systematically recorded and subjected to statistical analysis.

### Isolation, cultivation, and identification of pathogenic bacteria

The collected samples were inoculated into brain-heart infusion (BHI) broth (Guangdong Huankai Microbiological Technology Co., Ltd., China) and subjected to enrichment culture at 37°C with shaking at 200 rpm for 6 h. Subsequently, they were inoculated onto Columbia blood agar medium (Guangdong Huankai Microbiological Technology Co., Ltd., China) and BHI agar medium (Guangdong Huankai Microbiological Technology Co., Ltd., China), and incubated at 37°C for 18–24 h. Single colonies were picked and streaked onto new Columbia blood agar medium for purification for 3–4 generations until the colony sizes were consistent. The obtained colonies underwent Gram staining and 16S rDNA PCR detection. Single colonies were inoculated into BHI broth and incubated at 37°C and 200 rpm. The OD value was determined every 1 h starting from 0 h for a total of 15 h to calculate the logarithmic growth phase of the bacteria.

### Drug susceptibility test of pathogenic bacteria

Antimicrobial susceptibility test was performed using drug susceptibility test strips, following the Clinical and Laboratory Standards Institute (CLSI) guidelines for veterinary pathogens. Specifically, the Kirby-Bauer (K-B) agar diffusion method recommended in CLSI VET01 was employed, and test results were interpreted strictly in accordance with the minimum inhibitory concentration (MIC) breakpoints specified in CLSI VET01S. The concentration of the bacterial suspension was adjusted to an absorbance value of 0.08–0.13 at OD_600_ nm. A sterile cotton swab was utilized to dip into the bacterial suspension and evenly spread onto the MHA agar plates (Guangdong Huankai Microbiological Science and Technology Co., Ltd., China). The drug susceptibility test discs were adhered to the medium coated with the bacterial suspension, and the medium was inverted and incubated in a 37°C constant temperature for 16–18 h.

All commercial antimicrobial susceptibility paper discs were purchased from Hangzhou Binhe Microbial Reagent Co., Ltd. (Hangzhou, China). The discs used in this study included: penicillin G (10 IU); oxacillin (1 μg); ampicillin (10 μg); amoxicillin-clavulanate potassium (10 μg); gentamicin (10 μg); enrofloxacin (10 μg); trimethoprim-sulfamethoxazole (23.75/1.25 μg); clindamycin (2 μg); doxycycline (30 μg); rifampicin (5 μg); and florfenicol (30 μg).

### Isolation, purification, and concentration of phages

Sewage samples (vicinity of the Animal Hospital and Laboratory Animal Center, Northwest A&F University) and Yangling River water samples (Shaanxi, China) were pre-filtered with multi-layered gauze, centrifuged (10,000 × g, 10 min, 4°C), and the supernatant filtered through a 0.22 μm membrane (Millipore, United States) to obtain sterile filtrate. Logarithmic-phase *S. pseudintermedius* strains (strains 1, 26, and 32; BHI broth, 37°C, 200 rpm) were each mixed with equal volumes of sterile filtrate in BHI broth, statically incubated for 15 min, and cultured overnight (37°C, 200 rpm). The mixture was centrifuged (10,000 × g, 10 min, 4°C), and the supernatant was filtered through a 0.22 μm filter membrane to obtain the phage lysate.

Plaque assay was used to purify and titrate phages: serial 10-fold dilutions of lysate in BHI broth were mixed with 100 μL logarithmic-phase *S. pseudintermedius* and BHI semi-solid medium (10 g/L agar), poured onto BHI solid agar plates (15 g/L agar), and stood for 30 min. Then it was inverted and incubated overnight at 37°C. A single phage plaque was resuspended in SM buffer (Biosharp, China), and incubated at 4°C overnight. Three rounds of single-phage purification yielded phage plaques with uniform morphology and size.

*S. pseudintermedius* was cultured in 100 mL BHI broth (37°C, 200 rpm) to OD600 = 0.08–0.13. After adding 1 mL purified phage suspension, the mixture was incubated (37°C, 180 rpm) until clear (complete lysis), centrifuged (10,000 × g, 10 min, 4°C), and the supernatant filtered (0.22 μm filter membrane). DNase I and RNase A (Sigma-Aldrich, United States) were added to the filtrate (1 μg/mL each) and incubated (37°C, 30 min). NaCl was added to 1 mol/L, incubated on ice for 1 h, centrifuged (10,000 × g, 30 min, 4°C), and PEG 8,000 added to the supernatant for overnight incubation at 4°C. The sample was centrifuged (12,000 × g, 30 min, 4°C), the supernatant discarded, and the pellet resuspended in 1 mL SM buffer. Chloroform (1 mL) was added, vortexed for 1 min, centrifuged (4,000 × g, 15 min, 4°C), and the upper aqueous phase collected. Chloroform extraction was repeated 3–4 times for purification. The purified phage suspension was mixed with glycerol and stored at −80 °C.

### Determination of the biological characteristics of phages

The purified phages were characterized by transmission electron microscopy observation, optimal multiplicity of infection (MOI) determination, one-step growth curve assay, host range analysis, thermal stability evaluation, and pH stability assessment.

Electron microscopic observation: 20 μL of the purified phage solution was dropped onto a copper grid and allowed to stand for 10 min. Subsequently, 10 μL of 2% phosphotungstic acid (Sigma-Aldrich, United States) was added and allowed to stand for an additional 10 min. After drying, the samples was observed using a transmission electron microscope (Hitachi, H-600IV, Japan).

Determination of the optimal MOI: The target bacteria in the logarithmic growth phase were adjusted to a concentration of 1 × 10^8^ CFU/mL. The phage suspension was mixed with the target bacterial culture at MOI values of 10^–5^, 10^–4^, 10^–3^, 10^–2^, 10^–1^, 1, 10, and 100. The mixture was inoculated into BHI broth and incubated at 37°C and 200 rpm for 8 h. Following incubation, the cultures were centrifuged at 5,000 × g for 15 min at 4°C to remove bacterial debris. The supernatant was filtered through a 0.22 μm filter to obtain the phage lysate. The titer of the phage lysate was determined using the double-layer agar plate method to determine the optimal MOI.

One-step Growth Curve Determination: A 1 mL aliquot of the logarithmic-phase target strain was mixed with 1 mL of phage suspension at the optimal MOI. The mixture was incubated (37°C, 200 rpm, 5 min) to allow phage adsorption. Subsequently, the mixture was centrifuged (5,000 × g, 1 min, 4°C) to pellet bacterial cells. The supernatant was discarded, and the pellet was washed twice with PBS to remove unadsorbed phages. The washed pellet was resuspended in 10 mL of BHI broth and incubated (37°C, 200 rpm). Samples were taken every 10 min to determine the phage titer and to draw the one-step growth curve of the phage infecting the target bacteria. Burst size was calculated as the ratio of the final viral titer at the plateau phase to the initial number of infected cells.

Host Range Determination: Multiple clinical isolates of *S. pseudintermedius* were cultured to the logarithmic growth phase, and each bacterial suspension was adjusted to a concentration of 1 × 10^8^ CFU/mL under sterile conditions. A 200 μL aliquot of the bacterial suspension was mixed with 3 mL of BHI medium (10 g/L agar), and then poured onto BHI agar plates (15 g/L agar in BHI broth), and left to stand for 30 min. Subsequently, 5 μL of the phage suspension was spotted onto the medium and left to stand for 10 min, followed by incubation at 37°C overnight. The presence or absence of clear zones was recorded.

Thermal Stability Determination: The thermal stability of the phage was evaluated by incubating phage suspensions in a constant-temperature water bath at different target temperatures. Briefly, 1 mL aliquots of the phage suspension were dispensed into sterile 1.5 mL centrifuge tubes. The tubes were incubated at different temperature gradients (30, 40, 50, 60, 70, and 80°C) for a total of 1 h. At 10-min intervals, 100 μL sample were collected from each tube and serially diluted 10-fold in sterile SM buffer. The phage titer of each diluted sample was determined using the double-layer agar plate method.

pH Stability Determination: Sterile SM buffer was adjusted to a series of pH values (3.0, 4.0, 5.0, 6.0, 7.0, 8.0, 9.0, 10.0, 11.0, 12.0, and 13.0). A 10 μL aliquot of the phage suspension was added to 990 μL of SM buffer with each pH value and the mixtures were incubated at room temperature for 1 h without shaking. After incubation, the phage titer of each sample was determined using the double-layer agar plate method.

### Whole-genome sequencing of the phage

Genomic DNA/RNA of the purified phages was extracted using the TaKaRa MiniBEST Viral RNA/DNA Extraction Kit Ver.5.0 (Takara, Japan) following the manufacturer’s instructions. The nucleic acid type of the phage genomes was determined by nuclease susceptibility assays: the extracted genetic material was treated with DNase I (Sigma-Aldrich, 2,000 U/mL, United States), RNase A (Sigma-Aldrich, 10 mg/mL, United States), and Mung Bean Nuclease (Sigma-Aldrich, 40 U/μL, United States), respectively, following the recommended reaction conditions. Subsequently, the treated and untreated genomic samples were subjected to agarose gel electrophoresis to verify integrity. Only samples with clear, non-degraded bands were selected for subsequent sequencing. Whole-genome sequencing was performed by Shanghai Personal Biotechnology Co., Ltd. (Shanghai, China) using a whole genome shotgun (WGS) strategy. Libraries with different insert sizes were constructed, and paired-end (PE) sequencing was carried out on the Illumina NovaSeq sequencing platform based on next-generation sequencing (NGS) technology.

### *In vitro* antibacterial activity assay of phages

Five clinical isolates of MRSP were selected and inoculated into 5 mL of BHI broth, followed by incubation at 37 ° with shaking at 180 rpm in a constant-temperature shaking incubator until reaching the logarithmic growth phase. The bacterial suspension was adjusted to a final concentration of 1 × 10^8^ CFU/mL. Meanwhile, phage suspensions were adjusted to four different concentrations: 1 × 10^6^ PFU/mL, 1 × 10^7^ PFU/mL, 1 × 10^8^ PFU/mL, and 1 × 10^9^ PFU/mL. The experiment was divided into three groups with a consistent total volume of 205 μL per well:

Positive control group: 200 μL of BHI broth + 5 μL of logarithmic-phase MRSP suspension; Negative control group: 205 μL of sterile BHI broth; Experimental groups: 100 μL of phage suspension (at each concentration) + 100 μL of BHI broth + 5 μL of logarithmic-phase MRSP suspension. After sample preparation, the initial OD_600_ value of each group was measured using a microplate reader. All samples were then incubated at 37°C, and OD_600_ values were measured every 1 h for a total of 12 h. Growth curves of MRSP were plotted based on the time-dependent OD_600_ values.

Subsequently, the MRSP strain showing the most significant bacteriostatis response to the phages (indicated by the largest reduction in OD_600_) was selected for combined antibacterial assays. Based on the results of susceptibility testing and clinical medication practices, the minimum inhibitory concentrations (MICs) of five antibiotics (oxacillin, ampicillin sodium, enrofloxacin, clindamycin, and doxycycline) against this MRSP strain were determined using the broth microdilution method, with *S. aureus* ATCC 29213 serving as quality control strain. For the combined assay, 1/2 MIC and 1/4 MIC of each antibiotic, along with phage suspensions at 1 × 10^7^ PFU/mL and 1 × 10^8^ PFU/mL, were used. The experiment was divided into 8 groups: 1 × 10^8^ PFU/mL phage group, 1/2 MIC antibiotic group, 1 × 10^8^ PFU/mL phage + 1/2 MIC antibiotic group, 1 × 10^8^ PFU/mL phage + 1/4 MIC antibiotic group, 1 × 10^7^ PFU/mL phage + 1/2 MIC antibiotic group, 1 × 10^7^ PFU/mL phage + 1/4 MIC antibiotic group, positive control group, and negative control group.

### Evaluation of the therapeutic efficacy of phages on a rabbit model of MRSP-induced pyoderma

The study was approved by the Animal Ethical and Welfare Committee of Northwest Agriculture and Forest University (Date of approval: March 11, 2022. Approval No: 20220102), and conducted in compliance with the ARRIVE Guidelines^1^ for the care and use of laboratory animals. Eight adult male New Zealand white rabbits (2.0∼2.5 kg) were housed under standardized conditions (temperature: 22 ± 2°C, humidity: 55 ± 5%, 12-h light/dark cycle) with free access to food and water. Administration of anesthesia and euthanasia were achieved by intravenous administration through the marginal ear vein.

Prior to modeling, the hair on the bilateral paravertebral dorsal skin regions (precisely localized 1.0–1.5 cm lateral to the dorsal midline) of each rabbit was shaved to expose an area of approximately 3 cm × 3 cm. Residual hair stubs were removed using a depilatory cream, which was retained on the skin surface for 3 min, then gently wiped off with sterile wet gauze and rinsed thoroughly with sterile normal saline. The skin was inspected to confirm the absence of pre-existing lesions or irritation. Four hours later, anesthesia was induced by intravenous injection of Telazol (1 mg/kg) and dexmedetomidine hydrochloride (2 μg/kg), and maintained using a facial mask connected to the respiratory anesthesia machine with 2% isoflurane (in 100% oxygen). Rabbits were placed in a prone position, and physiological parameters (heart rate, blood oxygen saturation, and non-invasive blood pressure) were continuously monitored throughout the procedure. Skin barrier disruption was performed using the tape stripping method: sterile adhesive tape was firmly applied to the depilated dorsal skin and rapidly peeled off, repeated 7–10 times with a new tape piece for each cycle (confirmed by mild erythema to indicate effective barrier disruption). Immediately after disruption, 100 μL of MRSP suspension (10^8^ CFU/mL) was uniformly smeared onto the damaged skin using a sterile pipette tip. After natural air-drying for 15 min, the inoculated area was covered with sterile gauze to maintain moisture and ensure bacterial adherence.

Each rabbit had two independent lesions (total of 16 lesions across 8 rabbits), which were randomly assigned to four groups (*n* = 4 lesions per group): the sole phage DW treatment group (Phage), the sole ampicillin sodium group (AMP), the combined phage DW-ampicillin sodium treatment group (Phage + AMP), and the control group (CON). All medications were administered through topical application. The phage titer was 10^8^ PFU/mL, with 100 μL administered each time; the dosage of ampicillin sodium was 12.5 mg/kg. Treatments were administered once every 24 h for a total of 120 h. After each administration, the lesion was gently rubbed for 30 s to ensure uniform coverage, then re-bandaged with sterile gauze.

Lesion scoring was initiated at 0 h (baseline) and repeated every 24 h for 120 h. The scoring system included four key indicators, each graded on a 4-point scale (0 = none, 1 = mild, 2 = moderate, 3 = severe): (1) erythema, (2) desquamation/crust formation, (3) skin irregularity (edema/erosion), and (4) skin induration (Borio et al., 2015). Scores were independently recorded by two blinded observers, and the average score was used for statistical analysis.

At 120 h post-treatment, all rabbits were humanely euthanized via intravenous injection of pentobarbital sodium (150 mg/kg, overdose). Each dorsal lesion was aseptically dissected to obtain a 2 cm × 2 cm full-thickness skin specimen, which was rinsed with sterile normal saline to remove residual blood and debris. Each specimen was divided into three subsamples: (1) bacterial load determination: a 0.5 cm × 0.5 cm subsample was weighed (mean weight: ∼0.1 g) and homogenized in 900 μL sterile normal saline using a tissue grinder. Serial 10-fold dilutions were prepared, and bacterial counts were determined using the standard plate count method on BHI agar plates, incubated at 37°C for 12 h. Results were expressed as log_10_ CFU/g tissue. (2) inflammatory factor assay: a 0.5 cm × 0.5 cm subsample was immediately snap-frozen in liquid nitrogen to preserve protein integrity and stored at −80°C until analysis. 20 mg of tissue was accurately weighed using an electronic balance and placed into a pre-chilled thick-walled centrifuge tube. Subsequently, 100 μL of pre-chilled PBS buffer containing protease inhibitor cocktail (1.05 mM AEBSF, 0.8 μM Aprotinin, 50 μM Bestatin, 15 μM E64, 20 μM Leupeptin, and 15 μM Pepstatin A) (Beyotime, China) was added, and the tissue was minced with autoclaved surgical scissors followed by thorough homogenization. The tissue homogenate was centrifuged at 10,000 × g for 10 min at 4°C using a pre-chilled high-speed refrigerated centrifuge. The resulting supernatant was collected for the determination of inflammatory cytokines (IL-1β, IL-6, and TNF-α), using commercial ELISA kits (Jiangsu Jingmei biotechnology, China) according to the manufacturer’s instructions. (3) histopathological examination: A 1 cm × 1 cm subsample was fixed in 4% paraformaldehyde in PBS (pH 7.4). After 24 h of fixation, samples were dehydrated through a graded ethanol series, embedded in paraffin, sectioned (5 μm thickness), and stained with hematoxylin and eosin for histological evaluation. Slides were examined by a veterinary pathologist blinded to group allocation.

### Phage proteomic analysis

Phage proteins were extracted from the phage suspension concentrated via PEG 8,000 precipitation: mixed with 5 × SDS loading buffer (4:1, v/v), boiled (95°C, 10 min), centrifuged (12,000 × g, 5 min, 25°C) to remove debris. 20 μL denatured protein was electrophoresed (12% separating gel, 5% stacking gel; 80 V, 30 min; 120 V, 90 min) with Tris-glycine buffer, stained with Coomassie Brilliant Blue R-250 (2 h), destained to confirm protein presence and purity. Extracted proteins (100 μL) were digested with trypsin (50:1, w/w, 37°C, overnight), desalted via C18 SPE column (Waters, United States), lyophilized, resuspended in 0.1% formic acid. A peptide spectral library was constructed via DDA on Q Exactive Plus (Thermo Fisher Scientific, United States). DIA was performed on the same spectrometer. Raw data were analyzed with Skyline (v21.1) against the library; protein annotation and functional classification via BLASTp (NCBI nr database), GO, KEGG enrichment.

### Statistical analysis

All experiments were performed in at least three biological replicates with three technical replicates per biological replicate. All data were expressed as the mean ± standard error, and analyzed using GraphPad Prism 8. Intergroup comparisons were conducted using unpaired *t*-tests (two groups) or one-way ANOVA with Tukey’s HSD *post-hoc* test (multiple groups). Intragroup comparisons were performed using paired *t*-tests or repeated measures one-way ANOVA with Bonferroni correction. A *P* < 0.05 was considered statistically significant.

## Results

### Epidemiological Investigation of canine pyoderma in Xi’an

A total of 103 canine pyoderma cases were included, consisting of 59 male dogs (57.28%) and 44 female dogs (42.72%). Among the 103 cases, dogs were grouped by age at 2-year intervals: 19 dogs (18.45%) under 2 years old, 26 dogs (25.24%) in the 2–3 years group, 18 dogs (17.48%) in the 4–5 years group, 22 dogs (21.36%) in the 6–7 years group, 7 dogs (6.8%) in the 8–9 years group, 6 dogs (5.83%) in the 10–11 years group, and 5 dogs (4.85%) over 11 years old (Supplementary Document 1).

The 103 affected dogs belonged to 24 breeds. The top 10 breeds with the highest incidence were as follows: Poodles (11.65%, *n* = 12), mixed breeds (10.68%, *n* = 11), Golden Retrievers (10.68%, *n* = 11), Border Collies (8.38%, *n* = 9), Shiba Inus (7.77%, *n* = 8), Bichons (7.77%, *n* = 8), Samoyeds (5.83%, *n* = 6), French Bulldogs (4.85%, *n* = 5), Akita Inus (4.85%, *n* = 5), and Welsh Corgis (4.85%, *n* = 5) (Supplementary Document 2).

The incidence of canine pyoderma was the highest in October (19, 18.45%), followed by September (18, 17.48%) and July (13, 12.62%). In contrast, the number of canine pyoderma cases was relatively low in January, February, March, and April, accounting for 0.97, 3.88, 3.88, and 2.91% of the total cases, respectively (Supplementary Document 3).

**FIGURE 1:**
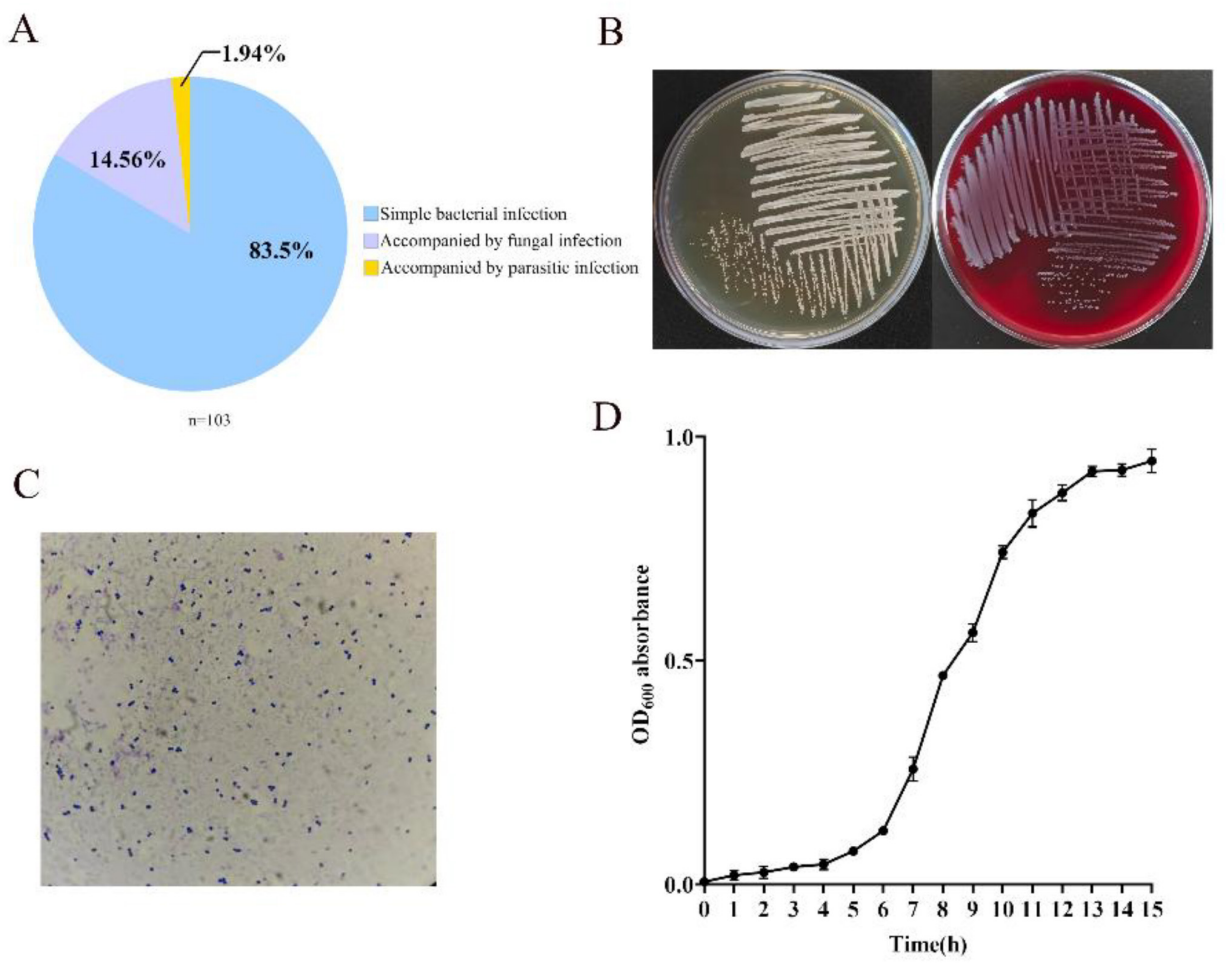
Clinical distribution of canine pyoderma and biological characteristics of *S. pseudintermedius*. **(A)** Etiological classification of pyoderma cases. **(B,C)** Colony morphology and Gram staining characteristics of isolated *S. pseudintermedius*. **(D)** Growth curve of *S. pseudintermedius*. All data were presented as means ± standard error, with error bars indicating standard error.

Based on microscopic examination of skin samples, canine pyoderma was primarily caused by uncomplicated bacterial infections, with a small proportion of cases accompanied by fungal or parasitic infections. Specifically, there were 86 cases of uncomplicated bacterial infections (83.5% of total cases), followed by 15 cases of fungal infections (14.56%), and 2 cases of parasitic infections (1.94%) (Figure 1A).

### Isolation, cultivation, and identification of pathogenic bacteria in canine pyoderma

A total of 82 bacterial strains were isolated from 42 canine pyoderma samples, with the 16S rDNA sequencing data provided in the Supplementary Document 4 and *Staphylococcus* (43.90%, 36/82) as the dominant genus. *S. pseudintermedius* accounted for 77.78% (28/36) of *Staphylococcus* isolates. Detailed pathogenic bacterial distribution is shown in Table 1. On Columbia blood agar medium and BHI agar medium, the isolated *S. pseudintermedius* formed circular, raised colonies with regular edges, smooth surfaces, and a grayish-white color; notably, β-hemolysis was observed on Columbia blood agar (Figure 1B). Microscopically, the bacteria were Gram-positive, arranged singly, in pairs, in short chains, or in irregular grape-like clusters (Figure 1C).

**TABLE 1:** Statistics of the pathogen distribution of canine pyoderma.

Bacterial genus	Bacteria	Strain number	Proportion (%)
Genus *Staphylococcus*	*Staphylococcus pseudintermedius*	28	34.14
	*Staphylococcus epidermidis*	1	1.22
	*Staphylococcus sciuri*	2	2.44
	*Staphylococcus saprophyticus*	1	1.22
	*Staphylococcus gallinarum*	1	1.22
	*Staphylococcus xanthus*	1	1.22
	*Staphylococcus haemolyticus*	1	1.22
	*Staphylococcus simulans*	1	1.22
Genus *Enterobacter*	*Escherichia coli*	15	18.29
Genus *Bacillus*	*Bacillus cereus*	5	6.10
	*Bacillus megaterium*	1	1.22
	*Bacillus glucanolyticus*	1	1.22
	*Bacillus licheniformis*	1	1.22
	*Bacillus safensis*	1	1.22
	*Bacillus pyocyaneus*	1	1.22
	*Bacillus thuringiensis*	1	1.22
Genus *Streptococcus*	*Streptococcus canis*	5	6.10
Genus *Acinetobacter*	*Acinetobacter haemolyticus*	1	1.23
	*Acinetobacter baumannii*	2	2.44
Genus *Enterococcus*	*Enterococcus faecalis*	2	2.44
Genus *Pseudomonas*	*Pseudomonas aeruginosa*	2	2.44
Genus *Pasteurella*	*Pasteurella*	1	1.22
Genus *Shigella*	*Shigella*	1	1.22
Genus *Proteus*	*Proteus*	1	1.22
Genus *Klebsiella*	*Klebsiella pneumoniae*	1	1.22
Genus *Campylobacter*	*Campylobacter jejuni*	1	1.22
Genus *Microbacterium*	*Microbacterium*	1	1.22
Genus *Aerococcus*	*Aerococcus urinae*	1	1.22
Genus *Actinomyces*	*Actinomycetes*	1	1.22

### Drug susceptibility test and growth curve of canine-derived *S. pseudintermedius*

Based on clinical medication practices, 11 antibiotics were selected to determine the drug susceptibility of 28 clinically isolated canine-derived *S. pseudintermedius*. β-lactam antibiotics: the resistance rate of oxacillin was 21.43%, while resistance rates to penicillin G and ampicillin sodium were as high as 96.43 and 92.85%, respectively; the resistance rate to amoxicillin-clavulanate potassium was relatively low at 7.14%. The resistance rates to aminoglycoside, sulfonamide, and lincosamide antibiotics all exceeded 50%. The resistance rates to quinolone, tetracycline, and chloramphenicol antibiotics were relatively lower, ranging from 10 to 30%. *S. pseudintermedius* showed the highest sensitivity to rifamycin drugs, with a resistance rate of only 3.57% (Table 2).

**TABLE 2:** Results of drug sensitivity test for *S. pseudintermedius.*

Drug types	Medication	Diameter			Sensitive situations			Drug resistance rate
		S( ≥ )	I	R( ≤ )	Resistant	Intermediate	Sensitive	
β-lactam antibiotics	Oxacillin	18	–	17	6	–	22	21.43%
	Penicillin G	29	–	28	27	–	1	96.43%
	Ampicillin sodium	17	–	16	26	–	2	92.85%
	Amoxicillin/clavulanic acid potassium	18	14∼17	13	2	2	24	7.14%
Aminoglycosides	Gentamicin	15	13∼14	12	14	4	9	50%
Quinolones	Enrofloxacin	23	17∼22	16	7	8	13	25%
Sulfonamides	Compound sulfamethoxazole	16	11∼15	10	19	0	9	67.86%
Lincomycin derivatives	Clindamycin	21	15∼20	14	17	1	10	60.71%
Tetracyclines	Doxycycline	16	13∼15	12	6	5	17	21.43%
Rifamycin derivatives	Rifampicin	20	17∼19	16	1	0	27	3.57%
Chloramphenicols	Florfenicol	18	13∼17	16	4	1	23	14.28%

Continuous determination of OD_600_ over a 15-h incubation period revealed that *S. pseudintermedius* exhibited a Lag phase during the initial culture stage (1–5 h), transitioned to the logarithmic growth phase at 6 h, and entered the stationary phase from 13 h onwards (Figure 1D).

### Isolation and characterization of canine-derived *S. pseudintermedius* phages

**FIGURE 2:**
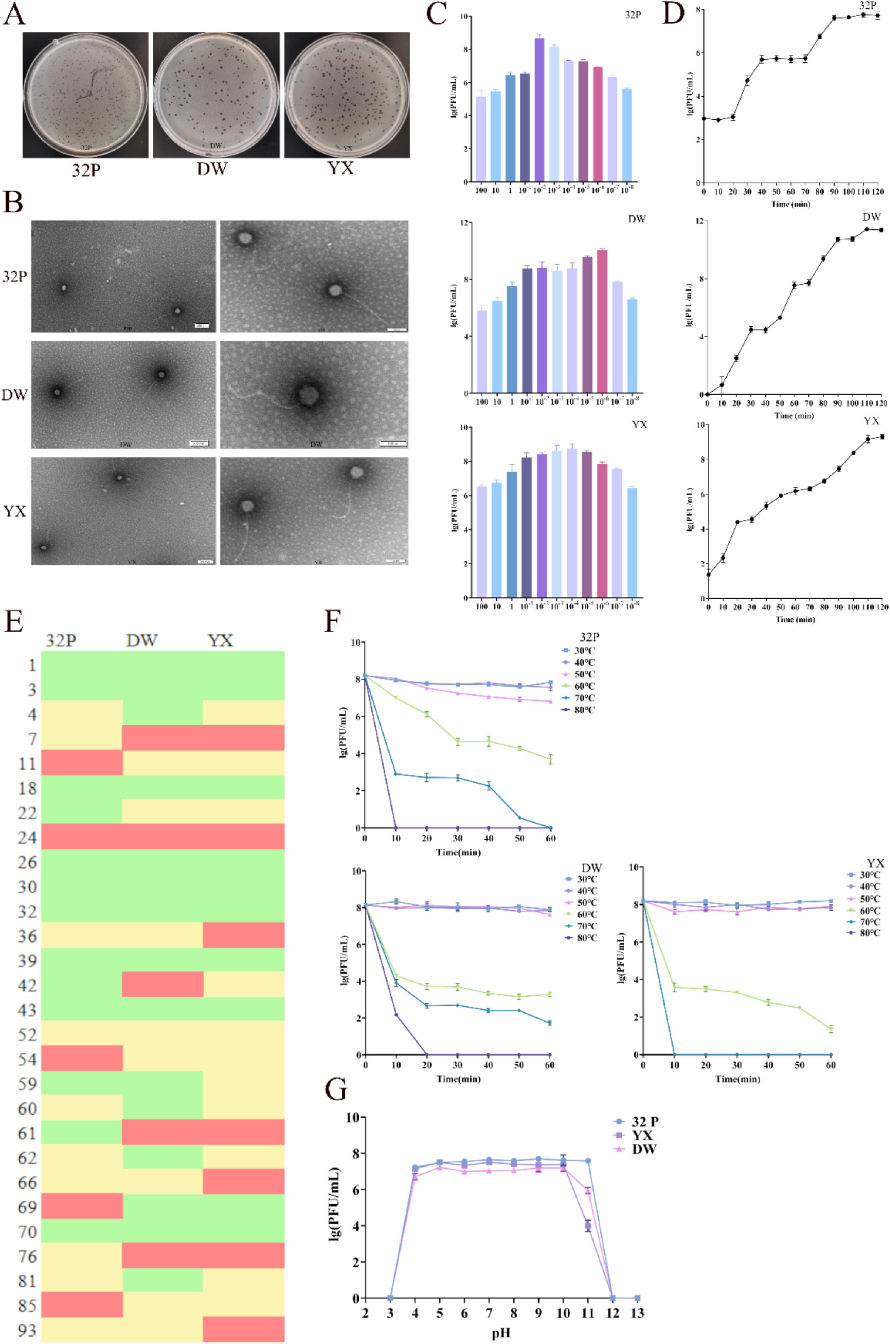
Characteristics of *S. pseudintermedius* phages. **(A)** Plaque morphology and diameter of phages. **(B)** Morphological characteristics of phages visualized by transmission electron microscopy. **(C)** Optimal MOI of phages 32P, DW, and YX. **(D)** One-step growth curves of phages 32P, DW, and YX. Burst size was calculated as the ratio of the final viral titer at the plateau phase to the initial number of infected cells. **(E)** Lysis profiles of phages 32P, DW, and YX against 28 *S. pseudintermedius* strains. Each strain is represented by Arabic numerals 1–93. Green indicates that the phage can completely lyse the bacterium, forming transparent plaques; yellow indicates incomplete lysis, forming opaque plaques; and red indicates that the phage cannot lyse the bacterium, with no plaques observed. **(F)** Temperature stability of the phages. **(G)** pH stability of the phages. All data were presented as means ± standard error, with error bars indicating standard error.

A total of three phages were isolated in this study and designated as 32P, DW, and YX. The purified phages formed clear, transparent plaques with distinct edges (Figure 2A).

### The biological characteristics of canine-derived *S. pseudintermedius* phages

Transmission electron microscopy indicated that phages 32P, DW, and YX shared an icosahedral head (diameter: 85 ± 5 nm, *n* = 12) and long tail (length: 350 ± 5 nm, *n* = 12) (Figure 2B). The optimal MOI was 10^–2^, 10^–6^, and 10^–4^ for 32P, DW, and YX, respectively, with corresponding maximum titers of 5.76 × 10^8^ PFU/mL, 1.21 × 10^10^ PFU/mL, and 2.22 × 10^9^ PFU/mL (Figure 2C).

One-step growth curve assays were performed to characterize the proliferative kinetics of phages 32P, DW, and YX (Figure 2D). For phage 32P, the latent period was determined to be 20 min; the lysis cycle lasted approximately 20 min, and the burst size was calculated as 216 PFU/cell. In contrast, phage DW exhibited a shorter latent period of 10 min, with a lysis cycle duration of ∼20 min and a higher burst size of 299 PFU/cell. For phage YX, the latent period was also 10 min, but its lysis cycle was markedly prolonged (lasting ∼100 min), and the corresponding burst size was 190 PFU/cell.

Against 28 clinically *S. pseudintermedius* isolates, the lysis rates of 32P, DW, and YX were 82.14, 82.14, and 75.00%, respectively, with DW exhibiting the highest complete lysis rate (transparent plaques, 53.57%) (Figure 2E). Thermal stability assays demonstrated that all three phages maintained stable titers at 30–50°C for 1 h. Phage 32P was inactivated after 60 min at 70°C or 10 min at 80°C; DW remained active after 1 h at 70°C but was inactivated after 20 min at 80°C; YX was inactivated after 10 min at 70°C or 80°C (Figure 2F). All phages retained stable titers at pH 4–10, were completely inactivated at pH 3 and pH 12, and DW and YX showed reduced titers at pH 11 (Figure 2G).

### Whole-genome sequencing of the phage

**FIGURE 3:**
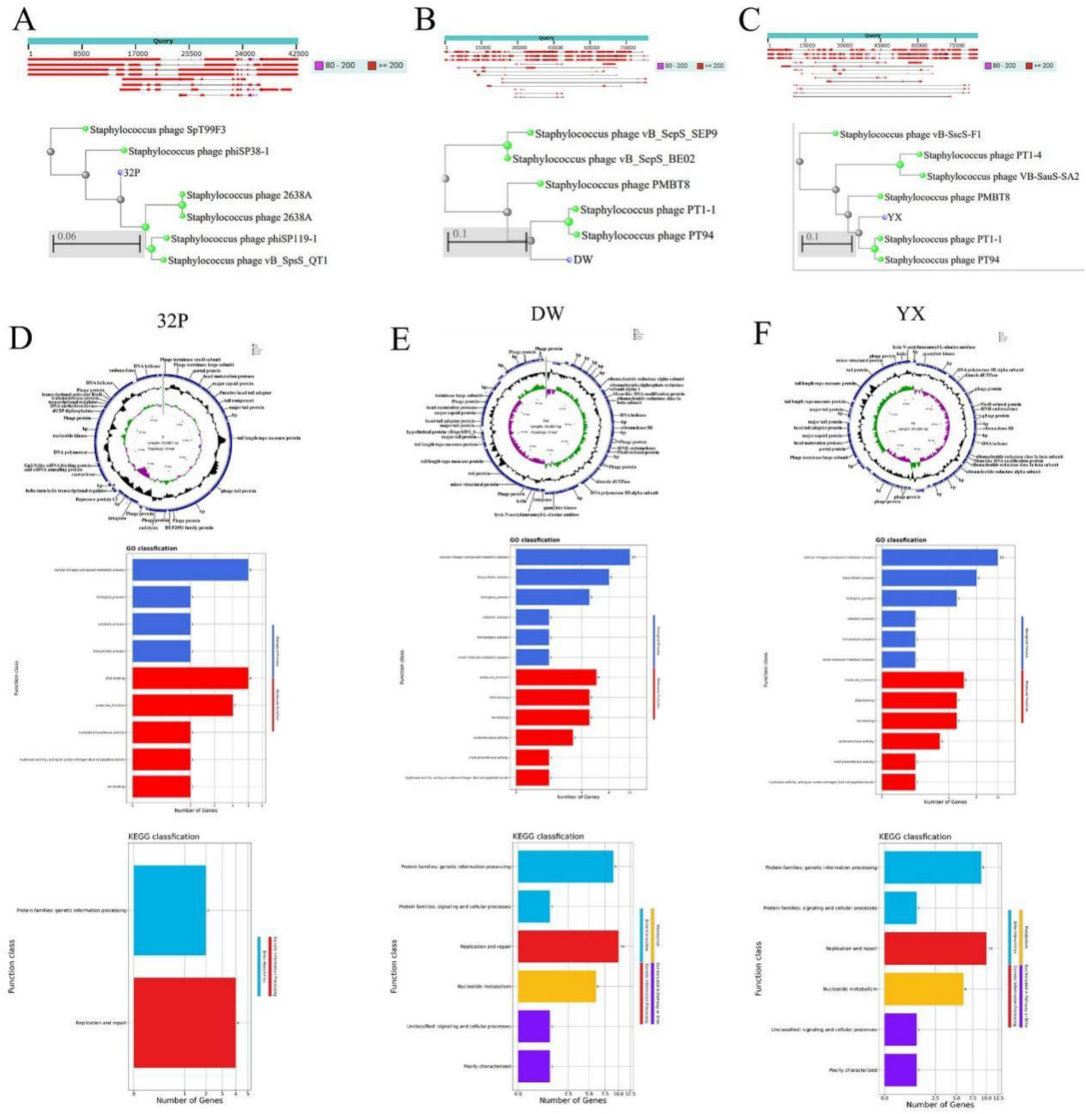
Phage genome sequencing. **(A–C)** Taxonomic classification of the three phages. **(D–F)** Genomic circular maps and GO functional annotation diagrams of phages 32P, DW, and YX.

All three phages were identified as double-stranded DNA (dsDNA) viruses (nucleic acids of all three phages were completely degraded by DNase I, yet remained insensitive to both RNase A and Mung Bean Nuclease), classified into the phylum *Duplodnaviria*, class *Heunggongvirae*, phylum *Uroviricota*, order *Caudoviricetes*, and belong to *Staphylococcus* phages (Figures 3A–C). Phages 32P and DW harbored linear dsDNA, while YX contained circular dsDNA (Table 3). All three phages exhibited an open reading frames (ORFs) density of 1.3–1.5 genes/kb, with ORFs accounting for > 85% of the total genome and an average ORF length of 600–700 bp.

**TABLE 3:** Genomic characteristics of the three phage strains.

The phage name	P32	DW	YX
Genome size (bp)	42,667	84,084	84,084
GC content (%)	36.84	32.02	32.03
DNA type	dsDNA(linear)	dsDNA (linear)	DsDNA (circular)
ORF num	58	120	121
ORF total length (bp)	40,380	73,233	73,434
ORF density (genes/kb)	1.359	1.427	1.439
Longest ORF length (bp)	6,069	5,448	5,448
ORF average length (bp)	696.21	606.89	610.27
ORF/Genome (coding percentage) (%)	94.64	87.10	87.33
GC content in ORF region (%)	36.92	32.34	32.38
Hypothetical proteins	22	81	85
Functional proteins	36	39	36

Phage 32P had 58 ORFs (36 structural/functional proteins, 22 putative proteins) (Figure 3D; Supplementary Document 5). Key modules included structural proteins (portal protein, major capsid protein, major tail protein), DNA replication/metabolism proteins (integrase, repressor protein CI, DNA polymerase, helicase), DNA packaging proteins (terminase small/large subunits), and lysis proteins (holin and endolysin). GO enrichment focused on catabolic processes and DNA binding; KEGG enrichment centered on replication and repair (Figure 3D).

Phage DW had 120 ORFs (39 structural/functional proteins, 81 putative proteins) (Figure 3E; Supplementary Document 6). Core modules included structural proteins (major capsid protein, major tail protein), DNA replication/metabolism proteins (ribonucleoside-diphosphate reductase, Mom-like DNA modification protein), DNA packaging protein (terminase large subunit), and lysis proteins (holin and N-acetylmuramoyl-L-alanine amidase). GO enrichment involved catabolic processes, DNA binding, and ion binding; KEGG enrichment included replication and repair, and nucleotide metabolism (Figure 3E).

Phage YX had 121 ORFs (36 structural/functional proteins, 85 putative proteins) (Figure 3F; Supplementary Document 7). Its main protein modules were consistent with DW, except for the absence of integrase. GO enrichment focused on catabolic processes, DNA binding, and ion binding; KEGG enrichment centered on replication and repair, and nucleotide metabolism (Figure 3F).

### *In vitro* antibacterial activity of phage DW

**FIGURE 4:**
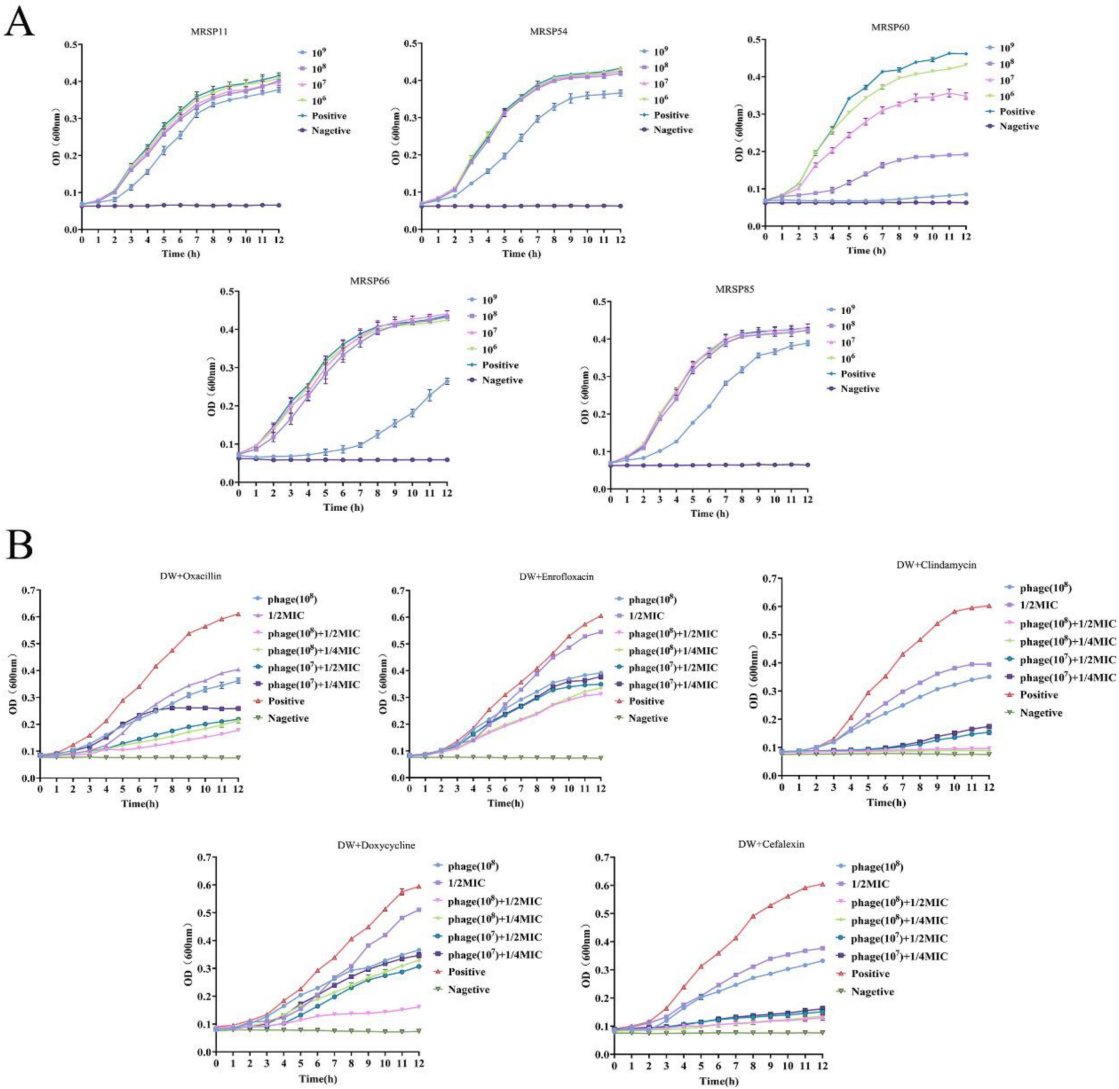
*In vitro* antibacterial activity of phage DW against MRSP. **(A)** *In vitro* antibacterial effects of phage DW (at different titers) against 5 MRSP strains (11, 54, 60, 66, 85). The optimal antibacterial activity was observed at a phage titer of 10^9^ PFU/mL, with the most titer-dependent variation in efficacy against MRSP strain 60. **(B)** Synergistic antibacterial effects of phage DW (at different titers) combined with antibiotics (1/2 or 1/4 MIC). The combination therapy exhibited superior efficacy compared to phage monotherapy or antibiotic monotherapy, with phage DW enhancing MRSP sensitivity to antibiotics and reducing antibiotic dosage. All data were presented as means ± standard error, with error bars indicating standard error.

Given its highest complete lysis (transparent plaques) rate against 28 *S. pseudintermedius* isolates, phage DW was selected for subsequent *in vitro* antibacterial assays. No significant differences in antibacterial efficacy were observed among different phage titers during the early culture phase (<2 h, *P* > 0.05). In the late culture stage, the optimal antibacterial effect was achieved at a phage titer of 10^9^ PFU/mL, with notably enhanced reduction in OD_600_ absorbance against MRSP strains 54, 60, 66, and 85. Among the five tested MRSP strains, strain 60 exhibited the most significant titer-dependent reduction to phage DW (Figure 4A).

The MIC of antibiotics against MRSP strain 60 were determined as follows: ampicillin sodium (256 μg/mL), oxacillin (8 μg/mL), clindamycin (256 μg/mL), enrofloxacin (16 μg/mL), and doxycycline hydrochloride (256 μg/mL). Combinatorial assays of phage DW (various titers) with antibiotics (1/2 or 1/4 MIC) demonstrated superior *in vitro* antibacterial efficacy compared to either phage or antibiotics alone (Figure 4B). When the phage titer was fixed, no significant differences in antibacterial activity were observed between the two antibiotic concentrations. In contrast, at a fixed antibiotic concentration, higher phage titers correlated with enhanced antibacterial effects against MRSP.

### Therapeutic efficacy of phage DW in a rabbit model of *S. pseudintermedius*-induced pyoderma

**FIGURE 5:**
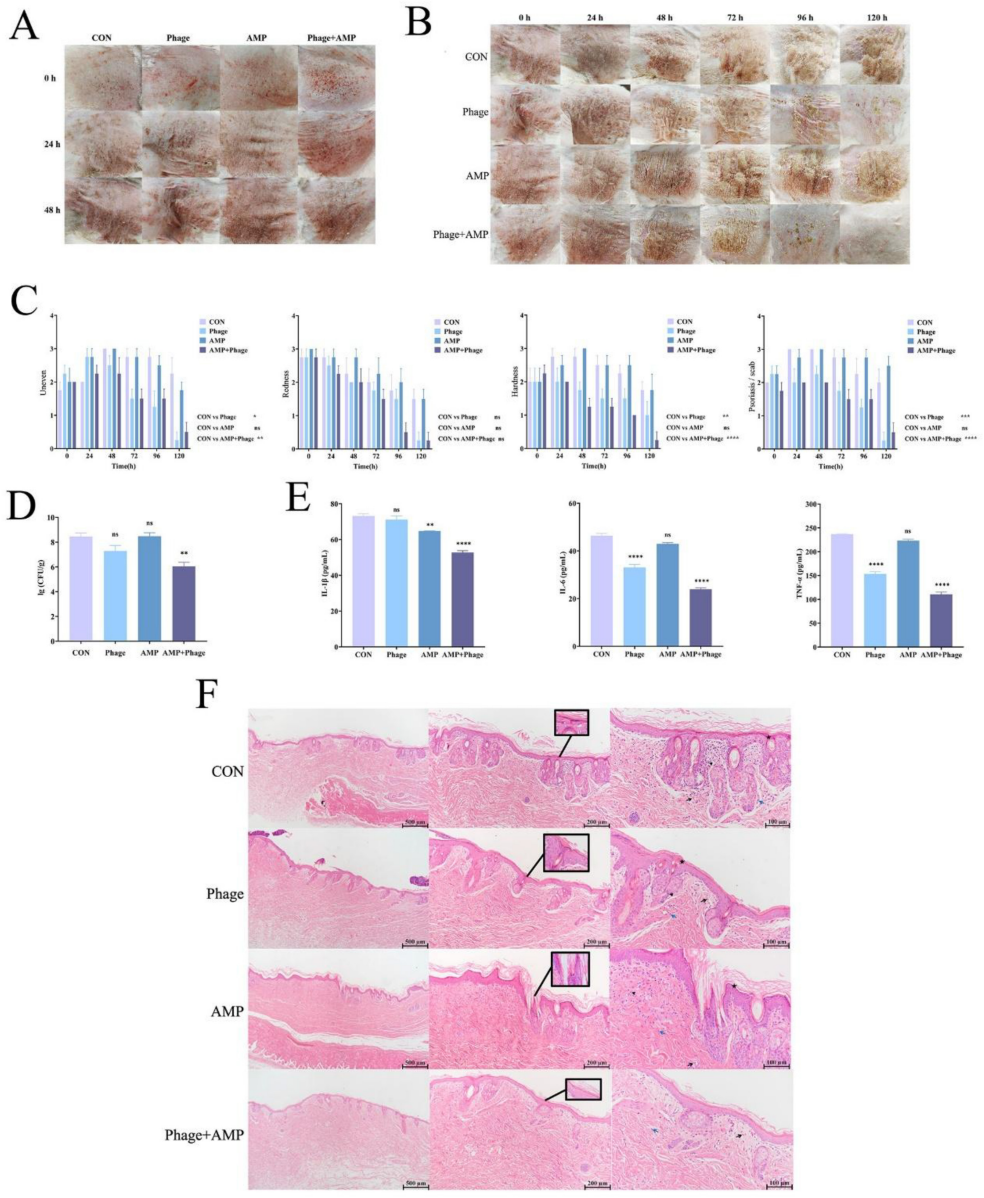
Therapeutic effects of phage-based therapy on the *S. pseudintermedius*-induced pyoderma. **(A)** Skin lesions of rabbits within 48 h post-model establishment, characterized by dandruff, crusting, skin thickening, and purulent exudation. **(B)** Gross skin appearance of each group after 120 h of treatment. **(C)** Dynamic changes in skin lesion scores during treatment. **(D)** Skin tissue bacterial load of each group. **(E)** Expression levels of inflammatory factors in skin. **(F)** Histopathological changes of skin tissue. All data were presented as means ± standard error, with error bars indicating standard error. * indicates *P* < 0.05, and ** indicates *P* < 0.01.

Within 48 hours after model establishment, the rabbit skin symptoms gradually exacerbated, and manifested as dandruff, crusting, skin thickening, and purulent exudate in some rabbits (Figure 5A).

After 120 h of treatment, the CON group and AMP group still exhibited extensive crusting, dandruff, skin thickening, increased hardness, and bleeding upon crust removal. In contrast, the phage group showed mild crusting, dandruff, and erythema, with reduced severity and softer skin compared to the CON group and AMP group, while the phage + AMP group presented nearly normal skin—AMP gr texture, no obvious erythema or crusting, and only minimal dandruff (Figure 5B).

Skin injury scores revealed similar injury degrees among all groups from 0 to 24 h (Figure 5C). The phage group showed reduced score relative to CON (*P* < 0.05), with the exception of the redness-associated score. AMP monotherapy showed limited therapeutic potential, as no significant differences in scores were observed between the AMP and CON groups (*P* > 0.05). The AMP + phage group also exhibited reduced score relative to CON (*P* < 0.01), with the exception of the redness-associated score.

There was no significant difference in skin bacterial load among the CON, phage, and AMP groups (*P* > 0.05). The AMP + phage group exhibited a marked reduction in skin bacterial load compared to the CON group (*P* < 0.01) (Figure 5D).

IL-6 and TNF-α levels were reduced in the phage group and AMP + phage group compared to the CON group (*P* < 0.01), with no difference between the CON and AMP groups (*P* > 0.05), and the levels in the AMP + phage group were lower than those in the phage group (*P* < 0.05). For IL-1β, no significant difference was observed between the CON and phage groups (*P* > 0.05), while the AMP group showed significant downregulation (*P* < 0.01 vs. CON) and the phage + AMP group exhibited a more pronounced decrease (*P* < 0.01 vs. CON) (Figure 5E).

Histopathological observations showed that the CON and AMP groups had marked epidermal thickening, indistinct epidermis-dermis boundaries, massive bacterial aggregates in the epidermis and hair follicle tips, severe neutrophil infiltration, fibroblast proliferation, and hair follicle abnormalities—with the AMP group additionally showing increased extravascular red blood cells. The phage group was superior to the CON and AMP groups, with a clear epidermis-dermis boundary, milder epidermal thickening, reduced bacterial density in aggregates, and moderate inflammatory cell infiltration/fibroblast proliferation. The phage + AMP group exhibited complete tissue recovery, with distinct layering of the epidermis, dermis, and subcutaneous tissue, a smooth epidermal surface, loose stratum corneum with minimal psoriasis/scab, no obvious hair follicle abnormalities, only slight bacterial aggregation on the epidermal surface, and no significant inflammatory infiltration or fibroblast proliferation (Figure 5F).

### Proteomic profiling of phage DW and 32P

**FIGURE 6:**
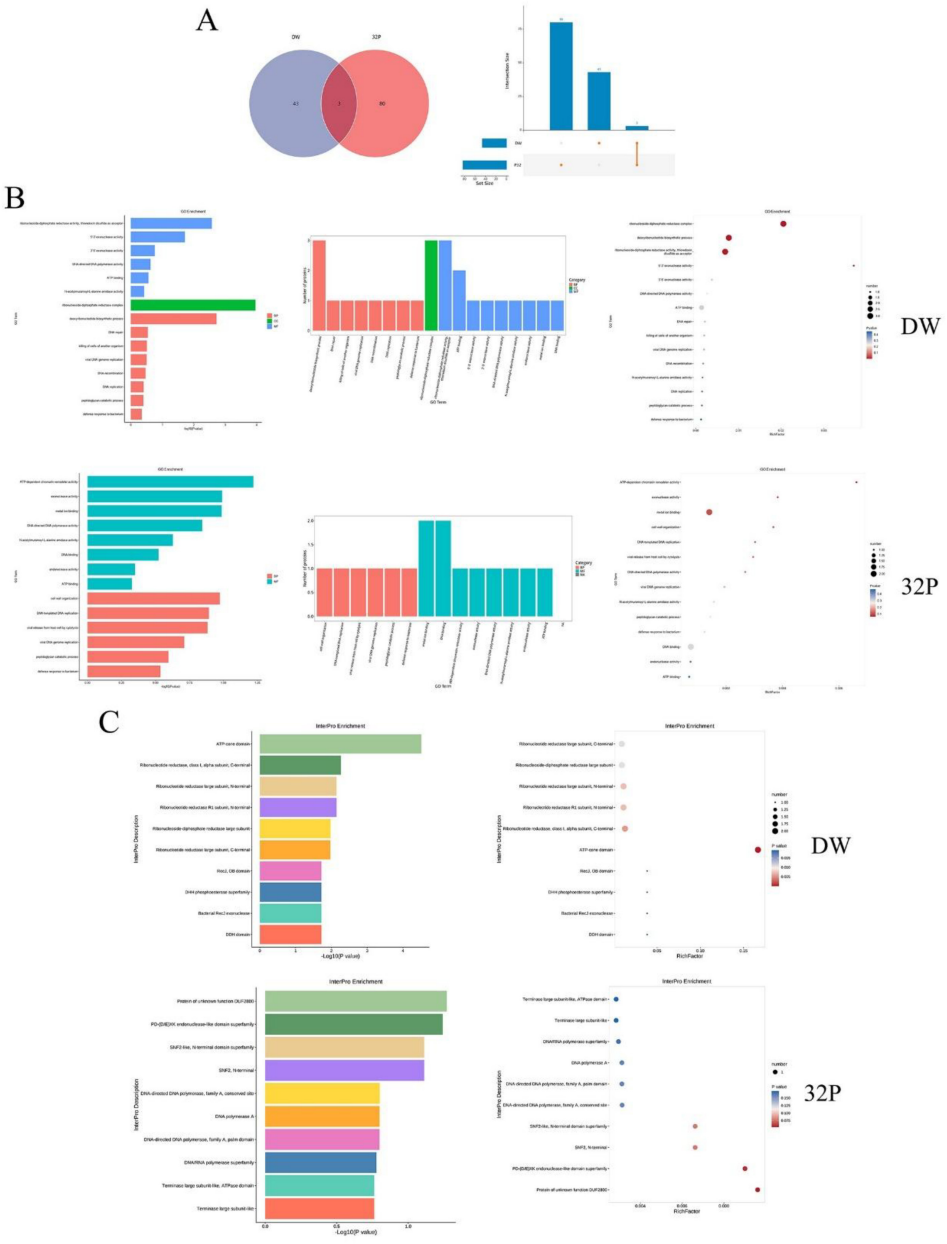
Proteomic analysis of *S. pseudintermedius* phages 32P and DW. **(A)** Number of identified proteins in phages DW (46) and 32P (83), with 3 common proteins shared between the two. **(B)** Functional enrichment of phage proteins. **(C)** Key protein domains of phages.

A total of 46 proteins were identified in phage DW, and 83 proteins in phage 32P, with 3 common proteins shared between the two phages (Figure 6A; Supplementary Documents 8, 9). GO functional annotation of the significantly enriched proteins was performed to clarify their functional categories and characteristics. For phage DW, numerous proteins were enriched in functions including DNA-directed DNA polymerase activity, ribonucleoside-diphosphate reductase complex, killing of cells of another organism, viral DNA genome replication, DNA replication, and peptidoglycan catabolic process (Figure 6B). In phage 32P, a large number of proteins were enriched in functions such as DNA-directed DNA polymerase activity, DNA-templated DNA replication, viral release from host cells by cytolysis, viral DNA genome replication, peptidoglycan catabolic process—lic of which played crucial roles in the lysis of *S. pseudintermedius* by the phages. Additionally, protein domain analysis revealed distinct domain profiles: DW contained domains including Ribonucleotide reductase (class I, alpha subunit, C-terminal), Ribonucleotide reductase large subunit (N-terminal), Ribonucleotide reductase R1 subunit (N-terminal), Ribonucleoside-diphosphate reductase large subunit, RecJ, OB domain, DHH phosphoesterase superfamily, Bacterial RecJ exonuclease, and DDH domain; while the main domains in 32P were Terminase large subunit-like, ATPase domain, DNA/RNA polymerase superfamily, DNA-directed DNA polymerase (family A, conserved site), SNF2-like N-terminal domain superfamily, and Protein of unknown function (DUF2800) (Figure 6C).

## Discussion

Canine pyoderma is a prevalent bacterial skin infection in dogs. Currently, antibiotic therapy remains the primary treatment, but irrational use of antibiotics in veterinary practice has driven the emergence and spread of antibiotic-resistant strains—posing significant challenges to clinical management. Additionally, cross-infection between animals and even between animals and humans threatens public health. The identification of its pathogen has evolved over time: initially, *S. aureus* was considered the primary causative agent. With technological advancements, *S. intermedius* was first isolated by Hajek in 1976, thereby replacing *S. aureus* as the main pathogen associated with canine pyoderma (Petridou et al., 2010). In 2005, *S. pseudintermedius* was identified, exhibiting distinct growth characteristics and biochemical properties compared to *S. intermedius* (Kizerwetter-Swida et al., 2018). Domestic and international studies consistently indicate that *S. pseudintermedius* is the primary pathogen underlying canine pyoderma and surgical site infections (Dégi et al., 2024; Hnilica and May, 2004). In this study, 28 *S. pseudintermedius* strains were isolated from 42 canine pyoderma samples. Consistent with previous findings, these results confirm that *S. pseudintermedius* is the main pathogen of canine pyoderma in Xi’an region, China.

In recent years, the isolation rates of MRSP and multi-drug resistant *S. pseudintermedius* have been increasing globally (Bergström et al., 2012; Hanselman et al., 2008; Kizerwetter-Świda et al., 2019). MRSP poses a significant threat to animal health. Due to its inherent multidrug resistance, it greatly complicates antibacterial treatment, posing a major challenge to the clinical management of canine pyoderma. Companion animals play a pivotal role in the MRSP epidemiology: their close contact with humans facilitates *Staphylococcus* transmission to humans, leading some scholars to hypothesize that these animals may act as reservoirs for drug-resistant *Staphylococcus* (Kmieciak and Szewczyk, 2018). Recent studies have confirmed that humans can be infected with animal-derived *S. pseudintermedius* (Ghosh et al., 2018). *S. pseudintermedius* poses a notable threat to human health, highlighting the need for increased attention to antimicrobial resistance in companion animal-derived bacteria. In this study, 11 antibiotics were selected to assess the drug susceptibility of clinical *S. pseudintermedius* isolates from dogs. Resistance rates were exceptionally high for penicillin G (96.43%), and ampicillin sodium (92.85%), while rates exceeding 50% were observed for aminoglycosides, sulfonamides, and lincosamides. These findings confirm that the *S. pseudintermedius* isolates exhibit multiple resistance, complicating the clinical management of canine pyoderma. Additionally, this raises public health concerns and threatens the treatment of human *S. pseudintermedius* infections.

Significant progress has been achieved globally in using phages and their products to treat superbug-induced infections, highlighting their great potential for managing multidrug-resistant bacterial diseases (Ghosh et al., 2018; Iszatt et al., 2021; Mboowa, 2023). In the phage-based treatment of bacterial infections, the emergence of resistant bacterial strains triggers continuous phage evolution to adapt to host bacterial variations. This drives phage-host co-evolution, thereby minimizing the likelihood of bacterial escape (Santamaría-Corral et al., 2024; Umansky and Fortier, 2023). In this study, three lytic phages (32P, DW, and YX) were isolated. Their lysis rates (transparent plaques and opaque plaques) against 28 *S. pseudintermedius* strains were 82.14, 82.14, and 75%, respectively, with DW exhibiting the highest complete lysis rate (transparent plaques, 53.57%). These findings lay a foundation for developing phage-based formulations, while further investigations into their *in vivo* safety, and synergistic mechanisms with antibiotics will enhance their translational potential in veterinary clinical practice.

Before proliferating within bacterial hosts, phages must first evade the defense mechanisms of their hosts (Shi et al., 2022). Phages are capable of inhibiting biofilm formation or disrupting performed biofilms via enzymes like endolysins (Wang et al., 2023). Following injection of the phage genome, it can utilize the host bacterium’s methyltransferase for genomic modification, thereby evading host restriction endonuclease-mediated cleavage through mechanisms such as REase avoidance and direct inhibition of REase activity (Nayak et al., 2025; Rózsa et al., 2025). Phages can inhibit the toxin factors of host bacteria by generating antitoxins or mimicking bacterial regulatory factors, thereby blocking abortive infection systems and safeguarding their progeny (Blower et al., 2012; Kelly et al., 2023; Naka et al., 2014; Wan et al., 2016). In this study, all three phages were identified as *Staphylococcus* phages. Via gene and protein sequencing, it was discovered that their related genes and encoded proteins—including DNA replication-related proteins, holin, lysin N-acetylmuramoyl-L-alanine amidase, endolysin, and proteins enriched in interspecific cell killing, viral DNA genome replication, and viral release from host cells via cytolysis—play significant roles in the phage-mediated lysis of *S. pseudintermedius*.

Numerous studies have confirmed that phage-antibiotic combinations can retard the emergence of bacterial drug resistance while improving therapeutic efficacy (Devi, 2024; Fungo et al., 2023; North and Brown, 2021). Phages can enhance bacterial susceptibility to antibiotics and reduce the MIC (Gu Liu et al., 2020). Under phage-antibiotic synergy, certain antibiotics can trigger the release of additional phage particles from host bacteria or induce phage release at sub-lethal concentrations, thereby reducing the antibiotic dosage required for treatment. Furthermore, phages can synthesize various enzymes—including endolysins and lyases—that degrade bacterial biofilms, facilitating antibiotic diffusion and bacterial eradication. In this study, when phage DW at different titers was combined with antibiotics at 1/2 or 1/4 MIC, the *in vitro* antibacterial efficacy was superior to that of phage or antibiotic monotherapy. Phage DW enhanced the susceptibility of MRSP to antibiotics, reduced antibiotic usage, and thereby alleviated the selective pressure driving bacterial drug resistance. Additionally, the therapeutic efficacy of phage DW against pyoderma was validated using an animal disease model. The AMP + phage group exhibited the best therapeutic effect, followed by the phage group—further confirming the validity of phage-antibiotic combination therapy.

However, this study has several limitations that should be acknowledged. First, while the research focuses on canine pyoderma, rabbits were employed as the animal model to evaluate therapeutic efficacy instead of dogs. This choice was primarily justified by ethical and practical considerations. Interspecies differences in skin structure, immune response, and bacterial-host interactions may limit the direct translational relevance of the *in vivo* findings to canine clinical practice. Second, the sample size of animals used in the therapeutic evaluation was relatively small (n = 8 per group). Additionally, long-term follow-up data on the recurrence of pyoderma and potential development of phage resistance in clinical settings were not collected, which should be addressed in future investigations to fully assess the clinical applicability of the proposed phage-based strategy.

## Conclusion

Three high-efficiency lytic phages were isolated and characterized using MRSP strains from canine pyoderma cases. These phages enhanced MRSP susceptibility to antibiotics and reduced antibiotic dosage, with both phage monotherapy and AMP-phage combination therapy achieving the favorable therapeutic outcomes for pyoderma. This study provides a novel therapeutic strategy for veterinary clinical management of MRSP-associated canine pyoderma and valuable insights for mitigating MRSP infections to safeguard public health.

## Data Availability

The datasets presented in this study can be found in online repositories. The names of the repository/repositories and accession number(s) can be found in the article/Supplementary material.

## Footnotes

1
https://arriveguidelines.org
