# Supplementary material for: Phage therapy against methicillin-resistant Staphylococcus pseudintermedius: a novel strategy for canine pyoderma
Source: Front Microbiol. 2026 Jan 13;16:1719973. doi: 10.3389/fmicb.2025.1719973 (PMC12835223; doi:10.3389/fmicb.2025.1719973)
Supplement: Supplementary file 1 [file Table_1.docx]

The distribution of pyoderma in canine of different ages

| Age (years) | <2 | | 2-3 | | 4-5 | | 6-7 | | 8-9 | | 10-11 | | >11 |  |
| --- | --- | --- | --- | --- | --- | --- | --- | --- | --- | --- | --- | --- | --- | --- |
|  | 0 | 1 | 2 | 3 | 4 | 5 | 6 | 7 | 8 | 9 | 10 | 11 | >11 |  |
| Number of cases (cases) | 6 | 13 | 7 | 19 | 10 | 8 | 15 | 7 | 4 | 3 | 5 | 1 | 5 | |
| Total number (cases) | 19 | | 26 | | 18 | | 22 | | 7 | | 6 | | 5 |  |
| Percentage (%) | 18.45 | | 25.24 | | 17.48 | | 21.36 | | 6.80 | | 5.83 | | 4.85 | |
