# Supplementary material for: Phage therapy against methicillin-resistant Staphylococcus pseudintermedius: a novel strategy for canine pyoderma
Source: Front Microbiol. 2026 Jan 13;16:1719973. doi: 10.3389/fmicb.2025.1719973 (PMC12835223; doi:10.3389/fmicb.2025.1719973)
Supplement: Supplementary file 2 [file Table_2.docx]

The percentage of pyoderma in different breeds of canine

| Breed | Number of cases (cases) | Percentage (%) | Breed | Number of cases (cases) | Percentage (%) |
| --- | --- | --- | --- | --- | --- |
| Poodle | 12 | 11.65 | Mixed-breed dog | 11 | 10.68 |
| Golden retriever | 11 | 10.68 | Border collie | 9 | 8.38 |
| Shiba Inu | 8 | 7.77 | Bichon | 8 | 7.77 |
| Samoyed | 6 | 5.83 | French bulldog | 5 | 4.85 |
| Akita Inu | 5 | 4.85 | Welsh corgi | 5 | 4.85 |
| Labrador retriever | 3 | 2.91 | Shih Tzu | 3 | 2.91 |
| Alaska malamute | 2 | 1.94 | Yorkshire | 2 | 1.94 |
| Pomeranian | 2 | 1.94 | Beagle | 2 | 1.94 |
| Maltese | 2 | 1.94 | English bulldog | 1 | 0.97 |
| Schnauzer | 1 | 0.97 | Pug | 1 | 0.97 |
| Bristle fox terrier | 1 | 0.97 | Pit bull | 1 | 0.97 |
| Chihuahua | 1 | 0.97 | German shepherd dog | 1 | 0.97 |
