# Supplementary material for: Phage therapy against methicillin-resistant Staphylococcus pseudintermedius: a novel strategy for canine pyoderma
Source: Front Microbiol. 2026 Jan 13;16:1719973. doi: 10.3389/fmicb.2025.1719973 (PMC12835223; doi:10.3389/fmicb.2025.1719973)
Supplement: Supplementary file 3 [file Table_3.docx]

The percentage of canine pyoderma in different months

| Month | 1 | 2 | 3 | 4 | 5 | 6 | 7 | 8 | 9 | 10 | 11 | 12 |
| --- | --- | --- | --- | --- | --- | --- | --- | --- | --- | --- | --- | --- |
| Number of cases (cases) | 1 | 4 | 4 | 3 | 7 | 9 | 13 | 8 | 18 | 19 | 10 | 7 |
| Percentage (%) | 0.97 | 3.88 | 3.88 | 2.91 | 6.80 | 8.74 | 12.62 | 7.77 | 17.48 | 18.45 | 9.71 | 6.80 |
