# Supplementary material for: Phage therapy against methicillin-resistant Staphylococcus pseudintermedius: a novel strategy for canine pyoderma
Source: Front Microbiol. 2026 Jan 13;16:1719973. doi: 10.3389/fmicb.2025.1719973 (PMC12835223; doi:10.3389/fmicb.2025.1719973)
Supplement: Supplementary file 4 [file Table_4.docx]

1

TCGGGGTGCTATAATGCAGTCGAGCGAACAGATAAGGAGCTTGCTCCTTTGACGTTAGCGGCGGACGGGTGAGTAACACGTGGGTAACCTACCTATAAGACTGGAATAACTCCGGGAAACCGGGGCTAATGCCGGATAACATGTTGAACCGCATGGTTCTACAGTGAAAGACGGTCTTGCTGTCACTTATAGATGGACCCGCGCCGTATTAGCTAGTTGGTGGGGTAACGGCCTACCAAGGCGACGATACGTAGCCGACCTGAGAGGGTGATCGGCCACACTGGAACTGAGACACGGTCCAGACTCCTACGGGAGGCAGCAGTAGGGAATCTTCCGCAATGGGCGAAAGCCTGACGGAGCAACGCCGCGTGAGTGATGAAGGTCTTCGGATCGTAAAGCTCTGTTGTTAGGGAAGAACAAATGTGTAAGTAACTGTGCACATCTTGACGGTACCTAACCAGAAAGCCACGGCTAACTACGTGCCAGCAGCCGCGGTAATACGTAGGTGGCAAGCGTTATCCGGAATTATTGGGCGTAAAGCGCGCGTAGGCGGTTTTTTAAGTCTGATGTGAAAGCCCACGGCTCAACCGTGGAGGGTCATTGGAAACTGGAAAACTTGAGTGCAGAAGAGGAAAGTGGAATTCCATGTGTAGCGGTGAAATGCGCAGAGATATGGAGGAACACCAGTGGCGAAGGCGGCTTTCTGGTCTGCAACTGACGCTGATGTGCGAAAGCGTGGGGATCAAACAGGATTAGATACCCTGGTAGTCCACGCCGTAAACGATGAGTGCTAAGTGTTAGGGGGTTTCCGCCCCTTAGTGCTGCAGCTAACGCATTAAGCACTCCGCCTGGGGAGTACGGTCGCAAGACTGAAACTCAAAGGAATTGACGGGGACCCGCACAAGCGGGGAAGA

2

GGGGGCGTGCTATAATGCAGTCGAGCGAATGGATTGAGAGCTTGCTCTCAAGAAGTTAGCGGCGGACGGGTGAGTAACACGTGGGTAACCTGCCCATAAGACTGGGATAACTCCGGGAAACCGGGGCTAATACCGGATAACATTTTGAACTGCATGGTTCGAAATTGAAAGGCGGCTTCGGCTGTCACTTATGGATGGACCCGCGTCGCATTAGCTAGTTGGTGAGGTAACGGCTCACCAAGGCAACGATGCGTAGCCGACCTGAGAGGGTGATCGGCCACACTGGGACTGAGACACGGCCCAGACTCCTACGGGAGGCAGCAGTAGGGAATCTTCCGCAATGGACGAAAGTCTGACGGAGCAACGCCGCGTGAGTGATGAAGGCTTTCGGGTCGTAAAACTCTGTTGTTAGGGAAGAACAAGTGCTAGTTGAATAAGCTGGCACCTTGACGGTACCTAACCAGAAAGCCACGGCTAACTACGTGCCAGCAGCCGCGGTAATACGTAGGTGGCAAGCGTTATCCGGAATTATTGGGCGTAAAGCGCGCGCAGGTGGTTTCTTAAGTCTGATGTGAAAGCCCACGGCTCAACCGTGGAGGGTCATTGGAAACTGGGAGACTTGAGTGCAGAAGAGGAAAGTGGAATTCCATGTGTAGCGGTGAAATGCGTAGAGATATGGAGGAACACCAGTGGCGAAGGCGACTTTCTGGTCTGTAACTGACACTGAGGCGCGAAAGCGTGGGGAGCAAACAGGATTAGATACCCTGGTAGTCCACGCCGTAAACGATGAGTGCTAAGTGTTAGAGGGTTTCCGCCCTTTAGTGCTGAAGTTAACGCATTAAGCACTCCGCCTGGGGAGTACGGCCGCAAGGCTGAAACTCAAAGGAATTGACGGGGGCCCGCACAAGCGGTGGAACATGTGGTTTAATTCC

3

TAAGTGTGCTATAATGCAGTCGAGCGAACAGATAAGGAGCTTGCTCCTTTGACGTTAGCGGCGGACGGGTGAGTAACACGTGGGTAACCTACCTATAAGACTGGAATAACTCCGGGAAACCGGGGCTAATGCCGGATAACATGTTGAACCGCATGGTTCTACAGTGAAAGACGGTCTTGCTGTCACTTATAGATGGACCCGCGCCGTATTAGCTAGTTGGTGGGGTAACGGCCTACCAAGGCGACGATACGTAGCCGACCTGAGAGGGTGATCGGCCACACTGGAACTGAGACACGGTCCAGACTCCTACGGGAGGCAGCAGTAGGGAATCTTCCGCAATGGGCGAAAGCCTGACGGAGCAACGCCGCGTGAGTGATGAAGGTCTTCGGATCGTAAAGCTCTGTTGTTAGGGAAGAACAAATGTGTAAGTAACTGTGCACATCTTGACGGTACCTAACCAGAAAGCCACGGCTAACTACGTGCCAGCAGCCGCGGTAATACGTAGGTGGCAAGCGTTATCCGGAATTATTGGGCGTAAAGCGCGCGTAGGCGGTTTTTTAAGTCTGATGTGAAAGCCCACGGCTCAACCGTGGAGGGTCATTGGAAACTGGAAAACTTGAGTGCAGAAGAGGAAAGTGGAATTCCATGTGTAGCGGTGAAATGCGCAGAGATATGGAGGAACACCAGTGGCGAAGGCGGCTTTCTGGTCTGCAACTGACGCTGATGTGCGAAAGCGTGGGGATCAAACAGGATTAGATACCCTGGTAGTCCACGCCGTAAACGATGAGTGCTAAGTGTTAGGGGGTTTCCGCCCCTTAGTGCTGCAGCTAACGCATTAAGCACTCCGCCTGGGGAGTACGGTCGCAAGACTGAAACTCAAAGGAATTGACGGGGACCCGCACAAGCGGTGGAACATGTGGTTTAATTCCAAGCAACGCGAAGAACCTTACCAAATCTTGAAATCCTTTG

4

CGGAGAGTGCTATAATGCAGTCGAGCGAACAGATAAGGAGCTTGCTCCTTTGACGTTAGCGGCGGACGGGTGAGTAACACGTGGGTAACCTACCTATAAGACTGGAATAACTCCGGGAAACCGGGGCTAATGCCGGATAACATGTTGAACCGCATGGTTCTACAGTGAAAGACGGTCTTGCTGTCACTTATAGATGGACCCGCGCCGTATTAGCTAGTTGGTGGGGTAACGGCCTACCAAGGCGACGATACGTAGCCGACCTGAGAGGGTGATCGGCCACACTGGAACTGAGACACGGTCCAGACTCCTACGGGAGGCAGCAGTAGGGAATCTTCCGCAATGGGCGAAAGCCTGACGGAGCAACGCCGCGTGAGTGATGAAGGTCTTCGGATCGTAAAGCTCTGTTGTTAGGGAAGAACAAATGTGTAAGTAACTGTGCACATCTTGACGGTACCTAACCAGAAAGCCACGGCTAACTACGTGCCAGCAGCCGCGGTAATACGTAGGTGGCAAGCGTTATCCGGAATTATTGGGCGTAAAGCGCGCGTAGGCGGTTTTTTAAGTCTGATGTGAAAGCCCACGGCTCAACCGTGGAGGGTCATTGGAAACTGGAAAACTTGAGTGCAGAAGAGGAAAGTGGAATTCCATGTGTAGCGGTGAAATGCGCAGAGATATGGAGGAACACCAGTGGCGAAGGCGGCTTTCTGGTCTGCAACTGACGCTGATGTGCGAAAGCGTGGGGATCAAACAGGATTAGATACCCTGGTAGTCCACGCCGTAAACGATGAGTGCTAAGTGTTAGGGGGTTTCCGCCCCTTAGTGCTGCAGCTAACGCATTAAGCACTCCGCCTGGGGAATACGGTCGCAAGACTGAAACTCAAAGGAATTGACGGGGACCCGCACCACGGGGGAGAATGTGGGTTTATTCCAAGCAAGCCGAGAAA

5

CGGGGTAGGACTACCATGCAGTCGAACGGTAACAGGAAGCAGCTTGCTGCTTTGCTGACGAGTGGCGGACGGGTGAGTAATGTCTGGGAAACTGCCTGATGGAGGGGGATAACTACTGGAAACGGTAGCTAATACCGCATAACGTCGCAAGACCAAAGAGGGGGACCTTCGGGCCTCTTGCCATCGGATGTGCCCAGATGGGATTAGCTAGTAGGTGGGGTAAAGGCTCACCTAGGCGACGATCCCTAGCTGGTCTGAGAGGATGACCAGCCACACTGGAACTGAGACACGGTCCAGACTCCTACGGGAGGCAGCAGTGGGGAATATTGCACAATGGGCGCAAGCCTGATGCAGCCATGCCGCGTGTATGAAGAAGGCCTTCGGGTTGTAAAGTACTTTCAGCGGGGAGGAAGGGAGTAAAGTTAATACCTTTGCTCATTGACGTTACCCGCAGAAGAAGCACCGGCTAACTCCGTGCCAGCAGCCGCGGTAATACGGAGGGTGCAAGCGTTAATCGGAATTACTGGGCGTAAAGCGCACGCAGGCGGTTTGTTAAGTCAGATGTGAAATCCCCGGGCTCAACCTGGGAACTGCATCTGATACTGGCAAGCTTGAGTCTCGTAGAGGGGGGTAGAATTCCAGGTGTAGCGGTGAAATGCGTAGAGATCTGGAGGAATACCGGTGGCGAAGGCGGCCCCCTGGACGAAGACTGACGCTCAGGTGCGAAAGCGTGGGGAGCAAACAGGATTAGATACCCTGGTAGTCCACGCCGTAAACGATGTCGACTTGGAGGTTGTGCCCTTGAGGCGTGGCTTCCGGAGCTAACGCGTTAAGTCGACCGCCTGGGGAGTACGGCCGCAAGGTTAAAACTCAAATGAATTGACGGGGGCCCGCACAAGCGGGGAACATGTGGGTTATTCCATGGACGCGAAA

6

CGTGCTGTGCTATAATGCAGTCGAACGCTTCTTTCCTCCCGAGTGCTTGCACTCAATTGGAAAGAGGAGTGGCGGACGGGTGAGTAACACGTGGGTAACCTACCCATCAGAGGGGGATAACACTTGGAAACAGGTGCTAATACCGCATAACAGTTTATGCCGCATGGCATAAGAGTGAAAGGCGCTTTCGGGTGTCGCTGATGGATGGACCCGCGGTGCATTAGCTAGTTGGTGAGGTAACGGCTCACCAAGGCCACGATGCATAGCCGACCTGAGAGGGTGATCGGCCACACTGGGACTGAGACACGGCCCAGACTCCTACGGGAGGCAGCAGTAGGGAATCTTCGGCAATGGACGAAAGTCTGACCGAGCAACGCCGCGTGAGTGAAGAAGGTTTTCGGATCGTAAAACTCTGTTGTTAGAGAAGAACAAGGACGTTAGTAACTGAACGTCCCCTGACGGTATCTAACCAGAAAGCCACGGCTAACTACGTGCCAGCAGCCGCGGTAATACGTAGGTGGCAAGCGTTGTCCGGATTTATTGGGCGTAAAGCGAGCGCAGGCGGTTTCTTAAGTCTGATGTGAAAGCCCCCGGCTCAACCGGGGAGGGTCATTGGAAACTGGGAGACTTGAGTGCAGAAGAGGAGAGTGGAATTCCATGTGTAGCGGTGAAATGCGTAGATATATGGAGGAACACCAGTGGCGAAGGCGGCTCTCTGGTCTGTAACTGACGCTGAGGCTCGAAAGCGTGGGGAGCAAACAGGATTAGATACCCTGGTAGTCCACGCCGTAAACGATGAGTGCTAAGTGTTGGAGGGTTTCCGCCCTTCAGTGCTGCAGCAAACGCATTAAGCACTCCGCCTGGGGAATACGACCGCAAGGTTGAAACTCAAAGGAATTGACGGGGGCCCGCCCAAGGGGGGAAGATGTGGGTTTATTCCAAGCA

7

GGGGGTGTGCTATAATGCAGTCGAGCGAACAGATAAGGAGCTTGCTCCTTTGACGTTAGCGGCGGACGGGTGAGTAACACGTGGGTAACCTACCTATAAGACTGGAATAACTCCGGGAAACCGGGGCTAATGCCGGATAACATGTTGAACCGCATGGTTCTACAGTGAAAGACGGTCTTGCTGTCACTTATAGATGGACCCGCGCCGTATTAGCTAGTTGGTGGGGTAACGGCCTACCAAGGCGACGATACGTAGCCGACCTGAGAGGGTGATCGGCCACACTGGAACTGAGACACGGTCCAGACTCCTACGGGAGGCAGCAGTAGGGAATCTTCCGCAATGGGCGAAAGCCTGACGGAGCAACGCCGCGTGAGTGATGAAGGTCTTCGGATCGTAAAGCTCTGTTGTTAGGGAAGAACAAATGTGTAAGTAACTGTGCACATCTTGACGGTACCTAACCAGAAAGCCACGGCTAACTACGTGCCAGCAGCCGCGGTAATACGTAGGTGGCAAGCGTTATCCGGAATTATTGGGCGTAAAGCGCGCGTAGGCGGTTTTTTAAGTCTGATGTGAAAGCCCACGGCTCAACCGTGGAGGGTCATTGGAAACTGGAAAACTTGAGTGCAGAAGAGGAAAGTGGAATTCCATGTGTAGCGGTGAAATGCGCAGAGATATGGAGGAACACCAGTGGCGAAGGCGGCTTTCTGGTCTGCAACTGACGCTGATGTGCGAAAGCGTGGGGATCAAACAGGATTAGATACCCTGGTAGTCCACGCCGTAAACGATGAGTGCTAAGTGTTAGGGGGTTTCCGCCCCTTAGTGCTGCAGCTAACGCATTAAGCACTCCGCCTGGGGAGTACGGTCGCAAGACTGAAACTCAAAGGAATTGACGGGGACCCGCACAAGCGGTGGAACATGTGGTTTAATTCCAAGCAACGCGAAGAACCTTACCAAATCTTGAATCCTTTGACC

8

GCCGTGCTCGTGCTACACTGCAGTAGAACGCTGAGGACAGGTGCTTGCACTATTCTAAGGAGTTGCGAACGGGTGAGTAACGCGTGGGTGACCTACCTGATAGCGGAGGATAACTATTGGAAACGATAGCTAATACCGCATAAAAGTGCTTAACACATGTTAAGAACTTACCAGGGGCAACTGCTCCACTATGAGATGGACCTGCGTTGTATTAGCTAGTTGGTGAGGTAAAGGCTCACCAAGGCGACGATACATAGCCGACCTGAGAGGGTGATCGGCCACACTGGGACTGAGACACGGCCCAGACTCCTACGGGAGGCAGCAGTAGGGAATCTTCGGCAATGGGGGGAACCCTGACCGAGCAACGCCGCGTGAGTGAAGAAGGTTTTCGGATCGTAAAGCTCTGTTGTTAGAGAAGAACGTTAATGGGAGTGGAAAACCCATTATGTGACGGAAACTAACCAGAAAGGGACGGCTAACTACGTGCCAGCAGCCGCGGTAATACGTAGGTCCCGAGCGTTGTCCGGATTTATTGGGCGTAAAGCGAGCGCAGGCGCTTCTTTAAGTCTGAAGATAAAGGCAGTGGCTCAGCCATTGTACGCTTTGAAAACTGGAGAACTTGAGTGCAGAAGGGGAGAGTGTAATTCCATGAGTAGCGGAGAGATGCGTAGATATATGGAGGAACACCGGTGGCGAAAGCGCCTCTCTGGTCTGTAACTGACGCTGAGGCTCGAAAGCGTGAGGAGCAGACAGGATGAGATACCCTGCTACTCCACGTCGTAAACGATGAGTTGCTAGGCTGTTACGTCTCTTTTCCGGCGCTCAAAGCCGCAGCTAACGACATTTACTGCACTCCGACCTGGCGCAGCGACGTACCGACAAGGCATTGAAATCTGTA

8’

TCGGTGTGCTATACTGCAGTAGAACGCTGAGGACAGGTGCTTGCACTAGTCTAAGGAGTTGCGAACGGGTGAGTAACGCGTAGGTAACCTACCTGATAGCGGGGGATAACTATTGGAAACGATAGCTAATACCGCATAAAAGTGCTTAACACATGTTAAGAACTTAAAAGGGGCAACTGCTCCACTATGAGATGGACCTGCGTTGTATTAGCTAGTTGGTGAGGTAAAGGCTCACCAAGGCGACGATACATAGCCGACCTGAGAGGGTGATCGGCCACACTGGGACTGAGACACGGCCCAGACTCCTACGGGAGGCAGCAGTAGGGAATCTTCGGCAATGGGGGGAACCCTGACCGAGCAACGCCGCGTGAGTGAAGAAGGTTTTCGGATCGTAAAGCTCTGTTGTTAGAGAAGAACGGTAATGGGAGTGGAAAACCCATTATGTGACGGTAACTAACCAGAAAGGGACGGCTAACTACGTGCCAGCAGCCGCGGTAATACGTAGGTCCCGAGCGTTGTCCGGATTTATTGGGCGTAAAGCGAGCGCAGGCGGTTCTTTAAGTCTGAAGTTAAAGGCAGTGGCTCAACCATTGTACGCTTTGGAAACTGGAGAACTTGAGTGCAGAAGGGGAGAGTGGAATTCCATGTGTAGCGGTGAAATGCGTAGATATATGGAGGAACACCGGTGGCGAAAGCGGCTCTCTGGTCTGTAACTGACGCTGAGGCTCGAAAGCGTGGGGAGCAAACAGGATTAGATACCCTGGTAGTCCACGCCGTAAACGATGAGTGCTAGGTGTTAGGCCCTTTCCGGGGCTTAGTGCCGGAGCTAACGCATTAAGCACTCCGCCTGGGGAGTACGACCGCAAGGTTGAAACTCAAAGGAATTGACGGGGGCCCGCACAAGCGGTGGAGCATGTGGTTTAATTCGAAGCAACGCGAAGAACCTTACCAGGTCTTGACCTCCCGATGCCCGCTCTAGAGATAGAGTTTACTTCGGACCTCGGGGAAGGGGGGGCATGGTTGCCCCCACTCCGGGC

9

TGGCGTGTGCTATAATGCAGTCGAGCGAACAGACGAGGAGCTTGCTCCTCTGACGTTAGCGGCGGACGGGTGAGTAACACGTGGATAACCTACCTATAAGACTGGGATAACTTCGGGAAACCGGAGCTAATACCGGATAATATATTGAACCGCATGGTTCAATAGTGAAAGACGGTTTTGCTGTCACTTATAGATGGATCCGCGCCGCATTAGCTAGTTGGTAAGGTAACGGCTTACCAAGGCAACGATGCGTAGCCGACCTGAGAGGGTGATCGGCCACACTGGAACTGAGACACGGTCCAGACTCCTACGGGAGGCAGCAGTAGGGAATCTTCCGCAATGGGCGAAAGCCTGACGGAGCAACGCCGCGTGAGTGATGAAGGTCTTCGGATCGTAAAACTCTGTTATTAGGGAAGAACAAATGTGTAAGTAACTATGCACGTCTTGACGGTACCTAATCAGAAAGCCACGGCTAACTACGTGCCAGCAGCCGCGGTAATACGTAGGTGGCAAGCGTTATCCGGAATTATTGGGCGTAAAGCGCGCGTAGGCGGTTTTTTAAGTCTGATGTGAAAGCCCACGGCTCAACCGTGGAGGGTCATTGGAAACTGGAAAACTTGAGTGCAGAAGAGGAAAGTGGAATTCCATGTGTAGCGGTGAAATGCGCAGAGATATGGAGGAACACCAGTGGCGAAGGCGACTTTCTGGTCTGTAACTGACGCTGATGTGCGAAAGCGTGGGGATCAAACAGGATTAGATACCCTGGTAGTCCACGCCGTAAACGATGAGTGCTAAGTGTTAGGGGGTTTCCGCCCCTTAGTGCTGCAGCTAACGCATTAAGCACTCCGCCTGGGGAGTACGACCGCAAGGTTGAAACTCAAAGGAATTGACGGGGACCCGCCCAAGCGGTGGAGGA

10

GTGCGAGTGCTATAATGCAGTCGAGCGAATGGATTAAGAGCTTGCTCTTATGAAGTTAGCGGCGGACGGGTGAGTAACACGTGGGTAACCTGCCCATAAGACTGGGATAACTCCGGGAAACCGGGGCTAATACCGGATAACATTTTGAACCGCATGGTTCGAAATTGAAAGGCGGCTTCGGCTGTCACTTATGGATGGACCCGCGTCGCATTAGCTAGTTGGTGAGGTAACGGCTCACCAAGGCAACGATGCGTAGCCGACCTGAGAGGGTGATCGGCCACACTGGGACTGAGACACGGCCCAGACTCCTACGGGAGGCAGCAGTAGGGAATCTTCCGCAATGGACGAAAGTCTGACGGAGCAACGCCGCGTGAGTGATGAAGGCTTTCGGGTCGTAAAACTCTGTTGTTAGGGAAGAACAAGTGCTAGTTGAATAAGCTGGCACCTTGACGGTACCTAACCAGAAAGCCACGGCTAACTACGTGCCAGCAGCCGCGGTAATACGTAGGTGGCAAGCGTTATCCGGAATTATTGGGCGTAAAGCGCGCGCAGGTGGTTTCTTAAGTCTGATGTGAAAGCCCACGGCTCAACCGTGGAGGGTCATTGGAAACTGGGAGACTTGAGTGCAGAAGAGGAAAGTGGAATTCCATGTGTAGCGGTGAAATGCGTAGAGATATGGAGGAACACCAGTGGCGAAGGCGACTTTCTGGTCTGTAACTGACACTGAGGCGCGAAAGCGTGGGGAGCAAACAGGATTAGATACCCTGGTAGTCCACGCCGTAAACGATGAGTGCTAAGTGTTAGAGGGTTTCCGCCCTTTAGTGCTGAAGTTAACGCATTAAGCACTCCGCCTGGGGAGTACGGCCGCAAGGCTGAAACTCAAAGGAATTGACGGGGGCCCGCACAAGCGGTGGAGCATGTGGTTTAATTCGAAGCAACGCGAAGAACCTTACCAGGGCTTGACTCCTCCTGAAAACCCAAAGAATAGGGTTCTCCTTCGGGAACAAAAGGCAGGGGGGGCTTGGTGTCCCCCCTCTCGGCCCGGAAAGTTGGGTAAACCCCCCAAAAGGGGACCCT

11

ATGCGTGTGCTATAATGCAGTCGAGCGAACAGATAAGGAGCTTGCTCCTTTGACGTTAGCGGCGGACGGGTGAGTAACACGTGGGTAACCTACCTATAAGACTGGAATAACTCCGGGAAACCGGGGCTAATGCCGGATAACATGTTGAACCGCATGGTTCTACAGTGAAAGACGGTCTTGCTGTCACTTATAGATGGACCCGCGCCGTATTAGCTAGTTGGTGGGGTAACGGCCTACCAAGGCGACGATACGTAGCCGACCTGAGAGGGTGATCGGCCACACTGGAACTGAGACACGGTCCAGACTCCTACGGGAGGCAGCAGTAGGGAATCTTCCGCAATGGGCGAAAGCCTGACGGAGCAACGCCGCGTGAGTGATGAAGGTCTTCGGATCGTAAAGCTCTGTTGTTAGGGAAGAACAAATGTGTAAGTAACTGTGCACATCTTGACGGTACCTAACCAGAAAGCCACGGCTAACTACGTGCCAGCAGCCGCGGTAATACGTAGGTGGCAAGCGTTATCCGGAATTATTGGGCGTAAAGCGCGCGTAGGCGGTTTTTTAAGTCTGATGTGAAAGCCCACGGCTCAACCGTGGAGGGTCATTGGAAACTGGAAAACTTGAGTGCAGAAGAGGAAAGTGGAATTCCATGTGTAGCGGTGAAATGCGCAGAGATATGGAGGAACACCAGTGGCGAAGGCGGCTTTCTGGTCTGCAACTGACGCTGATGTGCGAAAGCGTGGGGATCAAACAGGATTAGATACCCTGGTAGTCCACGCCGTAAACGATGAGTGCTAAGTGTTAGGGGGTTTCCGCCCCTTAGTGCTGCAGCTAACGCATTAAGCACTCCGCCTGGGGAGTACGGTCGCAAGACTGAAACTCAAAGGAATTGACGGGGACCCGCACAAGCGGGGGAGCATGTGGTTTAATTCGAAGCAACGCGAAGAACCTTACCAAATCTTGAAATCCTTTGAACCGTCTAAAA

12

GGCGTCGGCTTACCATGCAGTCGAACGGTAGCAGGAGAAAGCTTGCTTTCTTGCTGACGAGTGGCGGACGGGTGAGTAATGCTTGGGAATCTGGCTTATGGAGGGGGATAACTACTGGAAACGGTAGCTAATACCGCGTAATATCGAGAGATTAAAGACTGGGACCTACGGGCCAGTTGCCATAAGATGAGCCCAAGTGGGATTAGGTAGTTGGTGGGGTAAAGGCCTACCAAGCCCGCGATCTCTAGCTGGTCTGAGAGGATGACCAGCCACACTGGAACTGAGACACGGTCCAGACTCCTACGGGAGGCAGCAGTGGGGAATATTGCACAATGGGGGGAACCCTGATGCAGCCATGCCGCGTGAATGAAGAAGGCCTTCGGGTTGTAAAGTTCTTTCGGTGGTGAGGAAGGTATCAACTTTAATAGAGTTGGTAATTGACGTTATCCACAGAAGAAGCACCGGCTAACTCCGTGCCAGCAGCCGCGGTAATACGGAGGGTGCGAGCGTTAATCGGAATGACTGGGCGTAAAGGGCACGCAGGCGGATTGTTAAGTGAGATGTGAAAGCCCCGGGCTTAACCTGGGAATTGCATTTCAGACTGGCAATCTAGAGTATTTTAGGGAGGGGTAGAATTCCACGTGTAGCGGTGAAATGCGTAGAGATGTGGAGGAATACCGAAGGCGAAGGCAGCCCCTTGGGAATATACTGACGCTCATGTGCGAAAGCGTGGGGAGCAAACAGGATTAGATACCCTGGTAGTCCACGCTGTAAACGATGTCGATTTGGGGATTGGGCTATAAGCTTGGTGCCCGAAGCTAACGTGATAAATCGACCGCCTGGGGAGTACGGCCGCAAGGTTAAAACTCAAATGAATTGACGGGGGCCCGCACAAGCGGGGGAGCATGTGGGTTTAATTCGATGCAACGCGAAGAACCTTACCTACTCTTGAATCCC

13

ACTAGAGTGCTATAATGCAGTCGAGCGAACGGACAAGGAGCTTGCTCCTTTGAAGTTAGCGGCGGACGGGTGAGTAACACGTGGGTAACCTACCTATAAGACTGGAATAACTCCGGGAAACCGGGGCTAATGCCGGATAACATGTTGAACCGCATGGTTCAACAGTGAAAGACGGTCTTGCTGTCACTTATAGATGGACCCGCGCCGTATTAGCTAGTTGGTGGGGTAACGGCCTACCAAGGCGACGATACGTAGCCGACCTGAGAGGGTGATCGGCCACACTGGAACTGAGACACGGTCCAGACTCCTACGGGAGGCAGCAGTAGGGAATCTTCCGCAATGGGCGAAAGCCTGACGGAGCAACGCCGCGTGAGTGATGAAGGTCTTCGGATCGTAAAGCTCTGTTGTTAGGGAAGAACAAATGTGTAAGTAACTGTGCACGTCTTGACGGTACCTAACCAGAAAGCCACGGCTAACTACGTGCCAGCAGCCGCGGTAATACGTAGGTGGCAAGCGTTATCCGGAATTATTGGGCGTAAAGCGCGCGTAGGCGGTTTTTTAAGTCTGATGTGAAAGCCCACGGCTCAACCGTGGAGGGTCATTGGAAACTGGAAAACTTGAGTGCAGAAGAGGAAAGTGGAATTCCATGTGTAGCGGTGAAATGCGCAGAGATATGGAGGAACACCAGTGGCGAAGGCGGCTTTCTGGTCTGCAACTGACGCTGATGTGCGAAAGCGTGGGGATCAAACAGGATTAGATACCCTGGTAGTCCACGCCGTAAACGATGAGTGCTAAGTGTTAGGGGGTTTCCGCCCCTTAGTGCTGCAGCTAACGCATTAAGCACTCCGCCTGGGGAGTACGGTCGCAAGACTGAAACTCAAAGGAATTGACGGGGACCCGCACAAGCGGGGGAACATGTGGGTTTATTCCAAAGAACG

14

ACGTAGGGTGCTATACTGCAGTAGAACGCTGAGGACAGGTGCTTGCACTAGTCTAAGGAGTTGCGAACGGGTGAGTAACGCGTAGGTAACCTACCTGATAGCGGGGGATAACTATTGGAAACGATAGCTAATACCGCATAAAAGTGCTTAACACATGTTAAGAACTTAAAAGGGGCAACTGCTCCACTATGAGATGGACCTGCGTTGTATTAGCTAGTTGGTGAGGTAAAGGCTCACCAAGGCGACGATACATAGCCGACCTGAGAGGGTGATCGGCCACACTGGGACTGAGACACGGCCCAGACTCCTACGGGAGGCAGCAGTAGGGAATCTTCGGCAATGGGGGGAACCCTGACCGAGCAACGCCGCGTGAGTGAAGAAGGTTTTCGGATCGTAAAGCTCTGTTGTTAGAGAAGAACGGTAATGGGAGTGGAAAACCCATTATGTGACGGTAACTAACCAGAAAGGGACGGCTAACTACGTGCCAGCAGCCGCGGTAATACGTAGGTCCCGAGCGTTGTCCGGATTTATTGGGCGTAAAGCGAGCGCAGGCGGTTCTTTAAGTCTGAAGTTAAAGGCATTGGCTCAACCAATGTATGCTTTGGAAACTGGAGAACTTGAGTGCAGAAGGGGAGAGTGGAATTCCATGTGTAGCGGTGAAATGCGTAGATATATGGAGGAACACCGGTGGCGAAAGCGGCTCTCTGGTCTGTAACTGACGCTGAGGCTCGAAAGCGTGGGGAGCAAACAGGATTAGATACCCTGGTAGTCCACGCCGTAAACGATGAGTGCTAGGTGTTAGGCCCTTTCCGGGGCTTAGTGCCGGAGCTAACGCATTAAGCACTCCGCCTGGGGAGTACGACCGCAAGGTTGAAACTCAAAGGAATTGACGGGGGCCCGCACAAGCGGTGGAGCATGTGGTTTAATTCGAAGCAACGCGAAGAACCTTACCAGGTCTTGACTTCCCGATGCCCGTCTAAAGATAAAGTTTACTTCGGACATCGGGGAAGGGGGGGCTGGGTTGCCCA

15

GGGCGGGTGCTATAATGCAGTAGAACGCTGAGGACAGGTGCTTGCACTAGTCTAAGGAGTTGCGAACGGGTGAGTAACGCGTAGGTAACCTACCTGATAGCGGGGGATAACTATTGGAAACGATAGCTAATACCGCATAAAAGTGCTTAACACATGTTAAGAACTTAAAAGGGGCAACTGCTCCACTATGAGATGGACCTGCGTTGTATTAGCTAGTTGGTGAGGTAAAGGCTCACCAAGGCGACGATACATAGCCGACCTGAGAGGGTGATCGGCCACACTGGGACTGAGACACGGCCCAGACTCCTACGGGAGGCAGCAGTAGGGAATCTTCGGCAATGGGGGGAACCCTGACCGAGCAACGCCGCGTGAGTGAAGAAGGTTTTCGGATCGTAAAGCTCTGTTGTTAGAGAAGAACGGTAATGGGAGTGGAAAACCCATTATGTGACGGTAACTAACCAGAAAGGGACGGCTAACTACGTGCCAGCAGCCGCGGTAATACGTAGGTCCCGAGCGTTGTCCGGATTTATTGGGCGTAAAGCGAGCGCAGGCGGTTCTTTAAGTCTGAAGTTAAAGGCATTGGCTCAACCAATGTATGCTTTGGAAACTGGAGAACTTGAGTGCAGAAGGGGAGAGTGGAATTCCATGTGTAGCGGTGAAATGCGTAGATATATGGAGGAACACCGGTGGCGAAAGCGGCTCTCTGGTCTGTAACTGACGCTGAGGCTCGAAAGCGTGGGGAGCAAACAGGATTAGATACCCTGGTAGTCCACGCCGTAAACGATGAGTGCTAGGTGTTAGGCCCTTTCCGGGGCTTAGTGCCGGAGCTAACGCATTAAGCACTCCGCCTGGGGAGTACGACCGCAAGGTTGAAACTCAAAGGAATTGACGGGGGCCCGCACAAGCGGTGGAACATGTGGTTTAATTCCAAGCAACGCGAAGAACCTTACCAGGTCTTGACATCCCGATGCCCGTCTAAAGAAAAAGTTTACTTCCGGACATCGGGGAAGGGGGGGCTGGGTGTCCCCCAC

16

GGGCGTCGGACTACCTGCAGTCGAACGGTAACAGGAAGCAGCTTGCTGCTTTGCTGACGAGTGGCGGACGGGTGAGTAATGTCTGGGAAACTGCCTGATGGAGGGGGATAACTACTGGAAACGGTAGCTAATACCGCATAACGTCGCAAGACCAAAGAGGGGGACCTTCGGGCCTCTTGCCATCGGATGTGCCCAGATGGGATTAGCTAGTAGGTGGGGTAAAGGCTCACCTAGGCGACGATCCCTAGCTGGTCTGAGAGGATGACCAGCCACACTGGAACTGAGACACGGTCCAGACTCCTACGGGAGGCAGCAGTGGGGAATATTGCACAATGGGCGCAAGCCTGATGCAGCCATGCCGCGTGTATGAAGAAGGCCTTCGGGTTGTAAAGTACTTTCAGCGGGGAGGAAGGGAGTAAAGTTAATACCTTTGCTCATTGACGTTACCCGCAGAAGAAGCACCGGCTAACTCCGTGCCAGCAGCCGCGGTAATACGGAGGGTGCAAGCGTTAATCGGAATTACTGGGCGTAAAGCGCACGCAGGCGGTTTGTTAAGTCAGATGTGAAATCCCCGGGCTCAACCTGGGAACTGCATCTGATACTGGCAAGCTTGAGTCTCGTAGAGGGGGTTAGAATTCCAGGTGTAGCGGTGAAATGCGTAGAGATCTGGAGGAATACCGGTGGCGAAGGCGCCCCCTGGACGAAGACTGACGCTCAGGTGCGAAGCGTGTGGAGCAACAGGATTAGATACCTGGTAGTCCACGCCGTAACGATGTCGACTTGGAGGTTGTGCCCTTGAGGCGTGGTTCCGAGCTACGCGTTAGTCGACCGCCTGGGGAGTACGCCGCAGGTAAAATCACTGAATGACGGGGCCCGCCAAGGGGGGAGATGGGTTTATTCG

17

TAAAATACAAAGTGGTAGCGCCCTCCCGAAGGTTAAGCTACCTACTTCTTTTGCAACCCACTCCCATGGTGTGACGGGCGGTGTGTACAAGGCCCGGGAACGTATTCACCGTGGCATTCTGATCCACGATTACTAGCGATTCCGACTTCATGGAGTCGAGTTGCAGACTCCAATCCGGACTACGACGCACTTTATGAGGTCCGCTTGCTCTCGCGAGGTCGCTTCTCTTTGTATGCGCCATTGTAGCACGTGTGTAGCCCTGGTCGTAAGGGCCATGATGACTTGACGTCATCCCCACCTTCCTCCAGTTTATCACTGGCAGTCTCCTTTGAGTTCCCGGCCGGACCGCTGGCAACAAAGGATAAGGGTTGCGCTCGTTGCGGGACTTAACCCAACATTTCACAACACGAGCTGACGACAGCCATGCAGCACCTGTCTCACAGTTCCCGAAGGCACCAATCCATCTCTGGAAAGTTCTGTGGATGTCAAGACCAGGTAAGGTTCTTCGCGTTGCATCGAATTAAACCACATGCTCCACCGCTTGTGCGGGCCCCCGTCAATTCATTTGAGTTTTAACCTTGCGGCCGTACTCCCCAGGCGGTCGACTTAACGCGTTAGCTCCGGAAGCCACGCCTCAAGGGCACAACCTCCAAGTCGACATCGTTTACGGCGTGGACTACCAGGGTATCTAATCCTGTTTGCTCCCCACGCTTTCGCACCTGAGCGTCAGTCTTCGTCCAGGGGGCCGCCTTCGCCACCGGTATTCCTCCAGATCTCTACGCATTTCACCGCTACACCTGGAATTCTACCCCCCTCTACGAGACTCAAGCTTGCCAGTATCAGATGCAGTTCCCAGGTTGAGCCCGGGGATTTCACATCTGACTTAACAAACCGCCTGCGTGCGCTTTACGCCCAGTAATTCCGATTAACGCTTGCACCCTCCGTAATTACCGCGCTGCTGGCACGGAGTTAGCCGGTGCTTCTTCTGCGGTAACGGCCATGAGCAAAGGATTACTTTTACTCCTTCCTCCCGGTGAAAGACTTTACA

18

TGGTGCAACCTTCGACGGCTAGCTCCAAAATGGTTACTCCACCGGCTTCGGGTGTTACAAACTCTCGTGGTGTGACGGGCGGTGTGTACAAGACCCGGGAACGTATTCACCGTAGCATGCTGATCTACGATTACTAGCGATTCCAGCTTCATGTAGTCGAGTTGCAGACTACAATCCGAACTGAGAACAACTTTATGGGATTTGCTTGACCTCGCGGTTTCGCTGCCCTTTGTATTGTCCATTGTAGCACGTGTGTAGCCCAAATCATAAGGGGCATGATGATTTGACGTCATCCCCACCTTCCTCCGGTTTGTCACCGGCAGTCAACTTAGAGTGCCCAACTGAATGATGGCAACTAAGTTCAAGGGTTGCGCTCGTTGCGGGACTTAACCCAACATCTCACGACACGAGCTGACGACAACCATGCACCACCTGTCACTTTGTCCTCCGAAGAGGAAAACTCTATCTCTAGAGCGGTCAAAGGATGTCAAGATTTGGTAAGGTTCTTCGCGTTGCTTCGAATTAAACCACATGCTCCACCGCTTGTGCGGGTCCCCGTCAATTCCTTTGAGTTTCAGTCTTGCGACCGTACTCCCCAGGCGGAGTGCTTAATGCGTTAGCTGCAGCACTAAGGGGCGGAAACCCCCTAACACTTAGCACTCATCGTTTACGGCGTGGACTACCAGGGTATCTAATCCTGTTTGATCCCCACGCTTTCGCACATCAGCGTCAGTTGCAGACCAGAAAGCCGCCTTCGCCACTGGTGTTCCTCCATATCTCTGCGCATTTCACCGCTACACATGGAATTCCACTTTCCTCTTCTGCACTCAAGTTTTCCAGTTTCCAATGACCCTCCACGGTTGAGCCGTGGGCTTTCCATCAGACTTAAAAACCGCCTACGCGCGTTTACGCCAATAATTCCGGAAACGCTTGCCCCTACGTATACCGCGCTGCTGGACGTAGTAGCCGTGCTTCTGGTAGGACCGCAGATTGACAGTACTACACTTTGTCTTCCTACAAAGAGTTTACATCG

19

GAATTCCAAGTGGTAAGCGCCCTCCCGAAGGTTAAGCTACCTACTTCTTTTGCAACCCACTCCCATGGTGTGACGGGCGGTGTGTACAAGGCCCGGGAACGTATTCACCGTGGCATTCTGATCCACGATTACTAGCGATTCCGACTTCATGGAGTCGAGTTGCAGACTCCAATCCGGACTACGACGCACTTTATGAGGTCCGCTTGCTCTCGCGAGGTCGCTTCTCTTTGTATGCGCCATTGTAGCACGTGTGTAGCCCTGGTCGTAAGGGCCATGATGACTTGACGTCATCCCCACCTTCCTCCAGTTTATCACTGGCAGTCTCCTTTGAGTTCCCGGCCGGACCGCTGGCAACAAAGGATAAGGGTTGCGCTCGTTGCGGGACTTAACCCAACATTTCACAACACGAGCTGACGACAGCCATGCAGCACCTGTCTCACAGTTCCCGAAGGCACCAATCCATCTCTGGAAAGTTCTGTGGATGTCAAGACCAGGTAAGGTTCTTCGCGTTGCATCGAATTAAACCACATGCTCCACCGCTTGTGCGGGCCCCCGTCAATTCATTTGAGTTTTAACCTTGCGGCCGTACTCCCCAGGCGGTCGACTTAACGCGTTAGCTCCGGAAGCCACGCCTCAAGGGCACAACCTCCAAGTCGACATCGTTTACGGCGTGGACTACCAGGGTATCTAATCCTGTTTGCTCCCCACGCTTTCGCACCTGAGCGTCAGTCTTCGTCCAGGGGGCCGCCTTCGCCACCGGTATTCCTCCAGATCTCTACGCATTTCACCGCTACACCTGGAATTCTACCCCCCTCTACGAGACTCAAGCTTGCCAGTATCAGATGCAGTTCCCAGGTTGAGCCCGGGGATTCCCATCTGACTTACAAACCGCCTGCGTGCGTTTACGCCAGTATTCCGATAACGCTTGCACCTCCGATTACCGCGCTGCTGGACGGAGTAGCCGTGCTTTTCTGCGGTACGTCATGAGAAAGGATTACTTACTCCTTCTCCCCGTGAAGACTTAAACCGAAGCTTT

20

TTCTTGACACCTTCGGCGGCTGGCTCCATAAAGGTTACCTCACCGACTTCGGGTGTTGCAAACTCTCGTGGTGTGACGGGCGGTGTGTACAAGGCCCGGGAACGTATTCACCGCGGCATGCTGATCCGCGATTACTAGCGATTCCAGCTTCACGCAGTCGAGTTGCAGACTGCGATCCGAACTGAGAACAGATTTATGGGATTGGCTAAACCTTGCGGTCTTGCAGCCCTTTGTTCTGTCCATTGTAGCACGTGTGTAGCCCAGGTCATAAGGGGCATGATGATTTGACGTCATCCCCACCTTCCTCCGGTTTGTCACCGGCAGTCACCTTAGAGTGCCCAACTGAATGCTGGCAACTAAGATCAAGGGTTGCGCTCGTTGCGGGACTTAACCCAACATCTCACGACACGAGCTGACGACAACCATGCACCACCTGTCACTCTGTCCCCGAAGGGAAAGCCCTATCTCTAGGGTTGTCAGAGGATGTCAAGACCTGGTAAGGTTCTTCGCGTTGCTTCGAATTAAACCACATGCTCCACCGCTTGTGCGGGCCCCCGTCAATTCCTTTGAGTTTCAGTCTTGCGACCGTACTCCCCAGGCGGAGTGCTTAATGCGTTAGCTGCAGCACTAAGGGGCGGAAACCCCCTAACACTTAGCACTCATCGTTTACGGCGTGGACTACCAGGGTATCTAATCCTGTTCGCTCCCCACGCTTTCGCTCCTCAGCGTCAGTTACAGACCAGAGAGTCGCCTTCGCCACTGGTGTTCCTCCACATCTCTACGCATTTCACCGCTACACGTGGAATTCCACTCTCCTCTTCTGCACTCAAGTTTCCCAGTTTCCAATGACCCTCCCCGGTTGAGCCGGGGGCTTTCACATCAGACTTAAGAAACCGCCTGCGAGCCTTTACGCCCATAATTCCGGACACGCTTGCCCCTACGTTTACCGCGCTGCTGGACGTAGTAGCCGGGGTTTCTGGTAAGTACCGCCAGGTGAAGCAGTACTCTCGACTTGTCTTCCTAAAAAGAGTTTCGATCGAAACTTCTCCTCACGGGGTT

21

CCTCTGACACCTTCGGCGGCTGGCTCCATAAAGGTTACCTCACCGACTTCGGGTGTTGCAAACTCTCGTGGTGTGACGGGCGGTGTGTACAAGGCCCGGGAACGTATTCACCGCGGCATGCTGATCCGCGATTACTAGCGATTCCAGCTTCACGCAGTCGAGTTGCAGACTGCGATCCGAACTGAGAACAGATTTGTGGGATTGGCTAAACCTTGCGGTCTCGCAGCCCTTTGTTCTGTCCATTGTAGCACGTGTGTAGCCCAGGTCATAAGGGGCATGATGATTTGACGTCATCCCCACCTTCCTCCGGTTTGTCACCGGCAGTCACCTTAGAGTGCCCAACTAAATGCTGGCAACTAAGATCAAGGGTTGCGCTCGTTGCGGGACTTAACCCAACATCTCACGACACGAGCTGACGACAACCATGCACCACCTGTCACTCTGTCCCCGAAGGGAAAGCCCTATCTCTAGGGTTGTCAGAGGATGTCAAGACCTGGTAAGGTTCTTCGCGTTGCTTCGAATTAAACCACATGCTCCACCGCTTGTGCGGGCCCCCGTCAATTCCTTTGAGTTTCAGTCTTGCGACCGTACTCCCCAGGCGGAGTGCTTAATGCGTTAGCTGCAGCACTAAGGGGCGGAAACCCCCTAACACTTAGCACTCATCGTTTACGGCGTGGACTACCAGGGTATCTAATCCTGTTCGCTCCCCACGCTTTCGCTCCTCAGCGTCAGTTACAGACCAGAGAGTCGCCTTCGCCACTGGTGTTCCTCCACATCTCTACGCATTTCACCGCTACACGTGGAATTCCACTCTCCTCTTCTGCACTCAAGTTTCCCAGTTTCCAATGACCCTCCCCGGTTGAGCCGGGGGCTTTCCATCAGACTTAAGAAACCGCCTGCGAGCCTTTACGCCCATAATTCCGGACACGCTTGCCCCTACGATTACCGCGCTGCTGGACGTATTAGCCGGGGTTTCTGGTAGGACCGCAAGGGGAGCAGTAC

22

TGGTTGACCTTCGACGGCTAGCTCCAAAATGGTTACTCCACCGGCTTCGGGTGTTACAAACTCTCGTGGTGTGACGGGCGGTGTGTACAAGACCCGGGAACGTATTCACCGTAGCATGCTGATCTACGATTACTAGCGATTCCAGCTTCATGTAGTCGAGTTGCAGACTACAATCCGAACTGAGAACAACTTTATGGGATTTGCTTGACCTCGCGGTTTCGCTGCCCTTTGTATTGTCCATTGTAGCACGTGTGTAGCCCAAATCATAAGGGGCATGATGATTTGACGTCATCCCCACCTTCCTCCGGTTTGTCACCGGCAGTCAACTTAGAGTGCCCAACTGAATGATGGCAACTAAGTTCAAGGGTTGCGCTCGTTGCGGGACTTAACCCAACATCTCACGACACGAGCTGACGACAACCATGCACCACCTGTCACTTTGTCCTCCGAAGAGGAAAACTCTATCTCTAGAGCGGTCAAAGGATGTCAAGATTTGGTAAGGTTCTTCGCGTTGCTTCGAATTAAACCACATGCTCCACCGCTTGTGCGGGTCCCCGTCAATTCCTTTGAGTTTCAGTCTTGCGACCGTACTCCCCAGGCGGAGTGCTTAATGCGTTAGCTGCAGCACTAAGGGGCGGAAACCCCCTAACACTTAGCACTCATCGTTTACGGCGTGGACTACCAGGGTATCTAATCCTGTTTGATCCCCACGCTTTCGCACATCAGCGTCAGTTGCAGACCAGAAAGCCGCCTTCGCCACTGGTGTTCCTCCATATCTCTGCGCATTTCACCGCTACACATGGAATTCCACTTTCCTCTTCTGCACTCAAGTTTTCCAGTTTCCAATGACCCTCCACGGTTGAGCCGTGGGCTTTCACATCAGACTTAAAAAACCGCCTACGCGCGTTTACGCCAATAATTCCGGATACGCTTGCCACTACGATTACCGCGCTGCTGGACGTAGTAGCCGGGGTTTCTGGTAGGACCGTCAGATGGGCCAGTACTTACCATTTGTCTTCCTAAAACGAGTTT

23

CTCATTGCGCCTTAGGCGGCTGGCTCCAAAAGGGTACCTCCCCCGATTCCGGTGGTACCAAATTCCCGGGGGGGGAGGGGGGGGGGGACCAAGGCCGGGAAAGGATTCCCCGGGGGGGGGTGGACCGGGAATAATAACGAATCCGGGTTCCTGGAGGGGAAGTGGAGGCCGGAAACCCAAATGGAAAAAAGTTTTAAAAAATAACTTAACCCCCCGAATTCCCCAATCCGTGGACTTCCCATTGGAACACCGGGGGAACCCCAGGCCTAAAGGGCATGGAGAATTGGACGCCTCCCCCCCCTCCTCCGGGTTGGCCCCGGGAAGCTTGGTAAAAGGGCCCAATGGAAGGAGGGAAATAAAAAAAAAGGGTGGGGTTCGTGGGGGAATTAACCCCACCTTCCCCCAACCCAAGTGGACAACAACCTGGCCCCCCTGGCCCTTTGGCCCCCAAAGGGAAAGTTCAATCCCAAAAGGGGCCAAGGAAGGCCAGAACCGGGAAAGGTCCTCCCGGTGGTTCCAAATAAACCCACTGGTCCCCCGGTTGGGGGGGCCCCCCGCCATTTCTTTGGAGTTCCACCCTGGGGGCCGACCTCCCAAGGGGAAGGGTTAATGGCGTAACTGCCACACTGGAGGGCGGAAAACCTCCCACCCTTAAGACTTCTCCGTTAAGGGGGGGAATAACCAGGGATCTAATCCTGGTTGGTCCCCCCGGTTTTCGAGCTTAACCTTAAGTACCGAACAAAAAAAGCCCGCCTTCCGCACCTGGGTGGTTCCTTCCTTATTATCCTACCGCATTTTCACCGGCTACCACATGGAAATTCCACCTTCTCCTCCTTCTGCACCTCAAGTCCTCCCAGGTTCCCAATTGAACCTCTCCCCGGTTGGGAGCCGGGGGGGGCCTTTTT

24

TTTGGTTCCACCTTCGACGGCTAGCTCCAAAATGGTTACTCCACCGGCTTCGGGTGTTACAAACTCTCGTGGTGTGACGGGCGGTGTGTACAAGACCCGGGAACGTATTCACCGTAGCATGCTGATCTACGATTACTAGCGATTCCAGCTTCATGTAGTCGAGTTGCAGACTACAATCCGAACTGAGAACAACTTTATGGGATTTGCTTGACCTCGCGGTTTCGCTGCCCTTTGTATTGTCCATTGTAGCACGTGTGTAGCCCAAATCATAAGGGGCATGATGATTTGACGTCATCCCCACCTTCCTCCGGTTTGTCACCGGCAGTCAACTTAGAGTGCCCAACTGAATGATGGCAACTAAGTTCAAGGGTTGCGCTCGTTGCGGGACTTAACCCAACATCTCACGACACGAGCTGACGACAACCATGCACCACCTGTCACTTTGTCCTCCGAAGAGGAAAACTCTATCTCTAGAGCGGTCAAAGGATGTCAAGATTTGGTAAGGTTCTTCGCGTTGCTTCGAATTAAACCACATGCTCCACCGCTTGTGCGGGTCCCCGTCAATTCCTTTGAGTTTCAGTCTTGCGACCGTACTCCCCAGGCGGAGTGCTTAATGCGTTAGCTGCAGCACTAAGGGGCGGAAACCCCCTAACACTTAGCACTCATCGTTTACGGCGTGGACTACCAGGGTATCTAATCCTGTTTGATCCCCACGCTTTCGCACATCAGCGTCAGTTGCAGACCAGAAAGCCGCCTTCGCCACTGGTGTTCCTCCATATCTCTGCGCATTTCACCGCTACACATGGAATTCCACTTTCCTCTTCTGCACTCAAGTTTTCCAGTTTCCAATGACCCTCCACGGTTGAGCCGTGGGCTTTCACATCAGACTTAAAAAACCGCCTACGCGCGCTTTACGCCCATAATTCCGGATAACGCTTGCACCTACGTATACCGCGCTGCTGGACGTAGTAGCCGGGGTTTCTGGTTAGGA

25

TTCCAACTTAGGCGGCTGGCTCCAAAAAGGTTACCCCACCGACTTCGGGTGTTACAAACTCTCGTGGTGTGACGGGCGGTGTGTACAAGGCCCGGGAACGTATTCACCGCGGCATGCTGATCCGCGATTACTAGCGATTCCAGCTTCATGTAGGCGAGTTGCAGCCTACAATCCGAACTGAGAACGGTTTTATGAGATTAGCTCCACCTCGCGGTCTTGCAGCTCTTTGTACCGTCCATTGTAGCACGTGTGTAGCCCAGGTCATAAGGGGCATGATGATTTGACGTCATCCCCACCTTCCTCCGGTTTGTCACCGGCAGTCACCTTAGAGTGCCCAACTTAATGATGGCAACTAAGATCAAGGGTTGCGCTCGTTGCGGGACTTAACCCAACATCTCACGACACGAGCTGACGACAACCATGCACCACCTGTCACTCTGCTCCCGAAGGAGAAGCCCTATCTCTAGGGTTTTCAGAGGATGTCAAGACCTGGTAAGGTTCTTCGCGTTGCTTCGAATTAAACCACATGCTCCACCGCTTGTGCGGGCCCCCGTCAATTCCTTTGAGTTTCAGCCTTGCGGCCGTACTCCCCAGGCGGAGTGCTTAATGCGTTAACTTCAGCACTAAAGGGCGGAAACCCTCTAACACTTAGCACTCATCGTTTACGGCGTGGACTACCAGGGTATCTAATCCTGTTTGCTCCCCACGCTTTCGCGCCTCAGTGTCAGTTACAGACCAGAAAGTCGCCTTCGCCACTGGTGTTCCTCCATATCTCTACGCATTTCACCGCTACACATGGAATTCCACTTTCCTCTTCTGCACTCAAGTCTCCCAGTTTCCAATGACCCTCCACGGTTGAGCCGTGGGCTTTCACATCAGACTTAAGAAACCACCTGCGCGCGGTTTACGCCCATAATTCCGGATACGCTTGCCCCTACGTTTACCGCGCTGCTGGAC

26

GGTGACCCTTCGACGGCTAGCTCCAAAATGGTTACTCCACCGGCTTCGGGTGTTACAAACTCTCGTGGTGTGACGGGCGGTGTGTACAAGACCCGGGAACGTATTCACCGTAGCATGCTGATCTACGATTACTAGCGATTCCAGCTTCATGTAGTCGAGTTGCAGACTACAATCCGAACTGAGAACAACTTTATGGGATTTGCTTGACCTCGCGGTTTCGCTGCCCTTTGTATTGTCCATTGTAGCACGTGTGTAGCCCAAATCATAAGGGGCATGATGATTTGACGTCATCCCCACCTTCCTCCGGTTTGTCACCGGCAGTCAACTTAGAGTGCCCAACTGAATGATGGCAACTAAGTTCAAGGGTTGCGCTCGTTGCGGGACTTAACCCAACATCTCACGACACGAGCTGACGACAACCATGCACCACCTGTCACTTTGTCCTCCGAAGAGGAAAACTCTATCTCTAGAGCGGTCAAAGGATGTCAAGATTTGGTAAGGTTCTTCGCGTTGCTTCGAATTAAACCACATGCTCCACCGCTTGTGCGGGTCCCCGTCAATTCCTTTGAGTTTCAGTCTTGCGACCGTACTCCCCAGGCGGAGTGCTTAATGCGTTAGCTGCAGCACTAAGGGGCGGAAACCCCCTAACACTTAGCACTCATCGTTTACGGCGTGGACTACCAGGGTATCTAATCCTGTTTGATCCCCACGCTTTCGCACATCAGCGTCAGTTGCAGACCAGAAAGCCGCCTTCGCCACTGGTGTTCCTCCATATCTCTGCGCATTTCACCGCTACACATGGAATTCCACTTTCCTCTTCTGCACTCAAGTTTTCCAGTTTCCAATGACCCTCCACGGTTGAGCCGTGGGCTTTCACATCAGACTTAAAAAACCGCCTACGCGCGCTTACGCCCATAATTCCGGAAACGCTTGCCCCTACGATTACCGCGCTGCTGGACGAAGTAGCCGGGGTTTCTGGTAGGACCGCAAGAGGGCCAG

27

CGGGTTGACCTTAGGCGGCTAGCTCCTTACGGTTACTCCACCGACTTCGGGTGTTACAAACTCTCGTGGTGTGACGGGCGGTGTGTACAAGGCCCGGGAACGTATTCACCGCGGCATGCTGATCCGCGATTACTAGCGATTCCAGCTTCATGTAGGCGAGTTGCAGCCTACAATCCGAACTGAGAATGGTTTTATGGGATTGGCTTGACCTCGCGGTCTTGCAGCCCTTTGTACCATCCATTGTAGCACGTGTGTAGCCCAGGTCATAAGGGGCATGATGATTTGACGTCATCCCCACCTTCCTCCGGTTTGTCACCGGCAGTCACCTTAGAGTGCCCAACTAAATGCTGGCAACTAAGATCAAGGGTTGCGCTCGTTGCGGGACTTAACCCAACATCTCACGACACGAGCTGACGACAACCATGCACCACCTGTCACTCTGTCCCCCGAAGGGGAACGCTCTATCTCTAGAGTTGTCAGAGGATGTCAAGACCTGGTAAGGTTCTTCGCGTTGCTTCGAATTAAACCACATGCTCCACCGCTTGTGCGGGCCCCCGTCAATTCCTTTGAGTTTCAGTCTTGCGACCGTACTCCCCAGGCGGAGTGCTTAATGCGTTAGCTGCAGCACTAAAGGGCGGAAACCCTCTAACACTTAGCACTCATCGTTTACGGCGTGGACTACCAGGGTATCTAATCCTGTTTGCTCCCCACGCTTTCGCGCCTCAGCGTCAGTTACAGACCAAAAAGCCGCCTTCGCCACTGGTGTTCCTCCACATCTCTACGCATTTCACCGCTACACGTGGAATTCCGCTTTTCTCTTCTGCACTCAAGTTCCCCAGTTTCCAATGACCCTCCACGGTTGAGCCGTGGGCTTTCACATCAGACTTAAGAAACCGCCTGCGCGCGCTTACGCCCATAATTCCGGATACGCTTGCCCCTACGTTTACCGCGCTGCTGGACGAAGTAGCCGGGGTTTCTGGTAGGACCGCAAGGACAGCAGTACTCTCGACTTGTCTTCCTACAACAAGTTTAC

28

CCTTTGTGACCTTCGACGGCTAGCTCCAAATGGTTACTCCACCGGCTTCGGGTGTTACAAACTCTCGTGGTGTGACGGGCGGTGTGTACAAGACCCGGGAACGTATTCACCGTAGCATGCTGATCTACGATTACTAGCGATTCCAGCTTCATGTAGTCGAGTTGCAGACTACAATCCGAACTGAGAACAACTTTATGGGATTTGCTTGACCTCGCGGTTTCGCTACCCTTTGTATTGTCCATTGTAGCACGTGTGTAGCCCAAATCATAAGGGGCATGATGATTTGACGTCATCCCCACCTTCCTCCGGTTTGTCACCGGCAGTCAACTTAGAGTGCCCAACTTAATGATGGCAACTAAGCTTAAGGGTTGCGCTCGTTGCGGGACTTAACCCAACATCTCACGACACGAGCTGACGACAACCATGCACCACCTGTCACTTTGTCCCCCGAAGGGGAAAGCTCTATCTCTAGAGTTGTCAAAGGATGTCAAGATTTGGTAAGGTTCTTCGCGTTGCTTCGAATTAAACCACATGCTCCACCGCTTGTGCGGGTCCCCGTCAATTCCTTTGAGTTTCAACCTTGCGGTCGTACTCCCCAGGCGGAGTGCTTAATGCGTTAGCTGCAGCACTAAGGGGCGGAAACCCCCTAACACTTAGCACTCATCGTTTACGGCGTGGACTACCAGGGTATCTAATCCTGTTTGATCCCCACGCTTTCGCACATCAGCGTCAGTTACAGACCAGAAAGTCGCCTTCGCCACTGGTGTTCCTCCATATCTCTGCGCATTTCACCGCTACACATGGAATTCCACTTTCCTCTTCTGCACTCAAGTTTTCCAGTTTCCAATGACCCTCCACGGTTGAGCCGTGGGCTTCCCATCAGACTTAAAAACCGCCTACGCGCGCTTACGCCCATAATTCCGGATACGCTTGCCCCTACGATTACCGGGCTGCTGGACCTAAT

29

CCTTTGGTCCCCTTCGACGGCTAGCTCCATAAATGGTTACTCCACCGGCTTCGGGTGTTACAAACTCTCGTGGTGTGACGGGCGGTGTGTACAAGACCCGGGAACGTATTCACCGTAGCATGCTGATCTACGATTACTAGCGATTCCAGCTTCATGTAGTCGAGTTGCAGACTACAATCCGAACTGAGAACAACTTTATGGGATTTGCATGACCTCGCGGTTTAGCTGCCCTTTGTATTGTCCATTGTAGCACGTGTGTAGCCCAAATCATAAGGGGCATGATGATTTGACGTCATCCCCACCTTCCTCCGGTTTGTCACCGGCAGTCAACCTAGAGTGCCCAACTTAATGATGGCAACTAAGCTTAAGGGTTGCGCTCGTTGCGGGACTTAACCCAACATCTCACGACACGAGCTGACGACAACCATGCACCACCTGTCACTTTGTCCCCCGAAGGGGAAAGCTCTATCTCTAGAGTGGTCAAAGGATGTCAAGATTTGGTAAGGTTCTTCGCGTTGCTTCGAATTAAACCACATGCTCCACCGCTTGTGCGGGTCCCCGTCAATTCCTTTGAGTTTCAACCTTGCGGTCGTACTCCCCAGGCGGAGTGCTTAATGCGTTAGCTGCAGCACTAAGGGGCGGAAACCCCCTAACACTTAGCACTCATCGTTTACGGCGTGGACTACCAGGGTATCTAATCCTGTTTGATCCCCACGCTTTCGCACATCAGCGTCAGTTACAGACCAGAAAGTCGCCTTCGCCACTGGTGTTCCTCCATATCTCTGCGCATTTCACCGCTACACATGGAATTCCACTTTCCTCTTCTGCACTCAAGTTTCCCAGTTTCCAATGACCCTCCACGGTTGAGCCGTGGGTTTTCCATCAGACTTAAGAAACCGCCTACGCGCGTTTAACGCCCATAATTCCGGAAACGCTTGCCCCTACGATTACCGCGCTGCTGGAC

30

GTTTTGGTGACCTTCGACGGCTAGCTCCAAAATGGTTACTCCACCGGCTTCGGGTGTTACAAACTCTCGTGGTGTGACGGGCGGTGTGTACAAGACCCGGGAACGTATTCACCGTAGCATGCTGATCTACGATTACTAGCGATTCCAGCTTCATGTAGTCGAGTTGCAGACTACAATCCGAACTGAGAACAACTTTATGGGATTTGCTTGACCTCGCGGTTTCGCTGCCCTTTGTATTGTCCATTGTAGCACGTGTGTAGCCCAAATCATAAGGGGCATGATGATTTGACGTCATCCCCACCTTCCTCCGGTTTGTCACCGGCAGTCAACTTAGAGTGCCCAACTGAATGATGGCAACTAAGTTCAAGGGTTGCGCTCGTTGCGGGACTTAACCCAACATCTCACGACACGAGCTGACGACAACCATGCACCACCTGTCACTTTGTCCTCCGAAGAGGAAAACTCTATCTCTAGAGCGGTCAAAGGATGTCAAGATTTGGTAAGGTTCTTCGCGTTGCTTCGAATTAAACCACATGCTCCACCGCTTGTGCGGGTCCCCGTCAATTCCTTTGAGTTTCAGTCTTGCGACCGTACTCCCCAGGCGGAGTGCTTAATGCGTTAGCTGCAGCACTAAGGGGCGGAAACCCCCTAACACTTAGCACTCATCGTTTACGGCGTGGACTACCAGGGTATCTAATCCTGTTTGATCCCCACGCTTTCGCACATCAGCGTCAGTTGCAGACCAGAAAGCCGCCTTCGCCACTGGTGTTCCTCCATATCTCTGCGCATTTCACCGCTACACATGGAATTCCACTTTCCTCTTCTGCACTCAAGTTTTCCAGTTTCCAATGACCCTCCACGGTTGAGCCGTGGGCTTTCCATCAGACTTAAAAACCGCCTACGCGCGTTTACGCCCATAATTCCGGATACGCTTGCCCCTACGATTACCGCGCTGCTGGACGTAGTAGCCGGGGTTTCTGGTAGGACCGTCAAATGGGCCAGTACTTACCATTTGTCTTCCTAACACAGAGTTT

31

CTTTGGTGACCTTCGACGGCTAGCTCCATAAATGGTTACTCCACCGGCTTCGGGTGTTACAAACTCTCGTGGTGTGACGGGCGGTGTGTACAAGACCCGGGAACGTATTCACCGTAGCATGCTGATCTACGATTACTAGCGATTCCAGCTTCATGTAGTCGAGTTGCAGACTACAATCCGAACTGAGAACAACTTTATGGGATTTGCATGACCTCGCGGTTTAGCTGCCCTTTGTATTGTCCATTGTAGCACGTGTGTAGCCCAAATCATAAGGGGCATGATGATTTGACGTCATCCCCACCTTCCTCCGGTTTGTCACCGGCAGTCAACCTAGAGTGCCCAACTTAATGATGGCAACTAAGCTTAAGGGTTGCGCTCGTTGCGGGACTTAACCCAACATCTCACGACACGAGCTGACGACAACCATGCACCACCTGTCACTTTGTCCCCCGAAGGGGAAGGCTCTATCTCTAGAGTTTTCAAAGGATGTCAAGATTTGGTAAGGTTCTTCGCGTTGCTTCGAATTAAACCACATGCTCCACCGCTTGTGCGGGTCCCCGTCAATTCCTTTGAGTTTCAACCTTGCGGTCGTACTCCCCAGGCGGAGTGCTTAATGCGTTAGCTGCAGCACTAAGGGGCGGAAACCCCCTAACACTTAGCACTCATCGTTTACGGCGTGGACTACCAGGGTATCTAATCCTGTTTGATCCCCACGCTTTCGCACATCAGCGTCAGTTACAGACCAGAAAGTCGCCTTCGCCACTGGTGTTCCTCCATATCTCTGCGCATTTCACCGCTACACATGGAATTCCACTTTCCTCTTCTGCACTCAAGTTTCCCAGTTTCCAATGACCCTCCACGGTTGAGCCGTGGGCTTTCACATCAGACTTAAGAAACCGCCTACGCGCGCTTTACGCCCAATAATTCCGGATAACGCTTGCCACCTACGTATTACCGCGGCTGCTGGCACGTAATTAGCCGGGGCTTTCTGATAAGGAC

32

GGCTGCCCTTCGACGGCTAGCTCCAAATGGTTACTCCACCGGCTTCGGGTGTTACAAACTCTCGTGGTGTGACGGGCGGTGTGTACAAGACCCGGGAACGTATTCACCGTAGCATGCTGATCTACGATTACTAGCGATTCCAGCTTCATGTAGTCGAGTTGCAGACTACAATCCGAACTGAGAACAACTTTATGGGATTTGCTTGACCTCGCGGTTTCGCTGCCCTTTGTATTGTCCATTGTAGCACGTGTGTAGCCCAAATCATAAGGGGCATGATGATTTGACGTCATCCCCACCTTCCTCCGGTTTGTCACCGGCAGTCAACTTAGAGTGCCCAACTGAATGATGGCAACTAAGTTCAAGGGTTGCGCTCGTTGCGGGACTTAACCCAACATCTCACGACACGAGCTGACGACAACCATGCACCACCTGTCACTTTGTCCTCCGAAGAGGAAAACTCTATCTCTAGAGCGGTCAAAGGATGTCAAGATTTGGTAAGGTTCTTCGCGTTGCTTCGAATTAAACCACATGCTCCACCGCTTGTGCGGGTCCCCGTCAATTCCTTTGAGTTTCAGTCTTGCGACCGTACTCCCCAGGCGGAGTGCTTAATGCGTTAGCTGCAGCACTAAGGGGCGGAAACCCCCTAACACTTAGCACTCATCGTTTACGGCGTGGACTACCAGGGTATCTAATCCTGTTTGATCCCCACGCTTTCGCACATCAGCGTCAGTTGCAGACCAGAAAGCCGCCTTCGCCACTGGTGTTCCTCCATATCTCTGCGCATTTCACCGCTACACATGGAATTCCACTTTCCTCTTCTGCACTCAAGTTTTCCAGTTTCCAATGACCCTCCACGGTTGAGCCGTGGGCTTTCACATCAGACTTAAAAAACCGCCTACGCGCGCTTTACGCCAATAATTCCGGATACGCTTGCCCCTACGATTACCGCGCTGCTGCACGTAGTAGCCGTGGTTTCTGGTAGGACCGCCAGAGGGACAGTACTAC

33

TCTGATGACCTTAGGCGGCTGGCTCCAAAAAGGTTACCCCACCGACTTCGGGTGTTACAAACTCTCGTGGTGTGACGGGCGGTGTGTACAAGGCCCGGGAACGTATTCACCGCGGCATGCTGATCCGCGATTACTAGCGATTCCAGCTTCATGTAGGCGAGTTGCAGCCTACAATCCGAACTGAGAACGGTTTTATGAGATTAGCTCCACCTCGCGGTCTTGCAGCTCTTTGTACCGTCCATTGTAGCACGTGTGTAGCCCAGGTCATAAGGGGCATGATGATTTGACGTCATCCCCACCTTCCTCCGGTTTGTCACCGGCAGTCACCTTAGAGTGCCCAACTAAATGATGGCAACTAAGATCAAGGGTTGCGCTCGTTGCGGGACTTAACCCAACATCTCACGACACGAGCTGACGACAACCATGCACCACCTGTCACTCTGCTCCCGAAGGAGAAGCCCTATCTCTAGGGTTGTCAGAGGATGTCAAGACCTGGTAAGGTTCTTCGCGTTGCTTCGAATTAAACCACATGCTCCACCGCTTGTGCGGGCCCCCGTCAATTCCTTTGAGTTTCAGCCTTGCGGCCGTACTCCCCAGGCGGAGTGCTTAATGCGTTAACTTCAGCACTAAAGGGCGGAAACCCTCTAACACTTAGCACTCATCGTTTACGGCGTGGACTACCAGGGTATCTAATCCTGTTTGCTCCCCACGCTTTCGCGCCTCAGTGTCAGTTACAGACCAGAAAGTCGCCTTCGCCACTGGTGTTCCTCCATATCTCTACGCATTTCACCGCTACACATGGAATTCCACTTTCCTCTTCTGCACTCAAGTCTCCCAGTTTCCAATGACCCTCCACGGTTGAGCCGTGGGCTTTCACATCAGACTTAAGAACCACCTGCGCGCGTTTACGCCCATAATTCCGGATAACGCTTGCCCCTACGTATTACCGCGGCTGCTGGACGTAAGTAGCCGGGCTTTCTGGTAGGACCGCAAGGGGCAGTTATTCACTAGCCTTGTCTTCCTAAAACGAGT

34

ACTGAGATCACCTTCGACGGCTCCCCCCACAAGGGTTAGGCCACCGGCTTCGGGTGTTACCGACTTTCGTGACTTGACGGGCGGTGTGTACAAGGCCCGGGAACGTATTCACCGCAGCGTTGCTGATCTGCGATTACTAGCGACTCCGACTTCATGGGGTCGAGTTGCAGACCCCAATCCGAACTGAGACCGGCTTTTTGGGATTAGCTCCACCTCACAGTATCGCAACCCATTGTACCGGCCATTGTAGCATGCGTGAAGCCCAAGACATAAGGGGCATGATGATTTGACGTCGTCCTCACCTTCCTCCGAGTTGACCCCGGCAGTCTCCCATGAGTCCCCACCATTACGTGCTGGCAACATGGAACGAGGGTTGCGCTCGTTGCGGTACTTAACCCATCATCTCACGACACGAGCTGACGACCACCATGCACCAGCTGTGAACCCGCCCCAAAGGGGAAACCGTATCTCTACGGCGATCGAGAACATGTCAAGCCTTGGTAAGGTTCTTCGCGTTGCATCGAATTAATCCGGATGCTCCGCCGGTTGTGCGGGCCCCCGTCTATTCCTTTGAGTATTATCCTTGCGGCCGTACTCCCCAGGCGGGGCACTTAATGCGTTAGCTGCGGCGCGGAAACCGTGGAATGCTCCCCACACCTAGTGCCCAACGTTTACGGCATGCACTACCAGGGTATCTAATCCTGTTCGCTCCCCATGCTTTCGCTCCTCAGCGTCAGTTACAGCACAGAGACCTGCCTTCGCCATCGGCTGTTCCATCCTGATATCTGCGCATTTCCACCGCTACACCATGTAACTTCCAGTCTCTCCTACTGCACTCTACTCTGACTTCGTACCCCAACCTCCAGAATGCCTGGGAGTTAAGGGCCGACCGGT

35

CTTCTCCCACCTTCGGCGGCTGGCTCCCTTGCGGGTTACCCCACCGACTTCGGGTGTTGTAAACTCTCGTGGTGTGACGGGCGGTGTGTACAAGACCCGGGAACGTATTCACCGCGGCATGCTGATCCGCGATTACTAGCAATTCCGACTTCATGCAGGCGAGTTGCAGCCTGCAATCCGAACTGAGACTGGCTTTTATAGGATTGGCTCCACCTCGCGGCTTCGCTTCCCGTTGTACCAGCCATTGTAGTACGTGTGTAGCCCAAGTCATAAGGGGCATGATGATTTGACGTCATCCCCGCCTTCCTCCGGTTTGTCACCGGCAGTCATTCTAGAGTGCCCAGCCTTACCTGCTGGCAACTAAAATCAAGGGTTGCGCTCGTTGCGGGACTTAACCCAACATCTCACGACACGAGCTGACGACAACCATGCACCACCTGTCTCCTCTGTCCCGAAGGCCGCTGCTATCTCTAGCAGATTCAGAGGGATGTCAAGACTTGGTAAGGTTCTTCGCGTTGCTTCGAATTAAACCACATACTCCACTGCTTGTGCGGGTCCCCGTCAATTCCTTTGAGTTTCAGTCTTGCGACCGTACTCCCCAGGCGGAATGCTTAATGTGTTAACTTCGGCACCAAGGGTATCGAAACCCCTAACACCTAGCATTCATCGTTTACGGCGTGGACTACCAGGGTATCTAATCCTGTTTGCTCCCCACGCTTTCGCGCCTCAGCGTCAGTTACAGCCCAGAGAGTCGCCTTCGCCACTGGTGTTCCTCCACATATCTACGCATTTCACCGCTACACGTGGAATTCCACTCTCCTCTTCTGCACTCAAGTCCCCCAGTTTCCAGTGCGACCTGAAGTTGAGCCCCAGGTTAAACACCAGACTTAAAGAACCGCCTGCGCGCGCTTACGCCCATAATTCCGGACACGCTTGCCCCTACGATTACCGCGCTGCTGGACGTAGTAGCCGGGGTTTCTCTCAAGACCGT

36

CTTTTGGTGACCTTCGACGGCTAGCTCCAAAATGGTTACTCCACCGGCTTCGGGTGTTACAAACTCTCGTGGTGTGACGGGCGGTGTGTACAAGACCCGGGAACGTATTCACCGTAGCATGCTGATCTACGATTACTAGCGATTCCAGCTTCATGTAGTCGAGTTGCAGACTACAATCCGAACTGAGAACAACTTTATGGGATTTGCTTGACCTCGCGGTTTCGCTGCCCTTTGTATTGTCCATTGTAGCACGTGTGTAGCCCAAATCATAAGGGGCATGATGATTTGACGTCATCCCCACCTTCCTCCGGTTTGTCACCGGCAGTCAACTTAGAGTGCCCAACTGAATGATGGCAACTAAGTTCAAGGGTTGCGCTCGTTGCGGGACTTAACCCAACATCTCACGACACGAGCTGACGACAACCATGCACCACCTGTCACTTTGTCCTCCGAAGAGGAAAACTCTATCTCTAGAGCGGTCAAAGGATGTCAAGATTTGGTAAGGTTCTTCGCGTTGCTTCGAATTAAACCACATGCTCCACCGCTTGTGCGGGTCCCCGTCAATTCCTTTGAGTTTCAGTCTTGCGACCGTACTCCCCAGGCGGAGTGCTTAATGCGTTAGCTGCAGCACTAAGGGGCGGAAACCCCCTAACACTTAGCACTCATCGTTTACGGCGTGGACTACCAGGGTATCTAATCCTGTTTGATCCCCACGCTTTCGCACATCAGCGTCAGTTGCAGACCAGAAAGCCGCCTTCGCCACTGGTGTTCCTCCATATCTCTGCGCATTTCACCGCTACACATGGAATTCCACTTTCCTCTTCTGCACTCAAGTTTTCCAGTTTCCAATGACCCTCCACGGTTGAGCCGTGGGCTTTCACATCAGACTTAAAAAACCGCCTACGCGCGCTTACGCCCATAATTCCGGATACGCTTGCCACTACGTATACCGCGCTGCTGGAACGAATTAGCCGGGGCTTTCTGGTAGGTACCGCCAAATGGG

37

GACGATCCAAGTGGTAGCGCCCTCCCGAAGGTTAAGCTACCTACTTCTTTTGCAACCCACTCCCATGGTGTGACGGGCGGTGTGTACAAGGCCCGGGAACGTATTCACCGTGGCATTCTGATCCACGATTACTAGCGATTCCGACTTCATGGAGTCGAGTTGCAGACTCCAATCCGGACTACGACGCACTTTATGAGGTCCGCTTGCTCTCGCGAGGTCGCTTCTCTTTGTATGCGCCATTGTAGCACGTGTGTAGCCCTGGTCGTAAGGGCCATGATGACTTGACGTCATCCCCACCTTCCTCCAGTTTATCACTGGCAGTCTCCTTTGAGTTCCCGGCCGGACCGCTGGCAACAAAGGATAAGGGTTGCGCTCGTTGCGGGACTTAACCCAACATTTCACAACACGAGCTGACGACAGCCATGCAGCACCTGTCTCACGGTTCCCGAAGGCACATTCTCATCTCTGAAAACTTCCGTGGATGTCAAGACCAGGTAAGGTTCTTCGCGTTGCATCGAATTAAACCACATGCTCCACCGCTTGTGCGGGCCCCCGTCAATTCATTTGAGTTTTAACCTTGCGGCCGTACTCCCCAGGCGGTCGACTTAACGCGTTAGCTCCGGAAGCCACGCCTCAAGGGCACAACCTCCAAGTCGACATCGTTTACGGCGTGGACTACCAGGGTATCTAATCCTGTTTGCTCCCCACGCTTTCGCACCTGAGCGTCAGTCTTCGTCCAGGGGGCCGCCTTCGCCACCGGTATTCCTCCAGATCTCTACGCATTTCACCGCTACACCTGGAATTCTACCCCCCTCTACGAGACTCAAGCTTGCCAGTATCAGATGCAGTTCCCAGGTTGAGCCCGGGGATTTCACATCTGACTTAACAAACCGCCTGCGTGCGCTTTACGCCCAGTAATTCCGATTAACGCTTGGACCCTCCGGATTACCGGCGGTGGTGGGACGGAGTTAGCCGGGGTTTCTCTGCGGGGAACGCCATGGGGAAAGGGTTTATCTTTTCCCCTCTCCCCCGCGAAAGATTTTA

38

GGGTATGATCCAAGTGGTAAGCGCCCTCCCGAAGGTTAAGCTACCTACTTCTTTTGCAACCCACTCCCATGGTGTGACGGGCGGTGTGTACAAGGCCCGGGAACGTATTCACCGTGGCATTCTGATCCACGATTACTAGCGATTCCGACTTCATGGAGTCGAGTTGCAGACTCCAATCCGGACTACGACGCACTTTATGAGGTCCGCTTGCTCTCGCGAGGTCGCTTCTCTTTGTATGCGCCATTGTAGCACGTGTGTAGCCCTGGTCGTAAGGGCCATGATGACTTGACGTCATCCCCACCTTCCTCCAGTTTATCACTGGCAGTCTCCTTTGAGTTCCCGGCCGGACCGCTGGCAACAAAGGATAAGGGTTGCGCTCGTTGCGGGACTTAACCCAACATTTCACAACACGAGCTGACGACAGCCATGCAGCACCTGTCTCACGGTTCCCGAAGGCACATTCTCATCTCTGAAAACTTCCGTGGATGTCAAGACCAGGTAAGGTTCTTCGCGTTGCATCGAATTAAACCACATGCTCCACCGCTTGTGCGGGCCCCCGTCAATTCATTTGAGTTTTAACCTTGCGGCCGTACTCCCCAGGCGGTCGACTTAACGCGTTAGCTCCGGAAGCCACGCCTCAAGGGCACAACCTCCAAGTCGACATCGTTTACGGCGTGGACTACCAGGGTATCTAATCCTGTTTGCTCCCCACGCTTTCGCACCTGAGCGTCAGTCTTCGTCCAGGGGGCCGCCTTCGCCACCGGTATTCCTCCAGATCTCTACGCATTTCACCGCTACACCTGGAATTCTACCCCCCTCTACGAGACTCAAGCTTGCCAGTATCAGATGCAGTTCCCAGGTTGAGCCCGGGGATTTCACATCTGACTTAACAAACCGCCTGCGTGCGCTTTACGCCCAGTAATTCCGATTAACGCTTGCACCCTCCGTATTACCGGCGCTGCTGGCACGGAGTTAGCCGGGGTTCTTCTGCGGGA

39

CCATCTTTGTGACCTTCGACGGCTAGCTCCAAAATGGTTACTCCACCGGCTTCGGGTGTTACAAACTCTCGTGGTGTGACGGGCGGTGTGTACAAGACCCGGGAACGTATTCACCGTAGCATGCTGATCTACGATTACTAGCGATTCCAGCTTCATGTAGTCGAGTTGCAGACTACAATCCGAACTGAGAACAACTTTATGGGATTTGCTTGACCTCGCGGTTTCGCTGCCCTTTGTATTGTCCATTGTAGCACGTGTGTAGCCCAAATCATAAGGGGCATGATGATTTGACGTCATCCCCACCTTCCTCCGGTTTGTCACCGGCAGTCAACTTAGAGTGCCCAACTGAATGATGGCAACTAAGTTCAAGGGTTGCGCTCGTTGCGGGACTTAACCCAACATCTCACGACACGAGCTGACGACAACCATGCACCACCTGTCACTTTGTCCTCCGAAGAGGAAAACTCTATCTCTAGAGCGGTCAAAGGATGTCAAGATTTGGTAAGGTTCTTCGCGTTGCTTCGAATTAAACCACATGCTCCACCGCTTGTGCGGGTCCCCGTCAATTCCTTTGAGTTTCAGTCTTGCGACCGTACTCCCCAGGCGGAGTGCTTAATGCGTTAGCTGCAGCACTAAGGGGCGGAAACCCCCTAACACTTAGCACTCATCGTTTACGGCGTGGACTACCAGGGTATCTAATCCTGTTTGATCCCCACGCTTTCGCACATCAGCGTCAGTTGCAGACCAGAAAGCCGCCTTCGCCACTGGTGTTCCTCCATATCTCTGCGCATTTCACCGCTACACATGGAATTCCACTTTCCTCTTCTGCACTCAAGTTTTCCAGTTTCCAATGACCCTCCACGGTTGAGCCGTGGGCTTTCACATCAGACTTAAAAAACCGCCTACGCGCGCTTTACGCCCAATAATTCCGGATAACGCTTGCCACCTACGAATTACCGC

40

ACAACTTTGTGACCTTCGACGGCTAGCTCCAAAATGGTTACTCCACCGGCTTCGGGTGTTACAAACTCTCGTGGTGTGACGGGCGGTGTGTACAAGACCCGGGAACGTATTCACCGTAGCATGCTGATCTACGATTACTAGCGATTCCAGCTTCATGTAGTCGAGTTGCAGACTACAATCCGAACTGAGAACAACTTTATGGGATTTGCTTGACCTCGCGGTTTCGCTGCCCTTTGTATTGTCCATTGTAGCACGTGTGTAGCCCAAATCATAAGGGGCATGATGATTTGACGTCATCCCCACCTTCCTCCGGTTTGTCACCGGCAGTCAACTTAGAGTGCCCAACTGAATGATGGCAACTAAGTTCAAGGGTTGCGCTCGTTGCGGGACTTAACCCAACATCTCACGACACGAGCTGACGACAACCATGCACCACCTGTCACTTTGTCCTCCGAAGAGGAAAACTCTATCTCTAGAGCGGTCAAAGGATGTCAAGATTTGGTAAGGTTCTTCGCGTTGCTTCGAATTAAACCACATGCTCCACCGCTTGTGCGGGTCCCCGTCAATTCCTTTGAGTTTCAGTCTTGCGACCGTACTCCCCAGGCGGAGTGCTTAATGCGTTAGCTGCAGCACTAAGGGGCGGAAACCCCCTAACACTTAGCACTCATCGTTTACGGCGTGGACTACCAGGGTATCTAATCCTGTTTGATCCCCACGCTTTCGCACATCAGCGTCAGTTGCAGACCAGAAAGCCGCCTTCGCCACTGGTGTTCCTCCATATCTCTGCGCATTTCACCGCTACACATGGAATTCCACTTTCCTCTTCTGCACTCAAGTTTTCCAGTTTCCAATGACCCTCCACGGTTGAGCCGTGGGCTTTCACATCAGACTTAAAAAACCGCCTACGCGCGCTTTACGCCCAATAATTCCGGATAACGCTTGCCACCTACGAATTACCGGCGCTGCTGGCACGTAGTTAGCCGTGGCTTTCTGGTTAAGT

41

ACTTTGGTGACCTTCGACGGCTAGCTCCAAAATGGTTACTCCACCGGCTTCGGGTGTTACAAACTCTCGTGGTGTGACGGGCGGTGTGTACAAGACCCGGGAACGTATTCACCGTAGCATGCTGATCTACGATTACTAGCGATTCCAGCTTCATGTAGTCGAGTTGCAGACTACAATCCGAACTGAGAACAACTTTATGGGATTTGCTTGACCTCGCGGTTTCGCTGCCCTTTGTATTGTCCATTGTAGCACGTGTGTAGCCCAAATCATAAGGGGCATGATGATTTGACGTCATCCCCACCTTCCTCCGGTTTGTCACCGGCAGTCAACTTAGAGTGCCCAACTGAATGATGGCAACTAAGTTCAAGGGTTGCGCTCGTTGCGGGACTTAACCCAACATCTCACGACACGAGCTGACGACAACCATGCACCACCTGTCACTTTGTCCTCCGAAGAGGAAAACTCTATCTCTAGAGCGGTCAAAGGATGTCAAGATTTGGTAAGGTTCTTCGCGTTGCTTCGAATTAAACCACATGCTCCACCGCTTGTGCGGGTCCCCGTCAATTCCTTTGAGTTTCAGTCTTGCGACCGTACTCCCCAGGCGGAGTGCTTAATGCGTTAGCTGCAGCACTAAGGGGCGGAAACCCCCTAACACTTAGCACTCATCGTTTACGGCGTGGACTACCAGGGTATCTAATCCTGTTTGATCCCCACGCTTTCGCACATCAGCGTCAGTTGCAGACCAGAAAGCCGCCTTCGCCACTGGTGTTCCTCCATATCTCTGCGCATTTCACCGCTACACATGGAATTCCACTTTCCTCTTCTGCACTCAAGTTTTCCAGTTTCCAATGACCCTCCACGGTTGAGCCGTGGGCTTTCACATCAGACTTAAAAAACCGCCTACGCGCGCTTTACGCCCAATAATTCCGGATAACGCTTGCCACCTACGTATTACCGCGGCTGCTGGCCCGTAATTAGCCGTGGTTTTTGGGTAGGAACCG

42

ATCTTGGTGACCTTCGACGGCTAGCTCCAAAATGGTTACTCCACCGGCTTCGGGTGTTACAAACTCTCGTGGTGTGACGGGCGGTGTGTACAAGACCCGGGAACGTATTCACCGTAGCATGCTGATCTACGATTACTAGCGATTCCAGCTTCATGTAGTCGAGTTGCAGACTACAATCCGAACTGAGAACAACTTTATGGGATTTGCTTGACCTCGCGGTTTCGCTGCCCTTTGTATTGTCCATTGTAGCACGTGTGTAGCCCAAATCATAAGGGGCATGATGATTTGACGTCATCCCCACCTTCCTCCGGTTTGTCACCGGCAGTCAACTTAGAGTGCCCAACTGAATGATGGCAACTAAGTTCAAGGGTTGCGCTCGTTGCGGGACTTAACCCAACATCTCACGACACGAGCTGACGACAACCATGCACCACCTGTCACTTTGTCCTCCGAAGAGGAAAACTCTATCTCTAGAGCGGTCAAAGGATGTCAAGATTTGGTAAGGTTCTTCGCGTTGCTTCGAATTAAACCACATGCTCCACCGCTTGTGCGGGTCCCCGTCAATTCCTTTGAGTTTCAGTCTTGCGACCGTACTCCCCAGGCGGAGTGCTTAATGCGTTAGCTGCAGCACTAAGGGGCGGAAACCCCCTAACACTTAGCACTCATCGTTTACGGCGTGGACTACCAGGGTATCTAATCCTGTTTGATCCCCACGCTTTCGCACATCAGCGTCAGTTGCAGACCAGAAAGCCGCCTTCGCCACTGGTGTTCCTCCATATCTCTGCGCATTTCACCGCTACACATGGAATTCCACTTTCCTCTTCTGCACTCAAGTTTTCCAGTTTCCAATGACCCTCCACGGTTGAGCCGTGGGCTTTCACATCAGACTTAAAAAACCGCCTACGCGCGCTTTACGCCCAATAATTCCGGATAACGCTTGCCACCTACGTATTACCGCGGCTGCTGGCACGTAGTTAGCCGTGGCTTTCTGGTTAAGTACCGCCAAAT

43

ACACTTTGTGACCTTCGACGGCTAGCTCCAAAATGGTTACTCCACCGGCTTCGGGTGTTACAAACTCTCGTGGTGTGACGGGCGGTGTGTACAAGACCCGGGAACGTATTCACCGTAGCATGCTGATCTACGATTACTAGCGATTCCAGCTTCATGTAGTCGAGTTGCAGACTACAATCCGAACTGAGAACAACTTTATGGGATTTGCTTGACCTCGCGGTTTCGCTGCCCTTTGTATTGTCCATTGTAGCACGTGTGTAGCCCAAATCATAAGGGGCATGATGATTTGACGTCATCCCCACCTTCCTCCGGTTTGTCACCGGCAGTCAACTTAGAGTGCCCAACTGAATGATGGCAACTAAGTTCAAGGGTTGCGCTCGTTGCGGGACTTAACCCAACATCTCACGACACGAGCTGACGACAACCATGCACCACCTGTCACTTTGTCCTCCGAAGAGGAAAACTCTATCTCTAGAGCGGTCAAAGGATGTCAAGATTTGGTAAGGTTCTTCGCGTTGCTTCGAATTAAACCACATGCTCCACCGCTTGTGCGGGTCCCCGTCAATTCCTTTGAGTTTCAGTCTTGCGACCGTACTCCCCAGGCGGAGTGCTTAATGCGTTAGCTGCAGCACTAAGGGGCGGAAACCCCCTAACACTTAGCACTCATCGTTTACGGCGTGGACTACCAGGGTATCTAATCCTGTTTGATCCCCACGCTTTCGCACATCAGCGTCAGTTGCAGACCAGAAAGCCGCCTTCGCCACTGGTGTTCCTCCATATCTCTGCGCATTTCACCGCTACACATGGAATTCCACTTTCCTCTTCTGCACTCAAGTTTTCCAGTTTCCAATGACCCTCCACGGTTGAGCCGTGGGCTTTCACATCAGACTTAAAAAACCGCCTACGCGCGCTTTACGCCCAATAATTCCGGATAACGCTTGCCACCTACGTATTACCGCGGCTGCTGGCCCGAAGTTACCCGGGGTTTTTGGGTAGGACCC

44

TGACGATCCAAGTGGTAGCGCCCTCCCGAAGGTTAAGCTACCTACTTCTTTTGCAACCCACTCCCATGGTGTGACGGGCGGTGTGTACAAGGCCCGGGAACGTATTCACCGTGGCATTCTGATCCACGATTACTAGCGATTCCGACTTCATGGAGTCGAGTTGCAGACTCCAATCCGGACTACGACGCACTTTATGAGGTCCGCTTGCTCTCGCGAGGTCGCTTCTCTTTGTATGCGCCATTGTAGCACGTGTGTAGCCCTGGTCGTAAGGGCCATGATGACTTGACGTCATCCCCACCTTCCTCCAGTTTATCACTGGCAGTCTCCTTTGAGTTCCCGGCCGGACCGCTGGCAACAAAGGATAAGGGTTGCGCTCGTTGCGGGACTTAACCCAACATTTCACAACACGAGCTGACGACAGCCATGCAGCACCTGTCTCACGGTTCCCGAAGGCACATTCTCATCTCTGAAAACTTCCGTGGATGTCAAGACCAGGTAAGGTTCTTCGCGTTGCATCGAATTAAACCACATGCTCCACCGCTTGTGCGGGCCCCCGTCAATTCATTTGAGTTTTAACCTTGCGGCCGTACTCCCCAGGCGGTCGACTTAACGCGTTAGCTCCGGAAGCCACGCCTCAAGGGCACAACCTCCAAGTCGACATCGTTTACGGCGTGGACTACCAGGGTATCTAATCCTGTTTGCTCCCCACGCTTTCGCACCTGAGCGTCAGTCTTCGTCCAGGGGGCCGCCTTCGCCACCGGTATTCCTCCAGATCTCTACGCATTTCACCGCTACACCTGGAATTCTACCCCCCTCTACGAGACTCAAGCTTGCCAGTATCAGATGCAGTTCCCAGGTTGAGCCCGGGGATTTCACATCTGACTTAACAAACCGCCTGCGTGCGCTTTACGCCCAGTAATTCCGATTAACGCTTGCACCCTCCGGATTACCGCGGCTGGTGGCACGGAGTTAGCCGGGGTTCTTCTGCGGGTAAC

45

TCAACTCTGTGACCTTAGGCGGCTGGCTCCAAAAAGGTTACCCCACCGACTTCGGGTGTTACAAACTCTCGTGGTGTGACGGGCGGTGTGTACAAGGCCCGGGAACGTATTCACCGCGGCATGCTGATCCGCGATTACTAGCGATTCCAGCTTCATGTAGGCGAGTTGCAGCCTACAATCCGAACTGAGAACGGTTTTATGAGATTAGCTCCACCTCGCGGTCTTGCAGCTCTTTGTACCGTCCATTGTAGCACGTGTGTAGCCCAGGTCATAAGGGGCATGATGATTTGACGTCATCCCCACCTTCCTCCGGTTTGTCACCGGCAGTCACCTTAGAGTGCCCAACTTAATGATGGCAACTAAGATCAAGGGTTGCGCTCGTTGCGGGACTTAACCCAACATCTCACGACACGAGCTGACGACAACCATGCACCACCTGTCACTCTGCTCCCGAAGGAGAAGCCCTATCTCTAGGGTTTTCAGAGGATGTCAAGACCTGGTAAGGTTCTTCGCGTTGCTTCGAATTAAACCACATGCTCCACCGCTTGTGCGGGCCCCCGTCAATTCCTTTGAGTTTCAGCCTTGCGGCCGTACTCCCCAGGCGGAGTGCTTAATGCGTTAACTTCAGCACTAAAGGGCGGAAACCCTCTAACACTTAGCACTCATCGTTTACGGCGTGGACTACCAGGGTATCTAATCCTGTTTGCTCCCCACGCTTTCGCGCCTCAGTGTCAGTTACAGACCAGAAAGTCGCCTTCGCCACTGGTGTTCCTCCATATCTCTACGCATTTCACCGCTACACATGGAATTCCACTTTCCTCTTCTGCACTCAAGTCTCCCAGTTTCCAATGACCCTCCACGGTTGAGCCGTGGGCTTTCACATCAGACTTAAGAAACCACCTGCGCGCGCTTTACGCCCAATAATTCCGGATAACGCTTGCCACCTACGAATTACCGCGGCTGCTGGCACGTAGTTAGCCGGGGTTTCCGGGTAGGGA

46

ACAACTTTGTGAGCTTCGACGGCTAGTTCCTAAAAGGTTACTCCACCGGCTTCGGGTGTTACAAACTCTCGTGGTGTGACGGGCGGTGTGTACAAGACCCGGGAACGTATTCACCGTAGCATGCTGATCTACGATTACTAGCGATTCCAGCTTCATGTAGTCGAGTTGCAGACTACAATCCGAACTGAGAACGGCTTTATGGGATTTGCTTGACCTCGCGGGTTCGCTGCCCTTTGTACCGTCCATTGTAGCACGTGTGTAGCCCAAATCATAAGGGGCATGATGATTTGACGTCATCCCCACCTTCCTCCGGTTTGTCACCGGCAGTCAACTTAGAGTGCCCAACTTAATGCTGGCAACTAAGCTTAAGGGTTGCGCTCGTTGCGGGACTTAACCCAACATCTCACGACACGAGCTGACGACAACCATGCACCACCTGTCACTTTGTCCCCCGAAGGGGAAGGCTCTATCTCTAGAGTTGTCAAAGGATGTCAAGATTTGGTAAGGTTCTTCGCGTTGCTTCGAATTAAACCACATGCTCCACCGCTTGTGCGGGTCCCCGTCAATTCCTTTGAGTTTCAGCCTTGCGGCCGTACTCCCCAGGCGGAGTGCTTAATGCGTTAGCTGCAGCACTAAGGGGCGGAAACCCCCTAACACTTAGCACTCATCGTTTACGGCGTGGACTACCAGGGTATCTAATCCTGTTTGATCCCCACGCTTTCGCACATCAGCGTCAGTTGCAGACCAGAAAGTCGCCTTCGCCACTGGTGTTCCTCCATATCTCTGCGCATTTCACCGCTACACATGGAATTCCACTTTCCTCTTCTGCACTCAAGTTTTCCAGTTTCCAATGACCCTCCACGGTTGAGCCGTGGGCTTTCACATCAGACTTAAAAAACCGCCTACGCGCGCTTTACGCCCATAATTCCGGATAACGCTTGCACCTACGATTACCGCGGCTGCTGGCCCGAAGTAGCCGGGGTTTCTGATAAGGACCGCCAGGGAGGCAAGTACTTACTCCTTGGCTTCCCAAAAACAAGTTTACAA

47

CTTCTTCTTTTACCTTAGGCGGCTGGCTCCTAATAGGTTACCTCACCGACTTCGGGTGTTACAAACTCTCGTGGTGTGACGGGCGGTGTGTACAAGGCCCGGGAACGTATTCACCGCGGCGTGCTGATCCGCGATTACTAGCGATTCCGACTTCATGTAGGCGAGTTGCAGCCTACAATCCGAACTGAGATTGGCTTTCAGAGATTAGCTTGCCGTCACCGGCTTGCGACTCGTTGTACCAACCATTGTAGCACGTGTGTAGCCCAGGTCATAAGGGGCATGATGATTTGACGTCATCCCCACCTTCCTCCGGTTTATTACCGGCAGTCTCGCTAGAGTGCCCAACTTAATGATGGCAACTAACAATAGGGGTTGCGCTCGTTGCGGGACTTAACCCAACATCTCACGACACGAGCTGACGACAACCATGCACCACCTGTCACCGATGTACCGAAGTAAAACTCTATCTCTAGAGCGGGCATCGGGATGTCAAGACCTGGTAAGGTTCTTCGCGTTGCTTCGAATTAAACCACATGCTCCACCGCTTGTGCGGGCCCCCGTCAATTCCTTTGAGTTTCAACCTTGCGGTCGTACTCCCCAGGCGGAGTGCTTAATGCGTTAGCTCCGGCACTAAGCCCCGGAAAGGGCCTAACACCTAGCACTCATCGTTTACGGCGTGGACTACCAGGGTATCTAATCCTGTTTGCTCCCCACGCTTTCGAGCCTCAGCGTCAGTTACAGACCAGAGAGCCGCTTTCGCCACCGGTGTTCCTCCATATATCTACGCATTTCACCGCTACACATGGAATTCCACTCTCCCCTTCTGCACTCAAGTTCTCCAGTTTCCAAAGCATACATTGGTTGAGCCAATGCCTTTAACTTCAGACTTAAAGAACCGCCTGCGCTCGCTTTACGCCCATAAATCCGGACACGCTCGGGACTACGTTTACCGCGCTGCTGGACGTAGTAGCCGCCCTTTCTGTTAGTACCGCCACTAATGGGTTTCACTCCCTTACCGTCTTCCTAAAACAG

48

TTCTCGGGAACCGTGGTACCGCCCTCTTTGCAGTTAGGCTAGCTACTTCTGGTGCAACAAACTCCCATGGTGTGACGGGCGGTGTGTACAAGGCCCGGGAACGTATTCACCGCGGCATTCTGATCCGCGATTACTAGCGATTCCGACTTCATGGAGTCGAGTTGCAGACTCCAATCCGGACTACGATCGGCTTTTTGAGATTAGCATCACATCGCTGTGTAGCAACCCTTTGTACCGACCATTGTAGCACGTGTGTAGCCCTGGCCGTAAGGGCCATGATGACTTGACGTCGTCCCCGCCTTCCTCCAGTTTGTCACTGGCAGTATCCTTAAAGTTCCCATCCGAAATGCTGGCAAGTAAGGAAAAGGGTTGCGCTCGTTGCGGGACTTAACCCAACATCTCACGACACGAGCTGACGACAGCCATGCAGCACCTGTATCTAGATTCCCGAAGGCACCAATCCATCTCTGGAAAGTTTCTAGTATGTCAAGGCCAGGTAAGGTTCTTCGCGTTGCATCGAATTAAACCACATGCTCCACCGCTTGTGCGGGCCCCCGTCAATTCATTTGAGTTTTAGTCTTGCGACCGTACTCCCCAGGCGGTCTACTTATCGCGTTAGCTGCGCCACTAAAGCCTCAAAGGCCCCAACGGCTAGTAGACATCGTTTACGGCATGGACTACCAGGGTATCTAATCCTGTTTGCTCCCCATGCTTTCGTACCTCAGCGTCAGTATTAGGCCAGATGGCTGCCTTCGCCATCGGTATTCCTCCAGATCTCTACGCATTTCACCGCTACACCTGGAATTCTACCATCCTCTCCCATACTCTAGCTCACCAGTATCGAATGCAATTCCCAAGTTAAGCTCGGGGATTTCACATCCGACTTAATAAGCCGCCTACGCACGCTTTACGCCCAGTAAATCCGATTAACGCTCGCAACCTCTGTATTACCGCGGCTGCTGGCACAGAGTTAGCCGGGGTTATTCTGCGAGAACGTCACTATCTCAAGGATTAACTAAAGAACCTCCTCTCCGTTAAAGGGTTTACACCATAAGGCTT

49

TAATCGGAACCGTGGTACCGCCCTCTTTGCAGTTAGGCTAGCTACTTCTGGTGCAACAAACTCCCATGGTGTGACGGGCGGTGTGTACAAGGCCCGGGAACGTATTCACCGCGGCATTCTGATCCGCGATTACTAGCGATTCCGACTTCATGGAGTCGAGTTGCAGACTCCAATCCGGACTACGATCGGCTTTTTGAGATTAGCATCACATCGCTGTGTAGCAACCCTTTGTACCGACCATTGTAGCACGTGTGTAGCCCTGGCCGTAAGGGCCATGATGACTTGACGTCGTCCCCGCCTTCCTCCAGTTTGTCACTGGCAGTATCCTTAAAGTTCCCATCCGAAATGCTGGCAAGTAAGGAAAAGGGTTGCGCTCGTTGCGGGACTTAACCCAACATCTCACGACACGAGCTGACGACAGCCATGCAGCACCTGTATCTAGATTCCCGAAGGCACCAATCCATCTCTGGAAAGTTTCTAGTATGTCAAGGCCAGGTAAGGTTCTTCGCGTTGCATCGAATTAAACCACATGCTCCACCGCTTGTGCGGGCCCCCGTCAATTCATTTGAGTTTTAGTCTTGCGACCGTACTCCCCAGGCGGTCTACTTATCGCGTTAGCTGCGCCACTAAAGCCTCAAAGGCCCCAACGGCTAGTAGACATCGTTTACGGCATGGACTACCAGGGTATCTAATCCTGTTTGCTCCCCATGCTTTCGTACCTCAGCGTCAGTATTAGGCCAGATGGCTGCCTTCGCCATCGGTATTCCTCCAGATCTCTACGCATTTCACCGCTACACCTGGAATTCTACCATCCTCTCCCATACTCTAGCTCACCAGTATCGAATGCAATTCCCAAGTTAAGCTCGGGGATTTCACATCCGACTTAATAAGCCGCCTACGCACGCTTTACGCCCAGTAAATCCGATTAACGCTCGCACCCTCTGGATTACCGCGGCTGCTGGCACAGAGTTAGCCGGGGTTATTCTGCGAGAACGCCACTAT

50

CCCACTCTGTTAGCTTCGGCGGCTGGCTCCAAAAGGTTACCTCACCGACTTCGGGTGTTACAAACTCTCGTGGTGTGACGGGCGGTGTGTACAAGGCCCGGGAACGTATTCACCGCGGCATGCTGATCCGCGATTACTAGCGATTCCAGCTTCACGCAGTCGAGTTGCAGACTGCGATCCGAACTGAGAACAGATTTGTGGGATTGGCTTAGCCTCGCGGCTTCGCTGCCCTTTGTTCTGCCCATTGTAGCACGTGTGTAGCCCAGGTCATAAGGGGCATGATGATTTGACGTCATCCCCACCTTCCTCCGGTTTGTCACCGGCAGTCACCTTAGAGTGCCCAACTGAATGCTGGCAACTAAGATCAAGGGTTGCGCTCGTTGCGGGACTTAACCCAACATCTCACGACACGAGCTGACGACAACCATGCACCACCTGTCACTCTGCCCCCGAAGGGGAAGCCCTATCTCTAGGGTTGTCAGAGGATGTCAAGACCTGGTAAGGTTCTTCGCGTTGCTTCGAATTAAACCACATGCTCCACCGCTTGTGCGGGCCCCCGTCAATTCCTTTGAGTTTCAGTCTTGCGACCGTACTCCCCAGGCGGAGTGCTTAATGCGTTTGCTGCAGCACTAAAGGGCGGAAACCCTCTAACACTTAGCACTCATCGTTTACGGCGTGGACTACCAGGGTATCTAATCCTGTTCGCTCCCCACGCTTTCGCGCCTCAGCGTCAGTTACAGACCAGAGAGTCGCCTTCGCCACTGGTGTTCCTCCACATCTCTACGCATTTCACCGCTACACGTGGAATTCCACTCTCCTCTTCTGCACTCAAGTTCCCCAGTTTCCAATGACCCTCCCCGGTTGAGCCGGGGGCTTTCACATCAGACTTAAGAAACCGCCTGCGCGCGCTTTACGCCCAATAATTCCGGACAACGCTTGCCACCTACGTATTACCGCGGCTGCTGGCACGTAGTTAGCCGGGGTTTCCGGGTTAGGACCG

51

TCGAATCCTCCGTGGTACCGTCCCCCTTGCGGTTAGACTAGCTACTTCTGGAGCAACCCACTCCCATGGTGTGACGGGCGGTGTGTACAAGGCCCGGGAACGTATTCACCGTGACATTCTGATTCACGATTACTAGCGATTCCGACTTCACGCAGTCGAGTTGCAGACTGCGATCCGGACTACGATCGGTTTTATGGGATTAGCTCCACCTCGCGGCTTGGCAACCCTTTGTACCGACCATTGTAGCACGTGTGTAGCCCTGGCCGTAAGGGCCATGATGACTTGACGTCATCCCCACCTTCCTCCGGTTTGTCACCGGCAGTCTCCTTAGAGTGCCCACCCGAGGTGCTGGTAACTAAGGACAAGGGTTGCGCTCGTTACGGGACTTAACCCAACATCTCACGACACGAGCTGACGACAGCCATGCAGCACCTGTGTCTGAGTTCCCGAAGGCACCAATCCATCTCTGGAAAGTTCTCAGCATGTCAAGGCCAGGTAAGGTTCTTCGCGTTGCTTCGAATTAAACCACATGCTCCACCGCTTGTGCGGGCCCCCGTCAATTCATTTGAGTTTTAACCTTGCGGCCGTACTCCCCAGGCGGTCGACTTATCGCGTTAGCTGCGCCACTAAGATCTCAAGGATCCCAACGGCTAGTCGACATCGTTTACGGCGTGGACTACCAGGGTATCTAATCCTGTTTGCTCCCCACGCTTTCGCACCTCAGTGTCAGTATCAGTCCAGGTGGTCGCCTTCGCCACTGGTGTTCCTTCCTATATCTACGCATTTCACCGCTACACAGGAAATTCCACCACCCTCTACCGTACTCTAGCTCAGTAGTTTTGGATGCAGTTCCCAGGTTGAGCCCGGGGATTTCACATCCAACTTGCTGAACCACCTACGCGCGCTTTACGCCCAGTAATTCCGATTAACGCTTGCACCCTTCGTATTACCGCGGCTGCTGGCACGAAGTTAGCCGGGGCTTATTCTGGTTGGAACGTCAAACCAGCAAGGTATTAACTTACTGCCCTTCCTCCCACTTAAAGGGTTTACATCCGAGAA

52

ATCTTTGTGACCTTCGACGGCTAGCTCCAAAATGGTTACTCCACCGGCTTCGGGTGTTACAAACTCTCGTGGTGTGACGGGCGGTGTGTACAAGACCCGGGAACGTATTCACCGTAGCATGCTGATCTACGATTACTAGCGATTCCAGCTTCATGTAGTCGAGTTGCAGACTACAATCCGAACTGAGAACAACTTTATGGGATTTGCTTGACCTCGCGGTTTCGCTGCCCTTTGTATTGTCCATTGTAGCACGTGTGTAGCCCAAATCATAAGGGGCATGATGATTTGACGTCATCCCCACCTTCCTCCGGTTTGTCACCGGCAGTCAACTTAGAGTGCCCAACTGAATGATGGCAACTAAGTTCAAGGGTTGCGCTCGTTGCGGGACTTAACCCAACATCTCACGACACGAGCTGACGACAACCATGCACCACCTGTCACTTTGTCCTCCGAAGAGGAAAACTCTATCTCTAGAGCGGTCAAAGGATGTCAAGATTTGGTAAGGTTCTTCGCGTTGCTTCGAATTAAACCACATGCTCCACCGCTTGTGCGGGTCCCCGTCAATTCCTTTGAGTTTCAGTCTTGCGACCGTACTCCCCAGGCGGAGTGCTTAATGCGTTAGCTGCAGCACTAAGGGGCGGAAACCCCCTAACACTTAGCACTCATCGTTTACGGCGTGGACTACCAGGGTATCTAATCCTGTTTGATCCCCACGCTTTCGCACATCAGCGTCAGTTGCAGACCAGAAAGCCGCCTTCGCCACTGGTGTTCCTCCATATCTCTGCGCATTTCACCGCTACACATGGAATTCCACTTTCCTCTTCTGCACTCAAGTTTTCCAGTTTCCAATGACCCTCCACGGTTGAGCCGTGGGCTTTCCCATCAGACTTAAAAAACCGCCTACGCGCGCTTTACGCCCAATAATTCCGGATAACGCTTGCACCTACGTATTACCGGGCTGCTGGCACGTAGTTAGCCGGGGTTTCTGGTTAGTACCG

53

AACTGGCGTTCCCTTCGACGGCTCCCCCCACAAGGGTTAGGCCACCGGCTTCGGGTGTTACCAACTTTCGTGACTTGACGGGCGGTGTGTACAAGGCCCGGGAACGTATTCACCGCAGCGTTGCTGATCTGCGATTACTAGCGACTCCGACTTCATGGGGTCGAGTTGCAGACCCCAATCCGAACTGAGACCGGCTTTTTGGGATTAGCTCCACCTCACAGTATCGCAACCCTTTGTACCGGCCATTGTAGCATGCGTGAAGCCCAAGACATAAGGGGCATGATGATTTGACGTCGTCCCCACCTTCCTCCGAGTTGACCCCGGCAGTCTCCTATGAGTCCCCGCCATAACGCGCTGGCAACATAGAACGAGGGTTGCGCTCGTTGCGGGACTTAACCCAACATCTCACGACACGAGCTGACGACAACCATGCACCACCTGTAAACCGACCGCAAGCGGGGCACCTGTTTCCAGGTCTTTCCGGTTCATGTCAAGCCTTGGTAAGGTTCTTCGCGTTGCATCGAATTAATCCGCATGCTCCGCCGCTTGTGCGGGCCCCCGTCAATTCCTTTGAGTTTTAGCCTTGCGGCCGTACTCCCCAGGCGGGGCACTTAATGCGTTAGCTACGGCGCGGAAAACGTGGAATGTCCCCCACACCTAGTGCCCAACGTTTACGGCATGGACTACCAGGGTATCTAATCCTGTTCGCTCCCCATGCTTTCGCTCCTCAGCGTCAGTTACAGCCCAGAGACCTGCCTTCGCCATCGGTGTTCCTCCTGATATCTGCGCATTTCACCGCTACACCAGGAATTCCAGTCTCCCCTACTGCACTCTAGTCTGCCCGTACCCACTGCAGAACCGGAGTTGAGCCCCGGTCTTTCACAGCAGACGCGACAAACCGCCTACGAGCTCTTTACGCCCAATAATTCCGGATAACGCTTGCGCCCTACGTATTACCGCGGCTGCTGGCACGTAGTTAGCCGGGGTTTTTCTGGAGGACCG

54

CCCCCTTTGTGACCTTCGACGGCTAGCTCCAAATGGTTACTCCACCGGCTTCGGGTGTTACAAACTCTCGTGGTGTGACGGGCGGTGTGTACAAGACCCGGGAACGTATTCACCGTAGCATGCTGATCTACGATTACTAGCGATTCCAGCTTCATGTAGTCGAGTTGCAGACTACAATCCGAACTGAGAACAACTTTATGGGATTTGCTTGACCTCGCGGTTTCGCTGCCCTTTGTATTGTCCATTGTAGCACGTGTGTAGCCCAAATCATAAGGGGCATGATGATTTGACGTCATCCCCACCTTCCTCCGGTTTGTCACCGGCAGTCAACTTAGAGTGCCCAACTGAATGATGGCAACTAAGTTCAAGGGTTGCGCTCGTTGCGGGACTTAACCCAACATCTCACGACACGAGCTGACGACAACCATGCACCACCTGTCACTTTGTCCTCCGAAGAGGAAAACTCTATCTCTAGAGCGGTCAAAGGATGTCAAGATTTGGTAAGGTTCTTCGCGTTGCTTCGAATTAAACCACATGCTCCACCGCTTGTGCGGGTCCCCGTCAATTCCTTTGAGTTTCAGTCTTGCGACCGTACTCCCCAGGCGGAGTGCTTAATGCGTTAGCTGCAGCACTAAGGGGCGGAAACCCCCTAACACTTAGCACTCATCGTTTACGGCGTGGACTACCAGGGTATCTAATCCTGTTTGATCCCCACGCTTTCGCACATCAGCGTCAGTTGCAGACCAGAAAGCCGCCTTCGCCACTGGTGTTCCTCCATATCTCTGCGCATTTCACCGCTACACATGGAATTCCACTTTCCTCTTCTGCACTCAAGTTTTCCAGTTTCCAATGACCCTCCACGGTTGAGCCGTGGGCTTTCACATCAGACTTAAAAAACCGCCTACGCGCGCTTTACGCCCAAAATTCCGGATAACGCTTGCACCTACGATTACCGGGGTGGTGGCC

55

AACTTTTGTGACCTTCGACGGCTAGCTCCAAAATGGTTACTCCACCGGCTTCGGGTGTTACAAACTCTCGTGGTGTGACGGGCGGTGTGTACAAGACCCGGGAACGTATTCACCGTAGCATGCTGATCTACGATTACTAGCGATTCCAGCTTCATGTAGTCGAGTTGCAGACTACAATCCGAACTGAGAACAACTTTATGGGATTTGCTTGACCTCGCGGTTTCGCTGCCCTTTGTATTGTCCATTGTAGCACGTGTGTAGCCCAAATCATAAGGGGCATGATGATTTGACGTCATCCCCACCTTCCTCCGGTTTGTCACCGGCAGTCAACTTAGAGTGCCCAACTGAATGATGGCAACTAAGTTCAAGGGTTGCGCTCGTTGCGGGACTTAACCCAACATCTCACGACACGAGCTGACGACAACCATGCACCACCTGTCACTTTGTCCTCCGAAGAGGAAAACTCTATCTCTAGAGCGGTCAAAGGATGTCAAGATTTGGTAAGGTTCTTCGCGTTGCTTCGAATTAAACCACATGCTCCACCGCTTGTGCGGGTCCCCGTCAATTCCTTTGAGTTTCAGTCTTGCGACCGTACTCCCCAGGCGGAGTGCTTAATGCGTTAGCTGCAGCACTAAGGGGCGGAAACCCCCTAACACTTAGCACTCATCGTTTACGGCGTGGACTACCAGGGTATCTAATCCTGTTTGATCCCCACGCTTTCGCACATCAGCGTCAGTTGCAGACCAGAAAGCCGCCTTCGCCACTGGTGTTCCTCCATATCTCTGCGCATTTCACCGCTACACATGGAATTCCACTTTCCTCTTCTGCACTCAAGTTTTCCAGTTTCCAATGACCCTCCACGGTTGAGCCGTGGGCTTTCACATCAGACTTAAAAAACCGCCTACGCGCGTTTACGCCCATAATTCCGGATAACGCTTGCACCTACGATTACCGCGCTGCTGGA

57

TATCGGTACCGTGGTACCGCCCTCTTTGCAGTTAGGCTAGCTACTTCTGGTGCAACAAACTCCCATGGTGTGACGGGCGGTGTGTACAAGGCCCGGGAACGTATTCACCGCGGCATTCTGATCCGCGATTACTAGCGATTCCGACTTCATGGAGTCGAGTTGCAGACTCCAATCCGGACTACGATCGGCTTTTTGAGATTAGCATCCTATCGCTAGGTAGCAACCCTTTGTACCGACCATTGTAGCACGTGTGTAGCCCTGGCCGTAAGGGCCATGATGACTTGACGTCGTCCCCGCCTTCCTCCAGTTTGTCACTGGCAGTATCCTTAAAGTTCCCGACATTACTCGCTGGCAAATAAGGAAAAGGGTTGCGCTCGTTGCGGGACTTAACCCAACATCTCACGACACGAGCTGACGACAGCCATGCAGCACCTGTATGTAAGTTCCCGAAGGCACCAATCCATCTCTGGAAAGTTCTTACTATGTCAAGGCCAGGTAAGGTTCTTCGCGTTGCATCGAATTAAACCACATGCTCCACCGCTTGTGCGGGCCCCCGTCAATTCATTTGAGTTTTAGTCTTGCGACCGTACTCCCCAGGCGGTCTACTTATCGCGTTAGCTGCGCCACTAAAGCCTCAAAGGCCCCAACGGCTAGTAGACATCGTTTACGGCATGGACTACCAGGGTATCTAATCCTGTTTGCTCCCCATGCTTTCGCACCTCAGCGTCAGTGTTAGGCCAGATGGCTGCCTTCGCCATCGGTATTCCTCCAGATCTCTACGCATTTCACCGCTACACCTGGAATTCTACCATCCTCTCCCACACTCTAGCTAACCAGTATCGAATGCAATTCCCAAGTTAAGCTCGGGGATTTCACATTTGACTTAATTAGCCGCCTACGCGCGCTTTACGCCCAGTAAATCCGATTAACGCTTGCACCCTCTGGATTACCGCGGCTGCTGGCAC

58

ACATTTCAATTACCTTAGGCGGCTGGCTCCAAAAAGGTTACCTCACCGACTTCGGGTGTTACAAACTCTCGTGGTGTGACGGGCGGTGTGTACAAGGCCCGGGAACGTATTCACCGCGGCGTGCTGATCCGCGATTACTAGCGATTCCGGCTTCATGCAGGCGAGTTGCAGCCTGCAATCCGAACTGAGAGAAGCTTTAAGAGATTTGCATGACCTCGCGGTCTAGCGACTCGTTGTACTTCCCATTGTAGCACGTGTGTAGCCCAGGTCATAAGGGGCATGATGATTTGACGTCATCCCCACCTTCCTCCGGTTTGTCACCGGCAGTCTCGCTAGAGTGCCCAACTAAATGATGGCAACTAACAATAAGGGTTGCGCTCGTTGCGGGACTTAACCCAACATCTCACGACACGAGCTGACGACAACCATGCACCACCTGTCACTTTGTCCCCGAAGGGAAAGCTCTATCTCTAGAGTGGTCAAAGGATGTCAAGACCTGGTAAGGTTCTTCGCGTTGCTTCGAATTAAACCACATGCTCCACCGCTTGTGCGGGCCCCCGTCAATTCCTTTGAGTTTCAACCTTGCGGTCGTACTCCCCAGGCGGAGTGCTTAATGCGTTTGCTGCAGCACTGAAGGGCGGAAACCCTCCAACACTTAGCACTCATCGTTTACGGCGTGGACTACCAGGGTATCTAATCCTGTTTGCTCCCCACGCTTTCGAGCCTCAGCGTCAGTTACAGACCAGAGAGCCGCCTTCGCCACTGGTGTTCCTCCATATATCTACGCATTTCACCGCTACACATGGAATTCCACTCTCCTCTTCTGCACTCAAGTCTCCCAGTTTCCAATGACCCTCCCCGGTTGAGCCGGGGGCTTTCACATCAGACTTAAGAAACCGCCTGCGCTCGCTTTACGCCCATAAATCCGGACAACGCTTGCCACCTACGTATTACCGCGGCTGCTGGCACGTAGTTAGCCGGGGTTTCTGGTAAATACCG

59

CACTTTGTGACCTTCGACGGCTAGCTCCAAAATGGTTACTCCACCGGCTTCGGGTGTTACAAACTCTCGTGGTGTGACGGGCGGTGTGTACAAGACCCGGGAACGTATTCACCGTAGCATGCTGATCTACGATTACTAGCGATTCCAGCTTCATGTAGTCGAGTTGCAGACTACAATCCGAACTGAGAACAACTTTATGGGATTTGCTTGACCTCGCGGTTTCGCTGCCCTTTGTATTGTCCATTGTAGCACGTGTGTAGCCCAAATCATAAGGGGCATGATGATTTGACGTCATCCCCACCTTCCTCCGGTTTGTCACCGGCAGTCAACTTAGAGTGCCCAACTGAATGATGGCAACTAAGTTCAAGGGTTGCGCTCGTTGCGGGACTTAACCCAACATCTCACGACACGAGCTGACGACAACCATGCACCACCTGTCACTTTGTCCTCCGAAGAGGAAAACTCTATCTCTAGAGCGGTCAAAGGATGTCAAGATTTGGTAAGGTTCTTCGCGTTGCTTCGAATTAAACCACATGCTCCACCGCTTGTGCGGGTCCCCGTCAATTCCTTTGAGTTTCAGTCTTGCGACCGTACTCCCCAGGCGGAGTGCTTAATGCGTTAGCTGCAGCACTAAGGGGCGGAAACCCCCTAACACTTAGCACTCATCGTTTACGGCGTGGACTACCAGGGTATCTAATCCTGTTTGATCCCCACGCTTTCGCACATCAGCGTCAGTTGCAGACCAGAAAGCCGCCTTCGCCACTGGTGTTCCTCCATATCTCTGCGCATTTCACCGCTACACATGGAATTCCACTTTCCTCTTCTGCACTCAAGTTTTCCAGTTTCCAATGACCCTCCACGGTTGAGCCGTGGGCTTTCACATCAGACTTAAAAAACCGCCTACGCGCGCTTTACGCCCAATAATTCCGGATAACGCTTGCCACCTACGAATTACCGCGGCTGCTGGCCCGTAATTAACCGGG

60

CACTTTGTGAGCTTCGACGGCTAGCTCCAAAATGGTTACTCCACCGGCTTCGGGTGTTACAAACTCTCGTGGTGTGACGGGCGGTGTGTACAAGACCCGGGAACGTATTCACCGTAGCATGCTGATCTACGATTACTAGCGATTCCAGCTTCATGTAGTCGAGTTGCAGACTACAATCCGAACTGAGAACAACTTTATGGGATTTGCTTGACCTCGCGGTTTCGCTGCCCTTTGTATTGTCCATTGTAGCACGTGTGTAGCCCAAATCATAAGGGGCATGATGATTTGACGTCATCCCCACCTTCCTCCGGTTTGTCACCGGCAGTCAACTTAGAGTGCCCAACTGAATGATGGCAACTAAGTTCAAGGGTTGCGCTCGTTGCGGGACTTAACCCAACATCTCACGACACGAGCTGACGACAACCATGCACCACCTGTCACTTTGTCCTCCGAAGAGGAAAACTCTATCTCTAGAGCGGTCAAAGGATGTCAAGATTTGGTAAGGTTCTTCGCGTTGCTTCGAATTAAACCACATGCTCCACCGCTTGTGCGGGTCCCCGTCAATTCCTTTGAGTTTCAGTCTTGCGACCGTACTCCCCAGGCGGAGTGCTTAATGCGTTAGCTGCAGCACTAAGGGGCGGAAACCCCCTAACACTTAGCACTCATCGTTTACGGCGTGGACTACCAGGGTATCTAATCCTGTTTGATCCCCACGCTTTCGCACATCAGCGTCAGTTGCAGACCAGAAAGCCGCCTTCGCCACTGGTGTTCCTCCATATCTCTGCGCATTTCACCGCTACACATGGAATTCCACTTTCCTCTTCTGCACTCAAGTTTTCCAGTTTCAATGACCCTCCACGGTTGAGCCGTGGGCTTTCACATCAGACTTAAAAAACCGCCTACGCGCGCTTTACGCCCAATAATTCCGGATAACGCTTGGCCCCTA

61

ATATTTGTACACCTTCGACGGCTAGCTCCAAAATGGTTACTCCACCGGCTTCGGGTGTTACAAACTCTCGTGGGGTGACGGGCGGGGTGTACAAGACCCGGGAACGTATTCACCGTAGCATGCTGATCTACGATTACTAGCGATTCCAGCTTCATGTAGTCGAGTTGCAGACTACAATCCGAACTGAGAACAACTTTATGGGATTTGCTTGACCTCGCGGTTTCGCTGCCCTTTGTATTGTCCATTGTAGCACGTGTGTAGCCCAAATCATAAGGGGCATGATGATTTGACGTCATCCCCACCTTCCTCCGGTTTGTCACCGGCAGTCAACTTAGAGTGCCCAACTGAATGATGGCAACTAAGTTCAAGGGTTGCGCTCGTTGCGGGACTTAACCCAACATCTCACGACACGAGCTGACGACAACCATGCACCACCTGTCACTTTGTCCTCCGAAGAGGAAAACTCTATCTCTAGAGCGGTCAAAGGATGTCAAGATTTGGTAAGGTTCTTCGCGTTGCTTCGAATTAAACCACATGCTCCACCGCTTGTGCGGGTCCCCGTCAATTCCTTTGAGTTTCAGTCTTGCGACCGTACTCCCCAGGCGGAGTGCTTAATGCGTTAGCTGCAGCACTAAGGGGCGGAAACCCCCTAACACTTAGCACTCATCGTTTACGGCGTGGACTACCAGGGTATCTAATCCTGTTTGATCCCCACGCTTTCGCACATCAGCGTCAGTTGCAGACCAGAAAGCCGCCTTCGCCACTGGTGTTCCTCCATATCTCTGCGCATTTCACCGCTACACATGGAATTCCACTTTCCTCTTCTGCACTCAAGTTTTCCAGTTTCCAATGACCCTCCACGGTTGAGCCGTGGGCTTTCCCATCAGACTTAAAAAACCGCCTACGCGCGTTTACGCCCATAATTCCGGATAACGCTTGCCCCTACGATTACCGCGCTGCTGCACGAATTAGCCGGGGTTTCTGTTAGGACCGC

62

AACTTTGTGACCTTCGACGGCTAGCTCCAGATGGTTACTCCACCGGCTTCGGGTGTTACAAACTCTCGTGGTGTGACGGGCGGTGTGTACAAGACCCGGGAACGTATTCACCGTAGCATGCTGATCTACGATTACTAGCGATTCCAGCTTCATGTAGTCGAGTTGCAGACTACAATCCGAACTGAGAACAACTTTATGGGATTTGCTTGACCTCGCGGTTTCGCTGCCCTTTGTATTGTCCATTGTAGCACGTGTGTAGCCCAAATCATAAGGGGCATGATGATTTGACGTCATCCCCACCTTCCTCCGGTTTGTCACCGGCAGTCAACTTAGAGTGCCCAACTGAATGATGGCAACTAAGTTCAAGGGTTGCGCTCGTTGCGGGACTTAACCCAACATCTCACGACACGAGCTGACGACAACCATGCACCACCTGTCACTTTGTCCTCCGAAGAGGAAAACTCTATCTCTAGAGCGGTCAAAGGATGTCAAGATTTGGTAAGGTTCTTCGCGTTGCTTCGAATTAAACCACATGCTCCACCGCTTGTGCGGGTCCCCGTCAATTCCTTTGAGTTTCAGTCTTGCGACCGTACTCCCCAGGCGGAGTGCTTAATGCGTTAGCTGCAGCACTAAGGGGCGGAAACCCCCTAACACTTAGCACTCATCGTTTACGGCGTGGACTACCAGGGTATCTAATCCTGTTTGATCCCCACGCTTTCGCACATCAGCGTCAGTTGCAGACCAGAAAGCCGCCTTCGCCACTGGTGTTCCTCCATATCTCTGCGCATTTCACCGCTACACATGGAATTCCACTTTCCTCTTCTGCACTCAAGTTTTCCAGTTTCCAATGACCCTCCACGGTTGAGCCGTGGGCTTTCACATCAGACTTAAAAAACCGCCTACGCGGGCTTTACGCCCAATAATTCCGGATAACGCTTGCCACCTA

63

CCACTTTGTGACCTTCGACGGCTAGCTCCAGATGGTTACTCCACCGGCTTCGGGTGTTACAAACTCTCGTGGTGTGACGGGCGGTGTGTACAAGACCCGGGAACGTATTCACCGTAGCATGCTGATCTACGATTACTAGCGATTCCAGCTTCATGTAGTCGAGTTGCAGACTACAATCCGAACTGAGAACAACTTTATGGGATTTGCTTGACCTCGCGGTTTCGCTGCCCTTTGTATTGTCCATTGTAGCACGTGTGTAGCCCAAATCATAAGGGGCATGATGATTTGACGTCATCCCCACCTTCCTCCGGTTTGTCACCGGCAGTCAACTTAGAGTGCCCAACTGAATGATGGCAACTAAGTTCAAGGGTTGCGCTCGTTGCGGGACTTAACCCAACATCTCACGACACGAGCTGACGACAACCATGCACCACCTGTCACTTTGTCCTCCGAAGAGGAAAACTCTATCTCTAGAGCGGTCAAAGGATGTCAAGATTTGGTAAGGTTCTTCGCGTTGCTTCGAATTAAACCACATGCTCCACCGCTTGTGCGGGTCCCCGTCAATTCCTTTGAGTTTCAGTCTTGCGACCGTACTCCCCAGGCGGAGTGCTTAATGCGTTAGCTGCAGCACTAAGGGGCGGAAACCCCCTAACACTTAGCACTCATCGTTTACGGCGTGGACTACCAGGGTATCTAATCCTGTTTGATCCCCACGCTTTCGCACATCAGCGTCAGTTGCAGACCAGAAAGCCGCCTTCGCCACTGGTGTTCCTCCATATCTCTGCGCATTTCACCGCTACACATGGAATTCCACTTTCCTCTTCTGCACTCAAGTTTTCCAGTTTCCAATGACCCTCCACGGTTGAGCCGTGGGCTTTCACATCAGACTTAAAAAACCGCCTACGCGCGCTTTACGCCCAATAATTCCGGATAACGCTTGCCACCTACGTATTACCCGCGCTG

64

CTTACGATACAAGTGGTAAGCGCCCTCCCGAAGGTTAAGCTACCTACTTCTTTTGCAACCCACTCCCATGGTGTGACGGGCGGTGTGTACAAGGCCCGGGAACGTATTCACCGTAGCATTCTGATCTACGATTACTAGCGATTCCGACTTCATGGAGTCGAGTTGCAGACTCCAATCCGGACTACGACATACTTTATGAGGTCCGCTTGCTCTCGCGAGGTCGCTTCTCTTTGTATATGCCATTGTAGCACGTGTGTAGCCCTGGTCGTAAGGGCCATGATGACTTGACGTCATCCCCACCTTCCTCCAGTTTATCACTGGCAGTCTCCTTTGAGTTCCCGGCCTAACCGCTGGCAACAAAGGATAAGGGTTGCGCTCGTTGCGGGACTTAACCCAACATTTCACAACACGAGCTGACGACAGCCATGCAGCACCTGTCTCACAGTTCCCGAAGGCACCAATCCATCTCTGGAAAGTTCTGTGGATGTCAAGACCAGGTAAGGTTCTTCGCGTTGCATCGAATTAAACCACATGCTCCACCGCTTGTGCGGGCCCCCGTCAATTCATTTGAGTTTTAACCTTGCGGCCGTACTCCCCAGGCGGTCGATTTAACGCGTTAGCTCCGGAAGCCACGCCTCAAGGGCACAACCTCCAAATCGACATCGTTTACGGCGTGGACTACCAGGGTATCTAATCCTGTTTGCTCCCCACGCTTTCGCACCTGAGCGTCAGTCTTTGTCCAGGGGGCCGCCTTCGCCACCGGTATTCCTCCAGATCTCTACGCATTTCACCGCTACACCTGGAATTCTACCCCCCTCTACAAGACTCTAGCCTGCCAGTTTCGAATGCAGTTCCCAGGTTGAGCCCGGGGATTTCACATCCGACTTGACAGACCGCCTGCGTGCGCTTTACGCCCAGTAATTCCGATTAACGCTTGCACCCTCCGGATTACCGCGGGTGGTGGGACGGAGTTAACCGGGGTTCTTCTGGGGGAACGCCA

65

GGGTGATACAAGTGGTAAGCGCCCTCCCGAAGGTTAAGCTACCTACTTCTTTTGCAACCCACTCCCATGGTGTGACGGGCGGTGTGTACAAGGCCCGGGAACGTATTCACCGTGGCATTCTGATCCACGATTACTAGCGATTCCGACTTCATGGAGTCGAGTTGCAGACTCCAATCCGGACTACGACGCACTTTATGAGGTCCGCTTGCTCTCGCGAGGTCGCTTCTCTTTGTATGCGCCATTGTAGCACGTGTGTAGCCCTGGTCGTAAGGGCCATGATGACTTGACGTCATCCCCACCTTCCTCCAGTTTATCACTGGCAGTCTCCTTTGAGTTCCCGGCCGGACCGCTGGCAACAAAGGATAAGGGTTGCGCTCGTTGCGGGACTTAACCCAACATTTCACAACACGAGCTGACGACAGCCATGCAGCACCTGTCTCACGGTTCCCGAAGGCACATTCTCATCTCTGAAAACTTCCGTGGATGTCAAGACCAGGTAAGGTTCTTCGCGTTGCATCGAATTAAACCACATGCTCCACCGCTTGTGCGGGCCCCCGTCAATTCATTTGAGTTTTAACCTTGCGGCCGTACTCCCCAGGCGGTCGACTTAACGCGTTAGCTCCGGAAGCCACGCCTCAAGGGCACAACCTCCAAGTCGACATCGTTTACGGCGTGGACTACCAGGGTATCTAATCCTGTTTGCTCCCCACGCTTTCGCACCTGAGCGTCAGTCTTCGTCCAGGGGGCCGCCTTCGCCACCGGTATTCCTCCAGATCTCTACGCATTTCACCGCTACACCTGGAATTCTACCCCCCTCTACGAGACTCAAGCTTGCCAGTATCAGATGCAGTTCCCAGGTTGAGCCCGGGGATTTCACATCTGACTTAACAAACCGCCTGCGTGCGTTTACGCCCAGTAATTCCGATAACGCTTGCCCCTCCGATTACCGCGGTGCTGGCACGGAGTTAGCCGGGGTTCTTCTGCGGG

66

TCTTTTGGTGACCTTCGACGGCTAGCTCCAAAATGGTTACTCCACCGGCTTCGGGTGTTACAAACTCTCGTGGTGTGACGGGCGGTGTGTACAAGACCCGGGAACGTATTCACCGTAGCATGCTGATCTACGATTACTAGCGATTCCAGCTTCATGTAGTCGAGTTGCAGACTACAATCCGAACTGAGAACAACTTTATGGGATTTGCTTGACCTCGCGGTTTCGCTGCCCTTTGTATTGTCCATTGTAGCACGTGTGTAGCCCAAATCATAAGGGGCATGATGATTTGACGTCATCCCCACCTTCCTCCGGTTTGTCACCGGCAGTCAACTTAGAGTGCCCAACTGAATGATGGCAACTAAGTTCAAGGGTTGCGCTCGTTGCGGGACTTAACCCAACATCTCACGACACGAGCTGACGACAACCATGCACCACCTGTCACTTTGTCCTCCGAAGAGGAAAACTCTATCTCTAGAGCGGTCAAAGGATGTCAAGATTTGGTAAGGTTCTTCGCGTTGCTTCGAATTAAACCACATGCTCCACCGCTTGTGCGGGTCCCCGTCAATTCCTTTGAGTTTCAGTCTTGCGACCGTACTCCCCAGGCGGAGTGCTTAATGCGTTAGCTGCAGCACTAAGGGGCGGAAACCCCCTAACACTTAGCACTCATCGTTTACGGCGTGGACTACCAGGGTATCTAATCCTGTTTGATCCCCACGCTTTCGCACATCAGCGTCAGTTGCAGACCAGAAAGCCGCCTTCGCCACTGGTGTTCCTCCATATCTCTGCGCATTTCACCGCTACACATGGAATTCCACTTTCCTCTTCTGCACTCAAGTTTTCCAGTTTCCAATGACCCTCCACGGTTGAGCCGTGGGCTTTCACATCAGACTTAAAAAACCGCCTACGCGCGCTTTACGCCCAATAATTCCGGATAACGCTTGCCACCTACGTATTACCGGCGCTGCTGGCACGT

67

CTCTCTGTGACCTTAGGCGGCTGGCTCCAAAAAGGTTACCCCACCGACTTCGGGTGTTACAAACTCTCGTGGTGTGACGGGCGGTGTGTACAAGGCCCGGGAACGTATTCACCGCGGCATGCTGATCCGCGATTACTAGCGATTCCAGCTTCATGTAGGCGAGTTGCAGCCTACAATCCGAACTGAGAACGGTTTTATGAGATTAGCTCCACCTCGCGGTCTTGCAGCTCTTTGTACCGTCCATTGTAGCACGTGTGTAGCCCAGGTCATAAGGGGCATGATGATTTGACGTCATCCCCACCTTCCTCCGGTTTGTCACCGGCAGTCACCTTAGAGTGCCCAACTTAATGATGGCAACTAAGATCAAGGGTTGCGCTCGTTGCGGGACTTAACCCAACATCTCACGACACGAGCTGACGACAACCATGCACCACCTGTCACTCTGCTCCCGAAGGAGAAGCCCTATCTCTAGGGTTTTCAGAGGATGTCAAGACCTGGTAAGGTTCTTCGCGTTGCTTCGAATTAAACCACATGCTCCACCGCTTGTGCGGGCCCCCGTCAATTCCTTTGAGTTTCAGCCTTGCGGCCGTACTCCCCAGGCGGAGTGCTTAATGCGTTAACTTCAGCACTAAAGGGCGGAAACCCTCTAACACTTAGCACTCATCGTTTACGGCGTGGACTACCAGGGTATCTAATCCTGTTTGCTCCCCACGCTTTCGCGCCTCAGTGTCAGTTACAGACCAGAAAGTCGCCTTCGCCACTGGTGTTCCTCCATATCTCTACGCATTTCACCGCTACACATGGAATTCCACTTTCCTCTTCTGCACTCAAGTCTCCCAGTTTCCAATGACCCTCCACGGTTGAGCCGTGGGCTTTCACATCAGACTTAAGAAACCACCTGCGCGCGCTTACGCCCATAATTCCGGATAACGCTTGCACCTACGATTACCGCGCTGCTGGCA

68

ATTTTTGTCACCTTCGACGGCTAGCTCCAAAATGGTTACTCCACCGGCTTCGGGTGTTACAAACTCTCGTGGTGTGACGGGCGGTGTGTACAAGACCCGGGAACGTATTCACCGTAGCATGCTGATCTACGATTACTAGCGATTCCAGCTTCATGTAGTCGAGTTGCAGACTACAATCCGAACTGAGAACAACTTTATGGGATTTGCTTGACCTCGCGGTTTCGCTGCCCTTTGTATTGTCCATTGTAGCACGTGTGTAGCCCAAATCATAAGGGGCATGATGATTTGACGTCATCCCCACCTTCCTCCGGTTTGTCACCGGCAGTCAACTTAGAGTGCCCAACTGAATGATGGCAACTAAGTTCAAGGGTTGCGCTCGTTGCGGGACTTAACCCAACATCTCACGACACGAGCTGACGACAACCATGCACCACCTGTCACTTTGTCCTCCGAAGAGGAAAACTCTATCTCTAGAGCGGTCAAAGGATGTCAAGATTTGGTAAGGTTCTTCGCGTTGCTTCGAATTAAACCACATGCTCCACCGCTTGTGCGGGTCCCCGTCAATTCCTTTGAGTTTCAGTCTTGCGACCGTACTCCCCAGGCGGAGTGCTTAATGCGTTAGCTGCAGCACTAAGGGGCGGAAACCCCCTAACACTTAGCACTCATCGTTTACGGCGTGGACTACCAGGGTATCTAATCCTGTTTGATCCCCACGCTTTCGCACATCAGCGTCAGTTGCAGACCAGAAAGCCGCCTTCGCCACTGGTGTTCCTCCATATCTCTGCGCATTTCACCGCTACACATGGAATTCCACTTTCCTCTTCTGCACTCAAGTTTTCCAGTTTCCAATGACCCTCCACGGTTGAGCCGTGGGCTTTCACATCAGACTTAAAAAACCGCCTACGCGCGCTTTACGCCCATAATTCCGGATAACGCTTGCCCCTACGTATACCGCGCTGCTGGCACGAATTAGCCGGGGTTTCTGGTTAGTACCG

69

TTTTGGTTACCTTCGACGGCTAGCTCCAAATGGTTACTCCACCGGCTTCGGGTGTTACAAACTCTCGTGGTGTGACGGGCGGTGTGTACAAGACCCGGGAACGTATTCACCGTAGCATGCTGATCTACGATTACTAGCGATTCCAGCTTCATGTAGTCGAGTTGCAGACTACAATCCGAACTGAGAACAACTTTATGGGATTTGCTTGACCTCGCGGTTTCGCTGCCCTTTGTATTGTCCATTGTAGCACGTGTGTAGCCCAAATCATAAGGGGCATGATGATTTGACGTCATCCCCACCTTCCTCCGGTTTGTCACCGGCAGTCAACTTAGAGTGCCCAACTGAATGATGGCAACTAAGTTCAAGGGTTGCGCTCGTTGCGGGACTTAACCCAACATCTCACGACACGAGCTGACGACAACCATGCACCACCTGTCACTTTGTCCTCCGAAGAGGAAAACTCTATCTCTAGAGCGGTCAAAGGATGTCAAGATTTGGTAAGGTTCTTCGCGTTGCTTCGAATTAAACCACATGCTCCACCGCTTGTGCGGGTCCCCGTCAATTCCTTTGAGTTTCAGTCTTGCGACCGTACTCCCCAGGCGGAGTGCTTAATGCGTTAGCTGCAGCACTAAGGGGCGGAAACCCCCTAACACTTAGCACTCATCGTTTACGGCGTGGACTACCAGGGTATCTAATCCTGTTTGATCCCCACGCTTTCGCACATCAGCGTCAGTTGCAGACCAGAAAGCCGCCTTCGCCACTGGTGTTCCTCCATATCTCTGCGCATTTCACCGCTACACATGGAATTCCACTTTCCTCTTCTGCACTCAAGTTTTCCAGTTTCCAATGACCCTCCACGGTTGAGCCGTGGGCTTTCACATCAGACTTAAAAAACCGCCTACGCGCGCTTTACGCCCATAATTCCGGATAACGCTTGCCACCTACGAATTACCGCGGCTGCTGGCACGTAAGTAGCCGGGGCTTTCTGGTTAGGGACCGTCAAG

70

CCCTTGTCCCCTTCGACGGCTAGCTCCAAAATGGTTACTCCACCGGCTTCGGGTGTTACAAACTCTCGTGGTGTGACGGGCGGTGTGTACAAGACCCGGGAACGTATTCACCGTAGCATGCTGATCTACGATTACTAGCGATTCCAGCTTCATGTAGTCGAGTTGCAGACTACAATCCGAACTGAGAACAACTTTATGGGATTTGCTTGACCTCGCGGTTTCGCTGCCCTTTGTATTGTCCATTGTAGCACGTGTGTAGCCCAAATCATAAGGGGCATGATGATTTGACGTCATCCCCACCTTCCTCCGGTTTGTCACCGGCAGTCAACTTAGAGTGCCCAACTGAATGATGGCAACTAAGTTCAAGGGTTGCGCTCGTTGCGGGACTTAACCCAACATCTCACGACACGAGCTGACGACAACCATGCACCACCTGTCACTTTGTCCTCCGAAGAGGAAAACTCTATCTCTAGAGCGGTCAAAGGATGTCAAGATTTGGTAAGGTTCTTCGCGTTGCTTCGAATTAAACCACATGCTCCACCGCTTGTGCGGGTCCCCGTCAATTCCTTTGAGTTTCAGTCTTGCGACCGTACTCCCCAGGCGGAGTGCTTAATGCGTTAGCTGCAGCACTAAGGGGCGGAAACCCCCTAACACTTAGCACTCATCGTTTACGGCGTGGACTACCAGGGTATCTAATCCTGTTTGATCCCCACGCTTTCGCACATCAGCGTCAGTTGCAGACCAGAAAGCCGCCTTCGCCACTGGTGTTCCTCCATATCTCTGCGCATTTCACCGCTACACATGGAATTCCACTTTCCTCTTCTGCACTCAAGTTTTCCAGTTTCCAATGACCCTCCACGGTTGAGCCGTGGGCTTTCACATCAGACTTAAAAAACCGCCTACGCGCGCTTTACGCCCAATAATTCCGGAAAACGCTTGCCCCCAA

71

CTCTGGGGGACCGTGGTAGCGTCCTCCTTACGGTTAGACTACCTACTTCTGGTGCAACAAACTCCCATGGTGTGACGGGCGGTGTGTACAAGGCCCGGGAACGTATTCACCGCGGCATTCTGATCCGCGATTACTAGCGATTCCGACTTCATGGAGTCGAGTTGCAGACTCCAATCCGGACTACGATCGGCTTTTTGAGATTAGCATCCTATCGCTAGGTAGCAACCCTTTGTACCGACCATTGTAGCACGTGTGTAGCCCTGGTCGTAAGGGCCATGATGACTTGACGTCGTCCCCGCCTTCCTCCAGTTTGTCACTGGCAGTATCCTTAAAGTTCCCGGCTTAACCCGCTGGCAAATAAGGAAAAGGGTTGCGCTCGTTGCGGGACTTAACCCAACATCTCACGACACGAGCTGACGACAGCCATGCAGCACCTGTATGTAAGTTCCCGAAGGCACCAATCCATCTCTGGAAAGTTCTTACTATGTCAAGACCAGGTAAGGTTCTTCGCGTTGCATCGAATTAAACCACATGCTCCACCGCTTGTGCGGGCCCCCGTCAATTCATTTGAGTTTTAGTCTTGCGACCGTACTCCCCAGGCGGTCTACTTATCGCGTTAGCTGCGCCACTAAAGCCTCAAAGGCCCCAACGGCTAGTAGACATCGTTTACGGCATGGACTACCAGGGTATCTAATCCTGTTTGCTCCCCATGCTTTCGTACCTCAGCGTCAGTATTAGGCCAGATGGCTGCCTTCGCCATCGGTATTCCTCCAGATCTCTACGCATTTCACCGCTACACCTGGAATTCTACCATCCTCTCCCATACTCTAGCTGACCAGTATCGAATGCAATTCCTAAGTTAAGCTCAGGGATTTCACATCCGACTTAATCAGCCGCCTACGCACGCTTTACGCCCAGTAAATCCGATTAACGCTCGCCCCTCTGGATTACCGGGGTGGTGGGCCA

72

TTATTCCAAGTGGTAAGCGCCCTCCCGAAGGTTAAGCTACCTACTTCTTTTGCAACCCACTCCCATGGTGTGACGGGCGGTGTGTACAAGGCCCGGGAACGTATTCACCGTAGCATTCTGATCTACGATTACTAGCGATTCCGACTTCATGGAGTCGAGTTGCAGACTCCAATCCGGACTACGACAGACTTTATGAGTTCCGCTTGCTCTCGCGAGGTCGCTTCTCTTTGTATCTGCCATTGTAGCACGTGTGTAGCCCTACTCGTAAGGGCCATGATGACTTGACGTCATCCCCACCTTCCTCCGGTTTATCACCGGCAGTCTCCTTTGAGTTCCCGCCATCACGCGCTGGCAACAAAGGATAAGGGTTGCGCTCGTTGCGGGACTTAACCCAACATTTCACAACACGAGCTGACGACAGCCATGCAGCACCTGTCTCAGAGTTCCCGAAGGCACTCCTCTATCTCTAAAGGATTCTCTGGATGTCAAGAGTAGGTAAGGTTCTTCGCGTTGCATCGAATTAAACCACATGCTCCACCGCTTGTGCGGGCCCCCGTCAATTCATTTGAGTTTTAACCTTGCGGCCGTACTCCCCAGGCGGTCGATTTAACGCGTTAGCTCCAGAAGCCACGGTTCAAGACCACAACCTCTAAATCGACATCGTTTACAGCGTGGACTACCAGGGTATCTAATCCTGTTTGCTCCCCACGCTTTCGCACCTGAGCGTCAGTCTTTGTCCAGGGGGCCGCCTTCGCCACCGGTATTCCTCCACATCTCTACGCATTTCACCGCTACACGTGGAATTCTACCCCCCTCTACAAGACTCTAGCCAACCAGTTTCAGATGCAATTCCCAAGTTAAGCTCGGGGCTTTCACATCTGACTTAATTGACCGCCTGCGTGCGCTTTACGCCCAGTAATTCCGATTAACGGTTGGACCCTCCGGATTACCGCGGTGGTGGGACGGA

73

TTTTTTCCGATCCAAGTGGTAGCGCCCTCCCGAAGGTTAAGCTACCTACTTCTTTTGCAACCCACTCCCATGGTGTGACGGGCGGTGTGTACAAGGCCCGGGAACGTATTCACCGTAGCATTCTGATCTACGATTACTAGCGATTCCGACTTCATGGAGTCGAGTTGCAGACTCCAATCCGGACTACGACAGACTTTATGAGTTCCGCTTGCTCTCGCGAGGTCGCTTCTCTTTGTATCTGCCATTGTAGCACGTGTGTAGCCCTACTCGTAAGGGCCATGATGACTTGACGTCATCCCCACCTTCCTCCGGTTTATCACCGGCAGTCTCCTTTGAGTTCCCGCCATCACGCGCTGGCAACAAAGGATAAGGGTTGCGCTCGTTGCGGGACTTAACCCAACATTTCACAACACGAGCTGACGACAGCCATGCAGCACCTGTCTCAGAGTTCCCGAAGGCACTCCTCTATCTCTAAAGGATTCTCTGGATGTCAAGAGTAGGTAAGGTTCTTCGCGTTGCATCGAATTAAACCACATGCTCCACCGCTTGTGCGGGCCCCCGTCAATTCATTTGAGTTTTAACCTTGCGGCCGTACTCCCCAGGCGGTCGATTTAACGCGTTAGCTCCAGAAGCCACGGTTCAAGACCACAACCTCTAAATCGACATCGTTTACAGCGTGGACTACCAGGGTATCTAATCCTGTTTGCTCCCCACGCTTTCGCACCTGAGCGTCAGTCTTTGTCCAGGGGGCCGCCTTCGCCACCGGTATTCCTCCACATCTCTACGCATTTCACCGCTACACGTGGAATTCTACCCCCCTCTACAAGACTCTAGCCAACCAGTTTCAGATGCAATTCCCAAGTTAAGCTCGGGGCTTTCACATCTGACTTAATTGACCGCCTGCGTGCGCTTTACGCCCAGTAATTCCGATTAACGCTTGCACCCTCCGTATTACCGCGGCTGCTGGCACGGAGTTAGCCGGGGTTCTTCTGCGGGAACGTCATTGCTAAAAG

74

CCGGGATACAAGTGGTAGCGCCCTCCCGAAGGTTAAGCTACCTACTTCTTTTGCAACCCACTCCCATGGTGTGACGGGCGGTGTGTACAAGGCCCGGGAACGTATTCACCGTGGCATTCTGATCCACGATTACTAGCGATTCCGACTTCATGGAGTCGAGTTGCAGACTCCAATCCGGACTACGACGCACTTTATGAGGTCCGCTTGCTCTCGCGAGGTCGCTTCTCTTTGTATGCGCCATTGTAGCACGTGTGTAGCCCTGGTCGTAAGGGCCATGATGACTTGACGTCATCCCCACCTTCCTCCAGTTTATCACTGGCAGTCTCCTTTGAGTTCCCGGCCGGACCGCTGGCAACAAAGGATAAGGGTTGCGCTCGTTGCGGGACTTAACCCAACATTTCACAACACGAGCTGACGACAGCCATGCAGCACCTGTCTCACGGTTCCCGAAGGCACATTCTCATCTCTGAAAACTTCCGTGGATGTCAAGACCAGGTAAGGTTCTTCGCGTTGCATCGAATTAAACCACATGCTCCACCGCTTGTGCGGGCCCCCGTCAATTCATTTGAGTTTTAACCTTGCGGCCGTACTCCCCAGGCGGTCGACTTAACGCGTTAGCTCCGGAAGCCACGCCTCAAGGGCACAACCTCCAAGTCGACATCGTTTACGGCGTGGACTACCAGGGTATCTAATCCTGTTTGCTCCCCACGCTTTCGCACCTGAGCGTCAGTCTTCGTCCAGGGGGCCGCCTTCGCCACCGGTATTCCTCCAGATCTCTACGCATTTCACCGCTACACCTGGAATTCTACCCCCCTCTACGAGACTCAAGCTTGCCAGTATCAGATGCAGTTCCCAGGTTGAGCCCGGGGATTTCACATCTGACTTAACAAACCGCCTGCGTGCGCTTTACGCCCAGTAATTCCGATTAACGCTTGGACCCTCCGGATTACCGCGGTGGTGGGACGGAGTTAACCGG

75

GAATCCAAGTGGTAAGCGCCCTCCCGAAGGTTAAGCTACCTACTTCTTTTGCAACCCACTCCCATGGTGTGACGGGCGGTGTGTACAAGGCCCGGGAACGTATTCACCGTGGCATTCTGATCCACGATTACTAGCGATTCCGACTTCATGGAGTCGAGTTGCAGACTCCAATCCGGACTACGACGCACTTTATGAGGTCCGCTTGCTCTCGCGAGGTCGCTTCTCTTTGTATGCGCCATTGTAGCACGTGTGTAGCCCTGGTCGTAAGGGCCATGATGACTTGACGTCATCCCCACCTTCCTCCAGTTTATCACTGGCAGTCTCCTTTGAGTTCCCGGCCGGACCGCTGGCAACAAAGGATAAGGGTTGCGCTCGTTGCGGGACTTAACCCAACATTTCACAACACGAGCTGACGACAGCCATGCAGCACCTGTCTCACGGTTCCCGAAGGCACATTCTCATCTCTGAAAACTTCCGTGGATGTCAAGACCAGGTAAGGTTCTTCGCGTTGCATCGAATTAAACCACATGCTCCACCGCTTGTGCGGGCCCCCGTCAATTCATTTGAGTTTTAACCTTGCGGCCGTACTCCCCAGGCGGTCGACTTAACGCGTTAGCTCCGGAAGCCACGCCTCAAGGGCACAACCTCCAAGTCGACATCGTTTACGGCGTGGACTACCAGGGTATCTAATCCTGTTTGCTCCCCACGCTTTCGCACCTGAGCGTCAGTCTTCGTCCAGGGGGCCGCCTTCGCCACCGGTATTCCTCCAGATCTCTACGCATTTCACCGCTACACCTGGAATTCTACCCCCCTCTACGAGACTCAAGCTTGCCAGTATCAGATGCAGTTCCCAGGTTGAGCCCGGGGATTTCACATCTGACTTAACAAACCGCCTGCGTGCGCTTTACGCCCAGTAATTCCGATTAACGCTTGCACCCTCCGGATTACCGCGGCTGCTGGCACGGAGTTAACCGGGGTTTCTCTGCGGGAA

76

ATTGGATACACCTTGGGACGGCTCCCTCCAGAAGGGTAACCTACCTACTTCTTTTGGACCCACTCCCATGGGGTGACGGGCGGTGTGTACAAGGCCCGGGAACGTATTCACCGGGGCATTCTGATCCACGATTACTAACGATTCCGACTTCATGGAGTCGAGTTGCAGACTCCAATCCGGACTAGAACGCACTTTATGAGGATTGCTTGCTCTCGCGAGGTCGCTTCTCTTTGTATGCGCCATTGGAGCACGGGGGGAACCCTGGTCGTAAGGGGCATGATGAATTGACGTCATCCCCACCTTCCTCCAGGTTATCACTGGGAGGCTCCTTTGAATTCCCGACCGGACCGATGGCAACAAAGGATAAGGGTTGCGCTCGTTGCGGGACTTAACCCAACATTTCACAACACGAGCTGACGACAGCCATGCACCACCTGTCTCACAGTTCCCGAAGGAACAATCTCATCTCTGAAAAGTTCTGTGGATGTCAAGACCTGGTAAGGTTCTTCGCGTTGCATCGAATTAAACCACATGCTCCACCGCTTGTGCGGGCCCCCGTCAATTCATTTGAGTTTTAACCTTGCGGCCGTACTCCCCAGGCGGACGACTTAACGCGTTAGCTCCGGAAGTCAAGGCTCGAGAGCACCCACCTACATATCGATCTCGTTTACGGCGTGGGACTACCAGGGTATCTAATACTGGTTGCTCCCCCCGCTTTCGCAGCTGAGCGGCAGTCGTACGGACAGGAGGGCGCCGTCGGCCACTGGTAGTTCCTCCAGATCTTCTACCGCATTCAACGGCTACACCTGGAAATCTCACCTCCTCATCTAGGACTCAGCTTGGCAGATTCCATGAGACATCCTAGTTGATCCGGTGAT

77

CCGGGAATACAAAGTGGTAGCGCCCTCCCGAAGGTTAAGCTACCTACTTCTTTTGCAACCCACTCCCATGGTGTGACGGGCGGTGTGTACAAGGCCCGGGAACGTATTCACCGTGGCATTCTGATCCACGATTACTAGCGATTCCGACTTCATGGAGTCGAGTTGCAGACTCCAATCCGGACTACGACGCACTTTATGAGGTCCGCTTGCTCTCGCGAGGTCGCTTCTCTTTGTATGCGCCATTGTAGCACGTGTGTAGCCCTGGTCGTAAGGGCCATGATGACTTGACGTCATCCCCACCTTCCTCCAGTTTATCACTGGCAGTCTCCTTTGAGTTCCCGGCCGGACCGCTGGCAACAAAGGATAAGGGTTGCGCTCGTTGCGGGACTTAACCCAACATTTCACAACACGAGCTGACGACAGCCATGCAGCACCTGTCTCACGGTTCCCGAAGGCACATTCTCATCTCTGAAAACTTCCGTGGATGTCAAGACCAGGTAAGGTTCTTCGCGTTGCATCGAATTAAACCACATGCTCCACCGCTTGTGCGGGCCCCCGTCAATTCATTTGAGTTTTAACCTTGCGGCCGTACTCCCCAGGCGGTCGACTTAACGCGTTAGCTCCGGAAGCCACGCCTCAAGGGCACAACCTCCAAGTCGACATCGTTTACGGCGTGGACTACCAGGGTATCTAATCCTGTTTGCTCCCCACGCTTTCGCACCTGAGCGTCAGTCTTCGTCCAGGGGGCCGCCTTCGCCACCGGTATTCCTCCAGATCTCTACGCATTTCACCGCTACACCTGGAATTCTACCCCCCTCTACGAGACTCAAGCTTGCCAGTATCAGATGCAGTTCCCAGGTTGAGCCCGGGGATTTCACATCTGACTTAACAAACCGCCTGCGTGCGCTTTACGCCCAGTAATTCCGATTAACGCTTGCACCCTCCGTATTACCGCGGCTGCTGGCACGGAGTTAGCCGGGGCTTCTTCTGCGGGTAACGTCATGGAGCAAGGTTTAACTTTACCCCTTCCTCCCGGTGAAGGACTTTACACCCGAAGGCTTCTCTTACCG

78

CTCCTGTGACCTTCGGCGGCTGGCTCCATAAAGGTTACCTCACCGACTTCGGGTGTTACAAACTCTCGTGGTGTGACGGGCGGTGTGTACAAGACCCGGGAACGTATTCACCGTGGCGTGCTGATCCACGATTACTAGCGATTCCGGCTTCATGCAGGCGAGTTGCAGCCTGCAATCCGAACTGAGAATGGCTTTAAGAGATTTGCTTGCCCTCGCGGGTTTGCGACTCGTTGTACCATCCATTGTAGCACGTGTGTAGCCCAAGTCATAAGGGGCATGCTGATTTGACGTCATCCCCACCTTCCTCCGGTTTGTCACCGGCAGTCTCATTAGAGTGCCCAACTAAATGCTGGCAACTAATAATAGGGGTTGCGCTCGTTGCGGGACTTAACCCAACATCTCACGACACGAGCTGACGACAACCATGCACCACCTGTCACTTTGTCCCCGAAGGGAAAGCCCTATCTCTAGGGTGGTCAAAGGATGTCAAGACTTGGTAAGGTTCTTCGCGTTGCTTCGAATTAAACCACATGCTCCACCGCTTGTGCGGGTCCCCGTCAATTCCTTTGAGTTTCAACCTTGCGGTCGTACTCCCCAGGCGGAGTGCTTAATGCGTTAACTGCGGCACTGAAGGGCGGAAACCCTCCAACACCTAGCACTCATCGTTTACGGCGTGGACTACCAGGGTATCTAATCCTGTTTGCTCCCCACGCTTTCGAGCCTCAGTGTCAGTAACAGACCAGAATGTCGCCTTCGCCACTGGTGTTCTTCCATATATCTACGCATTCCACCGCTACACATGGAGTTCCACATTCCTCTTCTGTACTCAAGTTTCCCAGTTTCCAATGACCCTCCACGGTTAAGCCGTGGGCTTTCACATCAGACTTAAGAAACCACCTGCGCTCCCTTTACGCCCATAAATCCGGACAACGCTTGCCCCTACGTATTACCGCGGCTGCTGGACCGAATTAACCGGGGTTTCTGAAAAAAACCGCAAAAATGGA

79

CCTCTGTACACCTTCGGCGGCTGGTTCCATAAAGGTTACCTCACCGACTTCGTTGGTTACAAACTCTCGTGGTGTGACGGCCGGTGTGTACAAGACCCGGAAACGTATTCACCGTGGCGTGCTGATCCACGATTACTAGCGATTCCGGCTTCATGCAGGCGAGTTGCAGCCTGCAATCCGAACTGAGAATGGCTTTAAGAGATTTGCTTGCCCTCGCGGGTTTGCGACTCGTTGTACCATCCATTGTAGCACGTGGGTAGCCCAAGTCATAAGGGGCATGCTGATTTGACGTCATCCCCACCTTCCTCCGGTTTGTCACCGGCAGTCTCATTAAAGTGCCCAACTAAATGCTGGCAACTAATAATAGGGGTTGCGCTCGTTGCGGGACTTAACCCAACATCTCACGACACGAGCTGACGACAACCATGCACCACCTGTCACTTTGTCCCCGAAGGGAAAGTCCTATCTCTAGGGTGTTCAAAGGATGTCAAGACTTGGTAAGGTTCTTCGCGTTGCTTCGAATTAAACCACATGCTCCACCGCTTGTGCGGGTCCCCGTCAATTCCTTTGAGTTTCAACCTTGCGGTCGTACTCCCCAGGCGGAGTGCTTAATGCGTTAACTGCGGAACTGAAGGCTGGAAACCCTCCAACACCTAGCACTCATCGTTTACGGCGTGAACTACCAGGGTATCTAATCCTGTTTGCTCCCCCACGCTTTCGAGCCTCAGTGTCAGTAACAGACCAGAATGTCGCCTTCGCCACTGAGTTCTTCCATATATCTACGCATTCCACCCGCTACACATGGAGTTCCACATTCCCTCATCATGAACTCAAGTTTCCCAGATTACCAAATGAGCCCCCTCCCAACGTGTAAGGCCCGGTGGGGACTTTTCAC

80

CCTCTGATAGCTTCGGCGGCTGGCTCCTTACGGTTACCTCACCGACTTCGGGTGTTGCAAACTCTCGTGGTGTGACGGGCGGTGTGTACAAGACCCGGGAACGTATTCACCGCAGTATGCTGACCTGCGATTACTAGCGATTCCGACTTCATGCAGGCGAGTTGCAGCCTGCAATCCGAACTGAGAACGGCTTTCTGGGATTGGCTCCACCTCGCGGCTTCGCTGCCCTTTGTACCGTCCATTGTAGCACGTGTGTAGCCCAACTCATAAGGGGCATGATGATTTGACGTCATCCCCACCTTCCTCCGGTTTGTCACCGGCAGTCTCCCTAGAGTGCCCAACCAAATGCTGGCAACTAAGGACAAGGGTTGCGCTCGTTGCGGGACTTAACCCAACATCTCACGACACGAGCTGACGACAACCATGCACCACCTGTCACCCCTGCCCCCGAAGGGGAAGGTACATCTCTGTACCGGTCAGGGGGATGTCAAGAGTTGGTAAGGTTCTTCGCGTTGCTTCGAATTAAACCACATGCTCCACCGCTTGTGCGGGTCCCCGTCAATTCCTTTGAGTTTCAGCCTTGCGACCGTACTCCCCAGGCGGAGTGCTTAATGCGTTAGCTTCAGCACTGAAGGGCGGAAACCCTCCAACACCTAGCACTCATCGTTTACGGCGTGGACTACCAGGGTATCTAATCCTGTTTGCTCCCCACGCTTTCGCGCCTCAGCGTCAGTTATAGGCCAAAGAGTCGCCTTCGCCACTGGTGTTCCTCCACATCTCTACGCATTTCACCGCTACACGTGGAATTCCACTCTTCTCTCCTATACTCAAGCCTCCCAGTTTCCAATGGCCCTCCCCGGTTGAGCCGGGGGCTTTCACATCAGACTTAAGAGGCCGCCTGCGCGCGCTTTACGCCCAATAATTCCGGAAACGCTTGGCACCTA

81

ATTTTGTGACCTTCGACGGCTAGCTCCAAAATGGTTACTCCACCGGCTTCGGGTGTTACAAACTCTCGTGGTGTGACGGGCGGTGTGTACAAGACCCGGGAACGTATTCACCGTAGCATGCTGATCTACGATTACTAGCGATTCCAGCTTCATGTAGTCGAGTTGCAGACTACAATCCGAACTGAGAACAACTTTATGGGATTTGCTTGACCTCGCGGTTTCGCTGCCCTTTGTATTGTCCATTGTAGCACGTGTGTAGCCCAAATCATAAGGGGCATGATGATTTGACGTCATCCCCACCTTCCTCCGGTTTGTCACCGGCAGTCAACTTAGAGTGCCCAACTGAATGATGGCAACTAAGTTCAAGGGTTGCGCTCGTTGCGGGACTTAACCCAACATCTCACGACACGAGCTGACGACAACCATGCACCACCTGTCACTTTGTCCTCCGAAGAGGAAAACTCTATCTCTAGAGCGGTCAAAGGATGTCAAGATTTGGTAAGGTTCTTCGCGTTGCTTCGAATTAAACCACATGCTCCACCGCTTGTGCGGGTCCCCGTCAATTCCTTTGAGTTTCAGTCTTGCGACCGTACTCCCCAGGCGGAGTGCTTAATGCGTTAGCTGCAGCACTAAGGGGCGGAAACCCCCTAACACTTAGCACTCATCGTTTACGGCGTGGACTACCAGGGTATCTAATCCTGTTTGATCCCCACGCTTTCGCACATCAGCGTCAGTTGCAGACCAGAAAGCCGCCTTCGCCACTGGTGTTCCTCCATATCTCTGCGCATTTCACCGCTACACATGGAATTCCACTTTCCTCTTCTGCACTCAAGTTTTCCAGTTTCCAATGACCCTCCACGGTTGAGCCGTGGGCTTTCACATCAGACTTAAAAAACCGCCTACGCGCGTTTACGCCCATAATTCCGGATAACGCTTGCCCCTACGTTTACCGGGCTGGTGGCACGAATTAGCCGGGGTTTTTGGGTAGGA

83

CAGTGATACCAAGTGGTAGCGCCCTCCCGAAGGTTAAGCTACCTACTTCTTTTGCAACCCACTCCCATGGTGTGACGGGCGGTGTGTACAAGGCCCGGGAACGTATTCACCGTGGCATTCTGATCCACGATTACTAGCGATTCCGACTTCATGGAGTCGAGTTGCAGACTCCAATCCGGACTACGACGCACTTTATGAGGTCCGCTTGCTCTCGCGAGGTCGCTTCTCTTTGTATGCGCCATTGTAGCACGTGTGTAGCCCTGGTCGTAAGGGCCATGATGACTTGACGTCATCCCCACCTTCCTCCAGTTTATCACTGGCAGTCTCCTTTGAGTTCCCGGCCGGACCGCTGGCAACAAAGGATAAGGGTTGCGCTCGTTGCGGGACTTAACCCAACATTTCACAACACGAGCTGACGACAGCCATGCAGCACCTGTCTCACGGTTCCCGAAGGCACATTCTCATCTCTGAAAACTTCCGTGGATGTCAAGACCAGGTAAGGTTCTTCGCGTTGCATCGAATTAAACCACATGCTCCACCGCTTGTGCGGGCCCCCGTCAATTCATTTGAGTTTTAACCTTGCGGCCGTACTCCCCAGGCGGTCGACTTAACGCGTTAGCTCCGGAAGCCACGCCTCAAGGGCACAACCTCCAAGTCGACATCGTTTACGGCGTGGACTACCAGGGTATCTAATCCTGTTTGCTCCCCACGCTTTCGCACCTGAGCGTCAGTCTTCGTCCAGGGGGCCGCCTTCGCCACCGGTATTCCTCCAGATCTCTACGCATTTCACCGCTACACCTGGAATTCTACCCCCCTCTACGAGACTCAAGCTTGCCAGTATCAGATGCAGTTCCCAGGTTGAGCCCGGGGATTCACATCTGACTAACAAACCGCCTGCGTGCGTTTACGCCCAGTAATTCCGATAACGCTTGCCCCTCCGATTACCGCGGTGCTGGACGGAGTAGCCGGGGTTCTTCTGCGGGACGGCATGGGCAAGGTTTAACTTATCCCTTCTCCCCGTGAAAGATTTAAACCCAAGGCTCTTCTAACCGGGA

84

CCTCAGTAACCTTAGGCGGCTGGCTCCTAATAGGTTACCTCACCGACTTCGGGTGTTACAAACTCTCGTGGTGTGACGGGCGGTGTGTACAAGGCCCGGGAACGTATTCACCGCGGCGTGCTGATCCGCGATTACTAGCGATTCCGACTTCATGTAGGCGAGTTGCAGCCTACAATCCGAACTGAGATTGGCTTTCAGAGATTAGCTTGCCGTCACCGGCTTGCGACTCGTTGTACCAACCATTGTAGCACGTGTGTAGCCCAGGTCATAAGGGGCATGATGATTTGACGTCATCCCCACCTTCCTCCGGTTTATTACCGGCAGTCTCGCTAGAGTGCCCAACTTAATGATGGCAACTAACAATAGGGGTTGCGCTCGTTGCGGGACTTAACCCAACATCTCACGACACGAGCTGACGACAACCATGCACCACCTGTCACCGATGTACCGAAGTAAAACTCTATCTCTAGAGCGGGCATCGGGATGTCAAGACCTGGTAAGGTTCTTCGCGTTGCTTCGAATTAAACCACATGCTCCACCGCTTGTGCGGGCCCCCGTCAATTCCTTTGAGTTTCAACCTTGCGGTCGTACTCCCCAGGCGGAGTGCTTAATGCGTTAGCTCCGGCACTAAGCCCCGGAAAGGGCCTAACACCTAGCACTCATCGTTTACGGCGTGGACTACCAGGGTATCTAATCCTGTTTGCTCCCCACGCTTTCGAGCCTCAGCGTCAGTTACAGACCAGAGAGCCGCTTTCGCCACCGGTGTTCCTCCATATATCTACGCATTTCACCGCTACACATGGAATTCCACTCTCCCCTTCTGCACTCAAGTTCTCCAGTTTCCAAAGCATACATTGGTTGAGCCAATGCCTTTAACTTCAGACTTAAAGAACCGCCTGCGCTCGCTTTACGCCCATAAATCCGGACACGCTCGGGACCTACTATTACCGCGGTGGTGGGACGAA

85

CCGTTGGTGACCTTCGACGGCTAGCTCCAAAATGGTTACTCCACCGGCTTCGGGTGTTACAAACTCTCGTGGTGTGACGGGCGGTGTGTACAAGACCCGGGAACGTATTCACCGTAGCATGCTGATCTACGATTACTAGCGATTCCAGCTTCATGTAGTCGAGTTGCAGACTACAATCCGAACTGAGAACAACTTTATGGGATTTGCTTGACCTCGCGGTTTCGCTGCCCTTTGTATTGTCCATTGTAGCACGTGTGTAGCCCAAATCATAAGGGGCATGATGATTTGACGTCATCCCCACCTTCCTCCGGTTTGTCACCGGCAGTCAACTTAGAGTGCCCAACTGAATGATGGCAACTAAGTTCAAGGGTTGCGCTCGTTGCGGGACTTAACCCAACATCTCACGACACGAGCTGACGACAACCATGCACCACCTGTCACTTTGTCCTCCGAAGAGGAAAACTCTATCTCTAGAGCGGTCAAAGGATGTCAAGATTTGGTAAGGTTCTTCGCGTTGCTTCGAATTAAACCACATGCTCCACCGCTTGTGCGGGTCCCCGTCAATTCCTTTGAGTTTCAGTCTTGCGACCGTACTCCCCAGGCGGAGTGCTTAATGCGTTAGCTGCAGCACTAAGGGGCGGAAACCCCCTAACACTTAGCACTCATCGTTTACGGCGTGGACTACCAGGGTATCTAATCCTGTTTGATCCCCACGCTTTCGCACATCAGCGTCAGTTGCAGACCAGAAAGCCGCCTTCGCCACTGGTGTTCCTCCATATCTCTGCGCATTTCACCGCTACACATGGAATTCCACTTTCCTCTTCTGCACTCAAGTTTTCCAGTTTCCAATGACCCTCCACGGTTGAGCCGTGGGCTTTCACATCAGACTTAAAAAACCGCCTACGCGCGCTTTACGCCCATAATTCCGGATAACGCTTGCCCCTACGTTTACCGCGCTGCTG

86

CGCGTGATCCAAGTGGTAGCGCCCTCCCGAAGGTTAAGCTACCTACTTCTTTTGCAACCCACTCCCATGGTGTGACGGGCGGTGTGTACAAGGCCCGGGAACGTATTCACCGTGGCATTCTGATCCACGATTACTAGCGATTCCGACTTCATGGAGTCGAGTTGCAGACTCCAATCCGGACTACGACGCACTTTATGAGGTCCGCTTGCTCTCGCGAGGTCGCTTCTCTTTGTATGCGCCATTGTAGCACGTGTGTAGCCCTGGTCGTAAGGGCCATGATGACTTGACGTCATCCCCACCTTCCTCCAGTTTATCACTGGCAGTCTCCTTTGAGTTCCCGGCCGGACCGCTGGCAACAAAGGATAAGGGTTGCGCTCGTTGCGGGACTTAACCCAACATTTCACAACACGAGCTGACGACAGCCATGCAGCACCTGTCTCACGGTTCCCGAAGGCACATTCTCATCTCTGAAAACTTCCGTGGATGTCAAGACCAGGTAAGGTTCTTCGCGTTGCATCGAATTAAACCACATGCTCCACCGCTTGTGCGGGCCCCCGTCAATTCATTTGAGTTTTAACCTTGCGGCCGTACTCCCCAGGCGGTCGACTTAACGCGTTAGCTCCGGAAGCCACGCCTCAAGGGCACAACCTCCAAGTCGACATCGTTTACGGCGTGGACTACCAGGGTATCTAATCCTGTTTGCTCCCCACGCTTTCGCACCTGAGCGTCAGTCTTCGTCCAGGGGGCCGCCTTCGCCACCGGTATTCCTCCAGATCTCTACGCATTTCACCGCTACACCTGGAATTCTACCCCCCTCTACGAGACTCAAGCTTGCCAGTATCAGATGCAGTTCCCAGGTTGAGCCCGGGGATTTCACATCTGACTTAACAAACCGCCTGCGTGCGCTTTACGCCCAGTAATTCCGATTAACGCTTGCACCTCCGGATTACCGCGGTGGTGGCACGGAGTTAGCCGGGGTTCTTCTGCGGGAACGCCATGG

87

CACTGATACAAGTGGTAAGCGCCCTCCCGAAGGTTAAGCTACCTACTTCTTTTGCAACCCACTCCCATGGTGTGACGGGCGGTGTGTACAAGGCCCGGGAACGTATTCACCGTGGCATTCTGATCCACGATTACTAGCGATTCCGACTTCATGGAGTCGAGTTGCAGACTCCAATCCGGACTACGACGCACTTTATGAGGTCCGCTTGCTCTCGCGAGGTCGCTTCTCTTTGTATGCGCCATTGTAGCACGTGTGTAGCCCTGGTCGTAAGGGCCATGATGACTTGACGTCATCCCCACCTTCCTCCAGTTTATCACTGGCAGTCTCCTTTGAGTTCCCGGCCGGACCGCTGGCAACAAAGGATAAGGGTTGCGCTCGTTGCGGGACTTAACCCAACATTTCACAACACGAGCTGACGACAGCCATGCAGCACCTGTCTCACGGTTCCCGAAGGCACATTCTCATCTCTGAAAACTTCCGTGGATGTCAAGACCAGGTAAGGTTCTTCGCGTTGCATCGAATTAAACCACATGCTCCACCGCTTGTGCGGGCCCCCGTCAATTCATTTGAGTTTTAACCTTGCGGCCGTACTCCCCAGGCGGTCGACTTAACGCGTTAGCTCCGGAAGCCACGCCTCAAGGGCACAACCTCCAAGTCGACATCGTTTACGGCGTGGACTACCAGGGTATCTAATCCTGTTTGCTCCCCACGCTTTCGCACCTGAGCGTCAGTCTTCGTCCAGGGGGCCGCCTTCGCCACCGGTATTCCTCCAGATCTCTACGCATTTCACCGCTACACCTGGAATTCTACCCCCCTCTACGAGACTCAAGCTTGCCAGTATCAGATGCAGTTCCCAGGTTGAGCCCGGGGATTCACATCTGACTTAACAAACCGCCTGCGTGCGCTTTACGCCCAGTAATTCCGATTAACGCTTGCACCCTCCGGATTACCGCGGTGGTGGCACGGAGT

88

ACTTTGTGACCTTCGACGGCTAGCTCCAAAAGGGTTACTCCACCGGTTTCGGGGGTTACAAACTCTCGGGGGGTGACGGGGGGGGGGTACAAAACCCGGAAACGTATTCACCGTAGCATGCTGATCTACGATTACTAGCGATTCCAGCTTCATGTAGCCGAGTTGCAAACTACAATCCGAACTGAAAACAACTTTATGGAATTTGCTTGACCTCGCGGTTTCGTTGCCCTTTGTATTGTCCATTGTAGCACGTGTGTAGCCCAAATCATAAGGGGCATGATGATTTGACGTCATCCCCACCTTCCTCCGGTTTGTCACCGGCAGCCAACTTAAAGGGCCCAACTTAAGGATGGCAACTAAGCTCAAGGGTTGCGCTCGTTGCGGAACTTAACCCAACATCTCACGACACGAGCTGACGACAACCATGCACCACCTGTCACTTTGTCCTCCGAAAAGGAAAACTCTATCTCTAAAGCGGCCAAAGGATGTCAAGATTTGGAAAGGTTCTTCGCGTTGCTTCAAATTAAACCACATGCTCCACCGCTTGTGCGGGTCCCCGTCAATTCCTTTGAGTTTCAGCCTTGCGACCGTACTCCCCAGGCGAAGTGCTTAATGCGTTAGCTGCAGCACTAAGGGGCGAAAACCCCCTAACACTTAGCACTCATCGTTTACGGCGTGGACTACCAGGGTATCTAATCCTGTTTGATCCCCACGCTTTCGCACATCAGCGTCAGTTGCAGACCAGAAAGCCGCCTTCGCCACTGGTGTTCCTCCATATCTCTGCGCATTTTCACCGCTACACATGGAATTCCACTTTCTCTCTGCACTCAGTTTCCCAGGTTTCCATGACCCCTCCCCACGGGTTGGAGCCGGGTGGGGGCCTTTTCA

89

CAGTATCCAAGTGGTAGCGCCCTCCCGAAGGTTAAGCTACCTACTTCTTTTGCAACCCACTCCCATGGTGTGACGGGCGGTGTGTACAAGGCCCGGGAACGTATTCACCGTGGCATTCTGATCCACGATTACTAGCGATTCCGACTTCATGGAGTCGAGTTGCAGACTCCAATCCGGACTACGACGCACTTTATGAGGTCCGCTTGCTCTCGCGAGGTCGCTTCTCTTTGTATGCGCCATTGTAGCACGTGTGTAGCCCTGGTCGTAAGGGCCATGATGACTTGACGTCATCCCCACCTTCCTCCAGTTTATCACTGGCAGTCTCCTTTGAGTTCCCGGCCGGACCGCTGGCAACAAAGGATAAGGGTTGCGCTCGTTGCGGGACTTAACCCAACATTTCACAACACGAGCTGACGACAGCCATGCAGCACCTGTCTCACGGTTCCCGAAGGCACATTCTCATCTCTGAAAACTTCCGTGGATGTCAAGACCAGGTAAGGTTCTTCGCGTTGCATCGAATTAAACCACATGCTCCACCGCTTGTGCGGGCCCCCGTCAATTCATTTGAGTTTTAACCTTGCGGCCGTACTCCCCAGGCGGTCGACTTAACGCGTTAGCTCCGGAAGCCACGCCTCAAGGGCACAACCTCCAAGTCGACATCGTTTACGGCGTGGACTACCAGGGTATCTAATCCTGTTTGCTCCCCACGCTTTCGCACCTGAGCGTCAGTCTTCGTCCAGGGGGCCGCCTTCGCCACCGGTATTCCTCCAGATCTCTACGCATTTCACCGCTACACCTGGAATTCTACCCCCCTCTACGAGACTCAAGCTTGCCAGTATCAGATGCAGTTCCCAGGTTGAGCCCGGGGATTCACATCTGACTTAACAAACCGCCTGCGTGCGTTTACGCCCAGTAATTCCGATAACGCTTGCCCCTCCGATTACCGCGGTGCTGGACGGAGTAGCCGGGGTTCTTCTGCGGTAACGCCATG

90

ATCGATCCACCTTAGGCGGCTGGCTCCTAATAGGTTACCTCACCGACTTCGGGTGTTACAAACTCTCGTGGTGTGACGGGCGGTGTGTACAAGGCCCGGGAACGTATTCACCGCGGCGTGCTGATCCGCGATTACTAGCGATTCCGACTTCATGTAGGCGAGTTGCAGCCTACAATCCGAACTGAGATTGGCTTTCAGAGATTAGCTTGCCGTCACCGGCTTGCGACTCGTTGTACCAACCATTGTAGCACGTGTGTAGCCCAGGTCATAAGGGGCATGATGATTTGACGTCATCCCCACCTTCCTCCGGTTTATTACCGGCAGTCTCGTTAAAGTGCCCAACTTAATGATGGCAACTAACAATAGGGGTTGCGCTCGTTGCGGGACTTAACCCAACATCTCACGACACGAGCTGACGACAACCATGCACCACCTGTCACCGATGTACCGAAGTAAAACTCTATCTCTAGAGCGGGCATCGGGATGTCAAGACCTGGTAAGGTTCTTCGCGTTGCTTCGAATTAAACCACATGCTCCACCGCTTGTGCGGGCCCCCGTCAATTCCTTTGAGTTTCAACCTTGCGGTCGTACTCCCCAGGCGGAGTACTTAATGCGTTAGCTCCGGAACTAAGCCCCGGAAAGGGCCTAACACCTAGCACTCATCGTTTACGGCGTGAACTACCAGGGTATCTAATCCTGTTTGCTCCCCCACGCTTTCGAGCCTCAGCGTCAGCTACAGACCAGAGAGCCGCTTTCGCCACCGATGTTCCTCCATATATCTACGCATTTCACCGCTACACATGGAATTTCCACTCTCCCATCATGAACTCAGGTGCTTCAGATTTCATAGCATACATAGTGAGCAATGCCTTTCACA

91

TGAATACCAAAGTGGTAGCGCCCTCCCGAAGGTTAAGCTACCTACTTCTTTTGCAACCCACTCCCATGGTGTGACGGGCGGTGTGTACAAGGCCCGGGAACGTATTCACCGTGGCATTCTGATCCACGATTACTAGCGATTCCGACTTCATGGAGTCGAGTTGCAGACTCCAATCCGGACTACGACGCACTTTATGAGGTCCGCTTGCTCTCGCGAGGTCGCTTCTCTTTGTATGCGCCATTGTAGCACGTGTGTAGCCCTGGTCGTAAGGGCCATGATGACTTGACGTCATCCCCACCTTCCTCCAGTTTATCACTGGCAGTCTCCTTTGAGTTCCCGGCCGGACCGCTGGCAACAAAGGATAAGGGTTGCGCTCGTTGCGGGACTTAACCCAACATTTCACAACACGAGCTGACGACAGCCATGCAGCACCTGTCTCACGGTTCCCGAAGGCACATTCTCATCTCTGAAAACTTCCGTGGATGTCAAGACCAGGTAAGGTTCTTCGCGTTGCATCGAATTAAACCACATGCTCCACCGCTTGTGCGGGCCCCCGTCAATTCATTTGAGTTTTAACCTTGCGGCCGTACTCCCCAGGCGGTCGACTTAACGCGTTAGCTCCGGAAGCCACGCCTCAAGGGCACAACCTCCAAGTCGACATCGTTTACGGCGTGGACTACCAGGGTATCTAATCCTGTTTGCTCCCCACGCTTTCGCACCTGAGCGTCAGTCTTCGTCCAGGGGGCCGCCTTCGCCACCGGTATTCCTCCAGATCTCTACGCATTTCACCGCTACACCTGGAATTCTACCCCCCTCTACGAGACTCAAGCTTGCCAGTATCAGATGCAGTTCCCAGGTTGAGCCCGGGGATTCACATCTGACTTAACAAACCGCCTGCGTGCGCTTTACGCCCAGTAATTCCGATTAACGCTTGCCCCTCCGAATTACCGCGGCTGCTGGCACGGAGTTAGCCGGGGTTCTTCTGCGGGAAC

92

CACCGATACTCCGTGGTACCGTCCCCCTTGCGGTTAGACTAGCTACTTCTGGAGCAACCCACTCCCATGGTGTGACGGGCGGTGTGTACAAGGCCCGGGAACGTATTCACCGTGACATTCTGATTCACGATTACTAGCGATTCCGACTTCACGCAGTCGAGTTGCAGACTGCGATCCGGACTACGATCGGTTTTATGGGATTAGCTCCACCTCGCGGCTTGGCAACCCTTTGTACCGACCATTGTAGCACGTGTGTAGCCCTGGCCGTAAGGGCCATGATGACTTGACGTCATCCCCACCTTCCTCCGGTTTGTCACCGGCAGTCTCCTTAGAGTGCCCACCCGAGGTGCTGGTAACTAAGGACAAGGGTTGCGCTCGTTACGGGACTTAACCCAACATCTCACGACACGAGCTGACGACAGCCATGCAGCACCTGTGTCTGAGTTCCCGAAGGCACCAATCCATCTCTGGAAAGTTCTCAGCATGTCAAGGCCAGGTAAGGTTCTTCGCGTTGCTTCGAATTAAACCACATGCTCCACCGCTTGTGCGGGCCCCCGTCAATTCATTTGAGTTTTAACCTTGCGGCCGTACTCCCCAGGCGGTCGACTTATCGCGTTAGCTGCGCCACTAAGATCTCAAGGATCCCAACGGCTAGTCGACATCGTTTACGGCGTGGACTACCAGGGTATCTAATCCTGTTTGCTCCCCACGCTTTCGCACCTCAGTGTCAGTATCAGTCCAGGTGGTCGCCTTCGCCACTGGTGTTCCTTCCTATATCTACGCATTTCACCGCTACACAGGAAATTCCACCACCCTCTACCGTACTCTAGCTCAGTAGTTTTGGATGCAGTTCCCAGGTTGAGCCCGGGGATTCACATCCAACTTGCTGAACCACCTACGCGCGCTTTACGCCCAGTAATTCCGATTAACGCTTGGACCCTTCGAATTACCGCGGTGGTGGCCCAAATTAACCGGGGTTATTC

93

GGTCCACCTTCGACGGCTAGCTCCAAAATGGTTACTCCACCGGCTTCGGGTGTTACAAACTCTCGTGGTGTGACGGGCGGTGTGTACAAGACCCGGGAACGTATTCACCGTAGCATGCTGATCTACGATTACTAGCGATTCCAGCTTCATGTAGTCGAGTTGCAGACTACAATCCGAACTGAGAACAACTTTATGGGATTTGCTTGACCTCGCGGTTTCGCTGCCCTTTGTATTGTCCATTGTAGCACGTGTGTAGCCCAAATCATAAGGGGCATGATGATTTGACGTCATCCCCACCTTCCTCCGGTTTGTCACCGGCAGTCAACTTAGAGTGCCCAACTGAATGATGGCAACTAAGTTCAAGGGTTGCGCTCGTTGCGGGACTTAACCCAACATCTCACGACACGAGCTGACGACAACCATGCACCACCTGTCACTTTGTCCTCCGAAGAGGAAAACTCTATCTCTAGAGCGGTCAAAGGATGTCAAGATTTGGTAAGGTTCTTCGCGTTGCTTCGAATTAAACCACATGCTCCACCGCTTGTGCGGGTCCCCGTCAATTCCTTTGAGTTTCAGTCTTGCGACCGTACTCCCCAGGCGGAGTGCTTAATGCGTTAGCTGCAGCACTAAGGGGCGGAAACCCCCTAACACTTAGCACTCATCGTTTACGGCGTGGACTACCAGGGTATCTAATCCTGTTTGATCCCCACGCTTTCGCACATCAGCGTCAGTTGCAGACCAGAAAGCCGCCTTCGCCACTGGTGTTCCTCCATATCTCTGCGCATTTCACCGCTACACATGGAATTCCACTTTCCTCTTCTGCACTCAAGTTTTCCAGTTTCCAATGACCCTCCACGGTTGAGCCGTGGGCTTTCACATCAGACTTAAAAAACCGCCTACGCGCGCTTTACGCCCATAATTCCGGAAACGCTTGCCCCTACGTATACCGCGGTGCTGGA
