# Supplementary material for: Phage therapy against methicillin-resistant Staphylococcus pseudintermedius: a novel strategy for canine pyoderma
Source: Front Microbiol. 2026 Jan 13;16:1719973. doi: 10.3389/fmicb.2025.1719973 (PMC12835223; doi:10.3389/fmicb.2025.1719973)
Supplement: Supplementary file 5 [file Table_5.docx]

Prediction of the major functional proteins of phage P32

| Function | Name |
| --- | --- |
| Protein structure module | portal protein; head maturation protease; major capsid protein; Putative head tail adaptor; tail component; major tail protein; tail length tape measure protein; phage tail protein; transmembrane protein |
| DNA Replication and Metabolic Modules | Phosphoprotein; integrase; Repressor protein CI; helix-turn-helix transcriptional regulator; exonuclease; endonuclease; Gp2.5-like ssDNA binding protein and ssDNA annealing protein; DNA polymerase; DNA helicase; nucleotide kinase; dUTP diphosphatase; DNA methyltransferase; transcriptional regulator; transcriptional activator RinB |
| DNA packaging module | terminase small subunit; terminase large subunit |
| Cracking module | Holin; endolysin） |
| Family of unknown function proteins | DUF2951 family protein; DUF1514 family protein |
