# Supplementary material for: Phage therapy against methicillin-resistant Staphylococcus pseudintermedius: a novel strategy for canine pyoderma
Source: Front Microbiol. 2026 Jan 13;16:1719973. doi: 10.3389/fmicb.2025.1719973 (PMC12835223; doi:10.3389/fmicb.2025.1719973)
Supplement: Supplementary file 6 [file Table_6.docx]

Prediction of the major functional proteins of phage DW

| Function | Name |
| --- | --- |
| Protein structural module | minor structural protein; tail protein; tail length tape measure protein; major tail protein; Putative head tail adaptor; major capsid protein; head maturation protease |
| DNA replication and metabolism module | ribonucleoside-diphosphate reductase subunit alpha 1; ribonucleotide reductase alpha subunit; Mom-like DNA modification protein; ribonucleotide reductase class Ia beta subunit; DNA helicase; ribonuclease HI; HNH endonuclease; PhoH-related protein; YopX family protein; dimeric dUTPase; DNA polymerase III alpha subunit; guanylate kinase; integrase |
| DNA packaging module | terminase large subunit |
| Cracking module | Holin; lysin N-acetylmuramoyl-L-alanine amidase |
