# Supplementary material for: Phage therapy against methicillin-resistant Staphylococcus pseudintermedius: a novel strategy for canine pyoderma
Source: Front Microbiol. 2026 Jan 13;16:1719973. doi: 10.3389/fmicb.2025.1719973 (PMC12835223; doi:10.3389/fmicb.2025.1719973)
Supplement: Supplementary file 7 [file Table_7.docx]

Prediction of the major functional proteins of phage YX

| Function | Name |
| --- | --- |
| Protein structural module | head maturation protease; major capsid protein; Putative head tail adaptor; major tail protein; tail length tape measure protein; tail protein; minor structural protein |
| DNA replication and metabolism module | guanylate kinase; DNA polymerase III alpha subunit; dimeric dUTPase; YopX family protein; PhoH-related protein; HNH endonuclease; ribonuclease HI; DNA helicase; ribonucleotide reductase class Ia beta subunit; Mom-like DNA modification protein; ribonucleoside-diphosphate reductase subunit alpha 1; ribonucleotide reductase alpha subunit |
| DNA packaging module | terminase large subunit |
| Cracking module | Holin; lysin N-acetylmuramoyl-L-alanine amidase |
